# Boron Tetrafluoride Anion Bonding Dual Active Species Within a Large–Pore Mesoporous Silica for Two–Step Successive Organic Transformaion to Prepare Optically Pure Amino Alcohols

**DOI:** 10.3389/fchem.2018.00272

**Published:** 2018-07-06

**Authors:** Liang Li, Dongfeng Yang, Zhongrui Zhao, Yongkang Song, Lei Zhao, Rui Liu, Guohua Liu

**Affiliations:** Key Laboratory of Resource Chemistry of Ministry of Education, Shanghai Key Laboratory of Rare Earth Functional Materials, Shanghai Normal University, Shanghai, China

**Keywords:** asymmetric catalysis, heterogeneous catalyst, tandem reaction, silica, anion bonding

## Abstract

Development of a simple and easy handing process for preparation of multifunctional heterogenous catalysts and exploration of their applications in sequential organic transformation are of great significance in heterogeneous asymmetric catalysis. Herein, through the utilization of a ${\text{BF}}_{4}^{-}$ anion–bonding strategy, we anchor conveniently both organic bases and chiral ruthenium complex into the nanopores of Me-FDU−12, fabricating a Lewis base/Ru bifunctional heterogeneous catalyst. As we envisaged, cyclic amine as a Lewis base promotes an intermolecular aza–Michael addition between enones and arylamines, affording γ-secondary amino ketones featuring with aryl motif, whereas ruthenium/diamine species as catalytic promoter boosts an asymmetric transfer hydrogenation of γ-secondary amino ketones to γ-secondary amino alcohols. As expected, both enhance synergistically the aza–Michael addition/asymmetric transfer hydrogenation one–pot enantioselective organic transformation, producing chiral γ-secondary amino alcohols with up to 98% enantioselectivity. Unique features, such as operationally simple one–step synthesis of heterogeneous catalyst, homo–like catalytic environment as well as green sustainable process make this heterogeneous catalyst an attracting in a practical preparation of optically pure pharmaceutical intermediates of antidepressants.

## Introduction

With the extensive explorations of mesoporous silicas as supports in catalysis, various strategies have been employed to construct supported molecular catalysts(Song, 2005; Heitbaum et al., 2006; Yang et al., 2007; Margelefsky et al., 2008; Thomas and Raja, 2008; Bartók, 2010; Yu and He, 2012). Of all the well–established strategies, immobilization though a hydrogen–bonding have attracting a great deal of interest since it allow the facile anchoring of small molecular catalysts on a support with a suitable counterion of organometallic complexes (Fraile et al., 2009). This simple and practical approach endows the immobilized catalyst with an original catalytic environment as its homogeneous counterpart which is helpful for the catalytic reaction. These features are beneficial to construct highly efficient heterogeneous catalysts like their corresponding homogeneous counterparts, bridging the gap between homogeneous and heterogeneous catalysis. Recently, some remarkable works developed by several groups have utilized F_3_${\text{CSO}}_{3}^{-}$ anion to bond cationic complexes, fabricating various supported molecular catalysts with high catalytic efficiency (Bianchini et al., 2001; Raja et al., 2003; Rouzaud et al., 2003; O'Leary et al., 2004; Wang et al., 2006; McDonagh et al., 2007; Xu et al., 2012). These explorations overcome nicely the shortage of low catalytic efficiency of heterogeneous catalyst because of highly catalytic nature of cationic active species themselves, realizing superior catalytic performance relatively to their corresponding neutral counterparts. Similar to the F_3_${\text{CSO}}_{3}^{-}$ anion hydrogen–bonding method, recent reports also find that the ${\text{BF}}_{4}^{-}$ anions are used for fabrication of highly recyclable catalysts (Jones et al., 2003; Wiench et al., 2009; Shu et al., 2015; Liao et al., 2017; Xia et al., 2017). An outstanding example was reported by Toste and co–workers (Shu et al., 2015). In this work, the Au– complexes (Ph_3_PAuBF_4_) were tethered within the nanochannels of SBA−15 material *via* a hydrogen–bonding method between the ${\text{BF}}_{4}^{-}$ anion and the inner silanols of SBA−15. This as–made heterogeneous cationic gold (I) catalyst not only performs a superior regioselectivity to its corresponding homogeneous one in the cyclization of alkynoic acids. More importantly, through the suitable coordination with chiral phosphine ligands, the obtained chiral cationic gold (I) catalyst also shows the dramatic enhancement of enantioselectivities in the enantioselective lactonization reactions relative to its homogeneous one. In particular, high recyclability (11 runs) opens great potential in a practical application. Quite recently, we also take advantage of this ${\text{BF}}_{4}^{-}$ anion–bonding method to bond Au/carbine complexes, together with a covalent–bonding of neutral Ru/diamine complexes, to fabricate a recyclable heterogeneous catalysts for the enantioselective synthesis of halohydrins through a hydration/asymmetric transfer hydrogenation cascade process (Xia et al., 2017). Therefore, further development of this easy handing hydrogen–bonding method and direct immobilization of dual active centers on mesoporous silica for multi–step sequential organic transformation is still of great significance in heterogeneous asymmetric catalysis.

Large–pore mesoporous silicas, like FDU−12 (Fan et al., 2005; Ma et al., 2010; Li et al., 2015), possess large size of regular mesopores in their silicate networks, which have great potential in assembly of various heterogeneous catalysts (Li et al., 2012; Yan et al., 2013; Chen et al., 2014). At first, large space of nanopores can accommodate large steric organometallic complexes or multiply active species, which are beneficial to fabricate bi– or multi–functionalized heterogeneous catalysts. Also, large space of mesopores can avoid the accumulation of multiple active species, which can reduce efficiently the cross–interactions of multiple active species and overcome the shortage of homogeneous catalysis. Furthermore, large size of regular mesopores benefits mass transport, which can lead to an improved catalytic efficiency relative to those small–sizes of mesoporous counterparts. More importantly, through a control of molecular catalysts in an adjacent position on a support, a potential cooperative effect may make some unfeasible or lowly efficient tandem reactions in homogeneous condition into possibility. Therefore, the utilization of advantages of large–pore mesoporous FDU−12 in fabrication of multifunctional catalyst and the exploration of its application in a tandem reaction to promote reactivity, enantioselectivity and stability are highly desirable.

In this contribution, we employ a large–pore size of FDU−12 as a support and assemble a Lewis base and a chiral ruthenium/diamine dual species within its regular nanopores *via* a ${\text{BF}}_{4}^{-}$ anion–bonding approach, fabricating a bifunctional heterogeneous catalyst. This catalyst performs an efficient synthesis of valuable pharmaceutical intermediates of optically pure γ-secondary amino alcohols that had been explored extensively by various asymmetric catalysis in homogeneous conditions recently (Gao and Sharpless, 1988; Robertson et al., 1988; Kakei et al., 2004; Liu et al., 2005; Fujima et al., 2006; Geng et al., 2011; Träff et al., 2011; Wang et al., 2013a, 2015; Zhou et al., 2014; Hu et al., 2015; Wu et al., 2017). Benefits of this heterogeneous catalysis not only overcome the drawbacks of expensive transition–metal recycle and transition–metal contamination in homogeneous catalysis system, but also realize multi-step reactions to yield a series of synthetically useful amino alcohols under mild reaction conditions in environmental friendly fashion.

## Experimental

### Catalyst 1 preparation

In a typical route, (Step I: *Synthesis of mesoporous FDU*−*12*) directing template EO_106_PO_70_EO_106_ (Pluronic F127, 0.5 g), 1,3,5–trimethylbenzene (TMB, 0.6 g) and KCl (1.25 g) were added to a hydrochloric acid solution (50 mL, 1.0 M) at 15°C. After stirring for 1.0 h, tetraethyl orthosilicate (TEOS, 2.08 g) was slowly added to previous solution. The resulting mixture was vigorously stirred for another 24 h at 15°C. Subsequently, the obtained suspension was kept in a Teflon–lined autoclave at 170°C for 24 h under static conditions. After cooling to room temperature, the as-made FDU-12 was seperated through filtration and dried. (Step II: *Preparation of mesoporous Me*–*FDU*−*12 by protection of the outer facial silanols*) The collected FDU-12 (1.0 g) were dispersed in 25 mL anhydrous toluene, and HMDS [(CH_3_)_3_Si)_2_N] (10 mL, 0.050 mol) was then added. The suspension was kept and stirred for 12 h at room temperature. After filtration and rinse with excess water and ethanol, the collected solids was redistributed to 120 mL of ammonium nitrate solution (80 mg (1.0 mmol) in 120 mL (95%) of ethanol), and the cloudy solution was stirred at 60°C for 10 h. Once finsihed, the solids were filtered and washed with excess water and ethanol, and dried under vacuo, affording trimethysilylated Me–FDU−12 as a white powder (1.21 g). (*Step III: Immobilization of (DABCO)BF*_4_ *(1–(chloromethyl)*−*1,4–diazabicyclo[2.2.2]octanium tetrafluoroborate*) *and (MesityleneRuTsDPEN)(BF*_4_*) (TsDPEN* = *N–((S,S)*−*2–amino*−*1,2–diphenylethyl)*−*4–methylbenzenesulfonamide)*. The Me–FDU−12 (0.50 g) was firstly dispersed into dry CH_2_Cl_2_ (10.0 mL) within a round–bottom flask at 25°C, then the freshly prepared [MesityleneRuTsDPEN] BF_4_ (43.50 mg, 0.064 mmol) was rapidly added. The resulting red suspension was stirred for 12 h at 25°C. After that, the newly prepared (DABCO)BF_4_ (74.42 mg, 0.30 mmol) was added to previous solution, and the reaction solution was stirred at 25°C for another 12 h. Once finished, the crude solid catalyst was obtained via filteration. Finally, the unreacted starting materials was removed by Soxhlet extraction using dry CH_2_Cl_2_, furnishing desired (DABCO)BF_4_@(MesityleneRuTsDPEN)BF_4_@Me–FDU−12 (**1**) (0.57 g) as a faint–yellow powder. The Ru loadings was determined by inductively coupled plasma optical emission spectrometer (ICP–OES) analysis, showing that 10.40 mg (0.1020 mmol of Ru) per gram of catalyst. Elemental analysis for the fresh catalyst **1** (%):C 15.03, H 3.43, N 0.57, S 0.33.; and for the recycled catalyst after fifth run: C 14.45, H 3.35, N 0.48, S 0.29. ^13^C CP/MAS NMR (161.9 MHz): 149.2–121.7 (C of Ph and Ar groups), 108.4, 101.5 (CH_3_ of Arene groups), 73.6–67.3 (CH of –NCH–Ph), 66.2–59.3 (CH_2_ of –N^+^CH_2_- in DABCO moiety, and CH_2_ of –OCH_2_CH_2_O– groups in F127), 53.2, 46.3 (CH_2_ of N(CH_2_)_3_- in DABCO moiety), 31.1 (CH_2_ of –CH_2_Ph), 23.3 (CH_3_ of mesitylene), 19.4 (CH_3_ of –OCH(CH_3_)CH_2_O– in F127), 2.8 (CH_3_ of –Si(CH_3_)_3_) ppm; ^29^Si MAS NMR (79.4 MHz): Q^3^ (δ = −104.8 ppm), Q^4^ (δ = −114.3 ppm).

### General procedure for 1-catlayzed aza–Michael addition/ATH one–pot enantioselective cascade reactions

In a typical procedure, catalyst **1** (19.61 mg, 2.0 μmol of Ru, according to ICP–OES analysis), HCO_2_Na (68.0 mg, 1.0 mmol), enones (0.10 mmol), amines (0.11 mmol) and 2.0 mL of co-solvents (H_2_O/^i^PrOH, v/v = 1/1) were successively added to a round bottom flask. The resulting mixture was heated to 40°C and stirred for 8–24 h until the fully consumption of enones. Upon completion of reaction (monitoring by TLC), the catalyst **1** was recycled *via* centrifugation (10,000 rpm). The transparent solution was extracted using ethyl acetate (3 × 3.0 mL). The combined organic phase were washed with brine (2 × 5 mL) and dried with Na_2_SO_4_. After removal of EtOAc under reduced evaporation, the crude residue was purified by flash column chromatography to release the desired products. The enantiomeric excess was determined by HPLC equipped with a Daicel chiralcel column (Ø 0.46 cm, L 25 cm) and an UV–Vis detector.

## Results and discussion

### Synthesis and structural characterization

**Figure 1:**
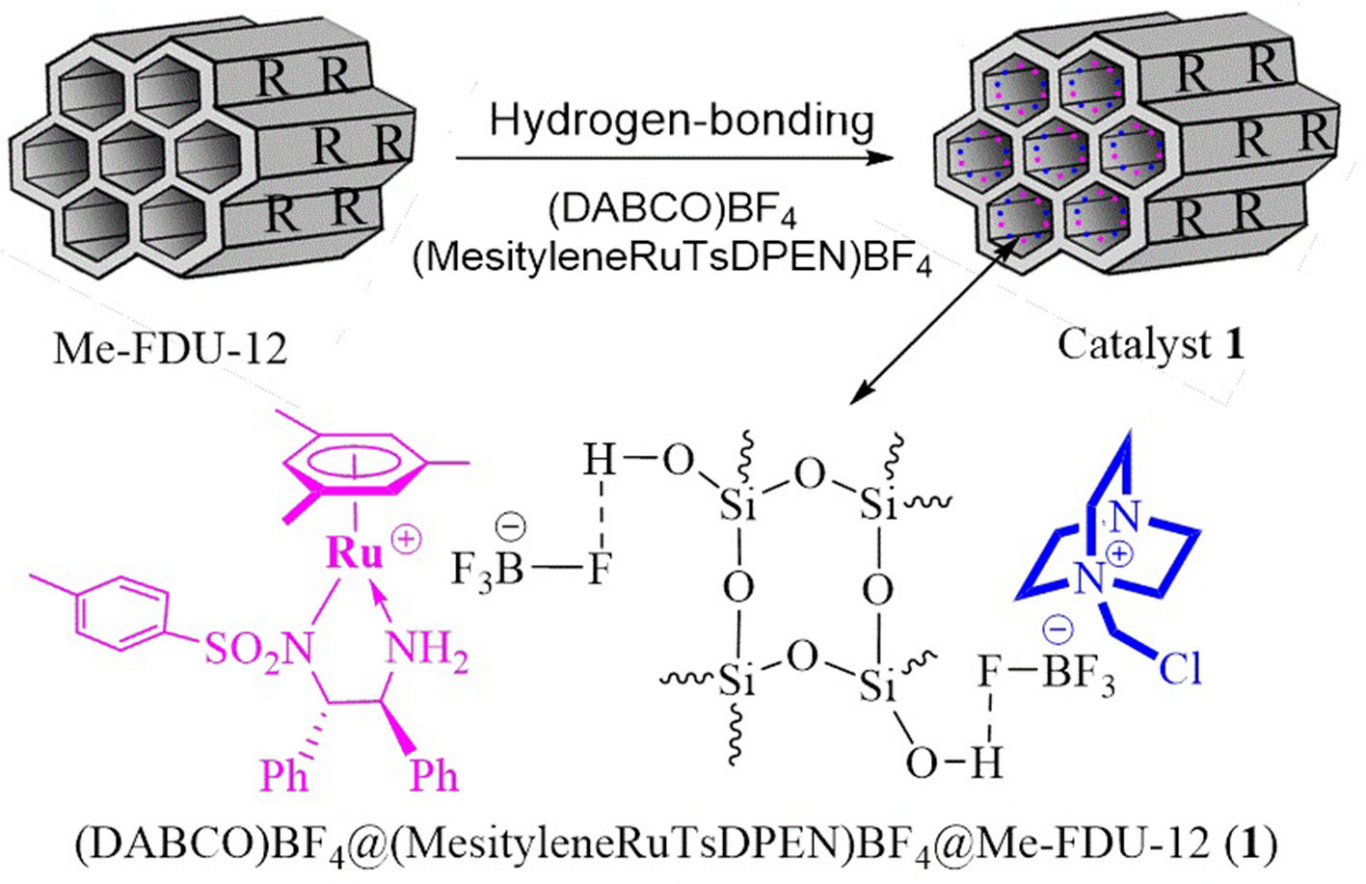
Preparation of heterogeneous catalyst 1.

The ${\text{BF}}_{4}^{-}$ anion–bonding of DABCO molecules as well as chiral ruthenium/diamine complexes immobilized in the nanopores of trimethysilylated FDU−12 (Me–FDU−12), in an abbreviated form as (DABCO)BF_4_@(MesityleneRuTsDPEN)BF_4_@Me–FDU−12 (**1**), ((DABCO)BF_4_ = 1–(chloromethyl)−1,4–diazabicyclo[2.2.2] octanium tetrafluoroborate) (Li and Liu, 2004; Li et al., 2005; Chauhan et al., 2014; Wen et al., 2014; Ying et al., 2014), (MesityleneRuTsDPEN)BF_4_ (Hashiguchi et al., 1995; Ohkuma et al., 2007): where TsDPEN = *N*–((*S*,*S*)−2–amino−1,2–diphenylethyl)−4–methylbenzenesulfonamide), was performed as shown in Figure 1. The mesoporous Me–FDU−12 was firstly obtained according to the reported approach with slightly modification (Fan et al., 2005; Ma et al., 2010), where the outer facial silanols of FDU−12 were protected through the treatment with ((CH_3_)_3_Si)_2_N compounds. Continuous hydrogen–bonding of (DABCO)BF_4_ and (MesityleneRuTsDPEN)BF_4_ to the inner silanols of nanopores in Me–FUD−12 then led to the crude catalyst. Finally, the well-defined catalyst **1** was obtained through a strict Soxhlet extraction from its corresponding crude form (see Figures S1, S2 of ESI).

**Figure 2:**
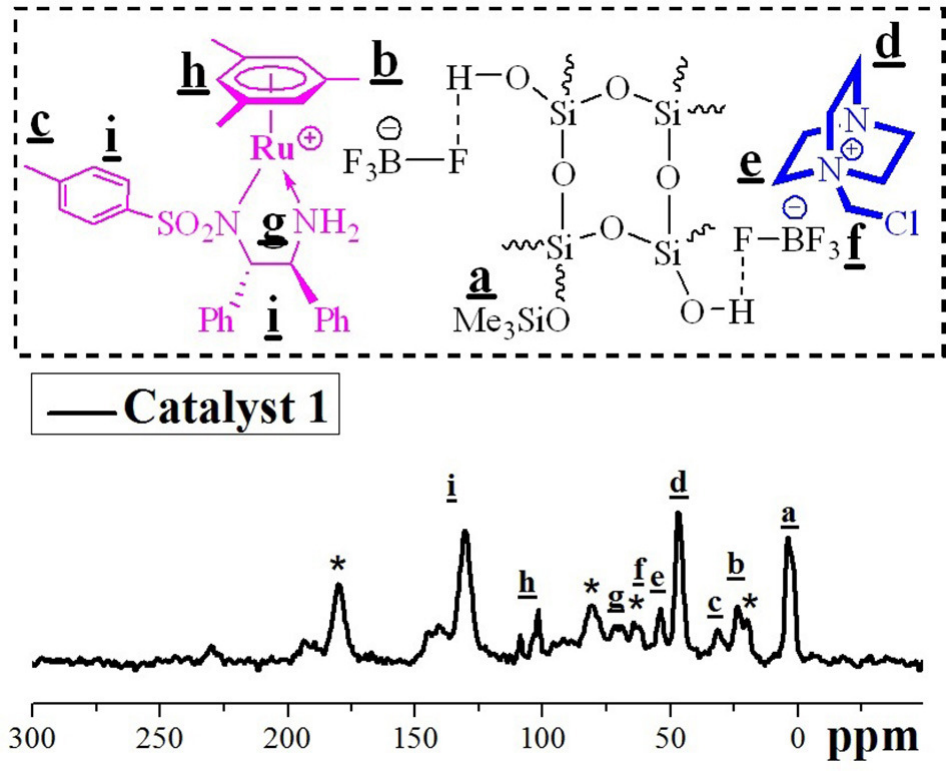
The solid–state ^13^C CP/MAS NMR spectrum of catalyst **1**.

Figure 2, the solid–state ^13^C cross–polarization (CP)/magic angle spinning (MAS) NMR spectroscopy of as-synthesized catalyst **1**, provided the direct evidence to the successful anchor of both anion functionalized chiral ruthenium/diamine complexes and DABCO–molecules species within the internal silanols of mesopores in Me–FDU−12 since both characteristic carbon signals could be observed clearly. In the part of DABCO–molecules, the characteristic peaks at 53.2 and 46.3 *ppm* were corresponded to the cyclic carbon atoms in DABCO moiety, which were similar to those attained with its homogeneous counterpart (Chauhan et al., 2014), suggesting the DABCO–functionality have been successfully incoporated in catalyst **1**. In the part of chiral ruthenium/diamine–complexes, besides the general carbon peaks around 70 ppm for the carbon atoms of –NCH groups and around 130 *ppm* for the carbon atoms of aromatic groups from TsDPEN, the characteristic peaks found at 108.4 and 101.5 *ppm* could be attributed to the carbon of mesitylene, while that at 23.3 *ppm* belonged to the carbon atoms of the *C*H_3_ groups of mesitylene. These carbons signals are the same as those of its homogeneous (MesityleneRuTsDPEN)BF_4_ (Ohkuma et al., 2007), demonstrating its well–defined single–site ruthenium center in catalyst **1**. Furthermore, to confirm DABCO molecules and chiral ruthenium/diamine–complexes dual active centers immobilized *via* the ${\text{BF}}_{4}^{-}$ anions, the solid–state ^19^F MAS NMR spectrum of catalyst **1** was used to compare with those of two corresponding single immobilized DABCO molecules and chiral ruthenium/diamine–complexes on Me-FDU-12. It was found that the wide F signals between −147 and −151 ppm are assigned to F signals in ${\text{BF}}_{4}^{-}$ anions interacting with dual centers *via* the ${\text{BF}}_{4}^{-}$ hydrogen bonding since they are similar to those of the their corresponding single immobilied DABCO molecules or chiral ruthenium/diamine–complexes solid-state ^19^F NMR spectra (see Figure S3 of ESI for their solid–state and liquid–state ^19^F NMR spectra). The other signals (at −181.3, −155.1, −101.6, and −74.2 ppm) denoted by asterisks were responded to the general “bulk” ${\text{BF}}_{4}^{-}$species that often appeared in ^19^F MAS NMR spectroscopy.

**Figure 3:**
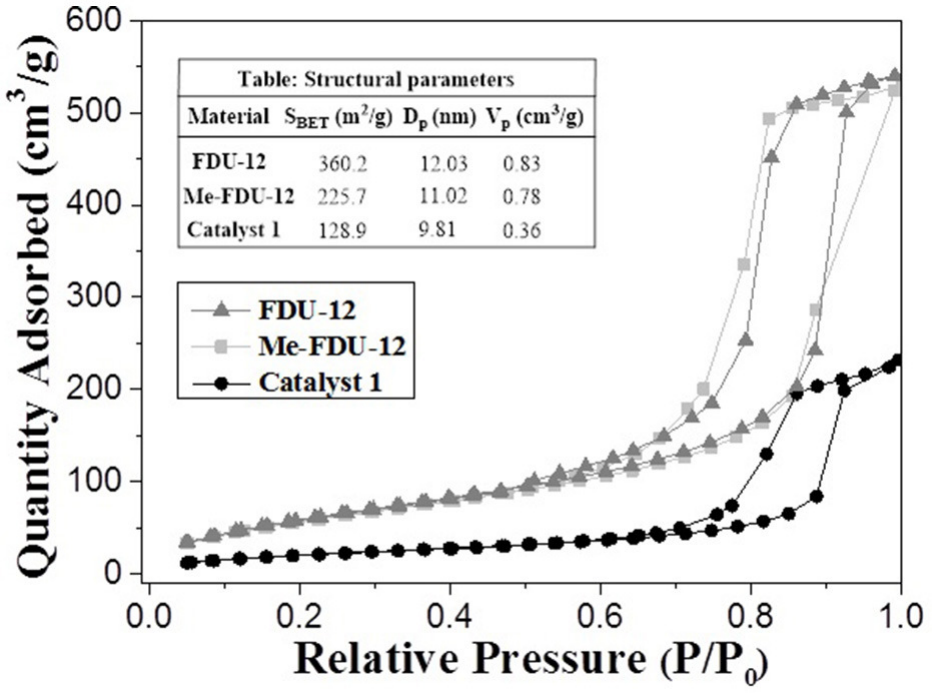
Nitrogen adsorption–desorption isotherms of Me–FDU−12 and catalyst **1**.

**Figure 4:**
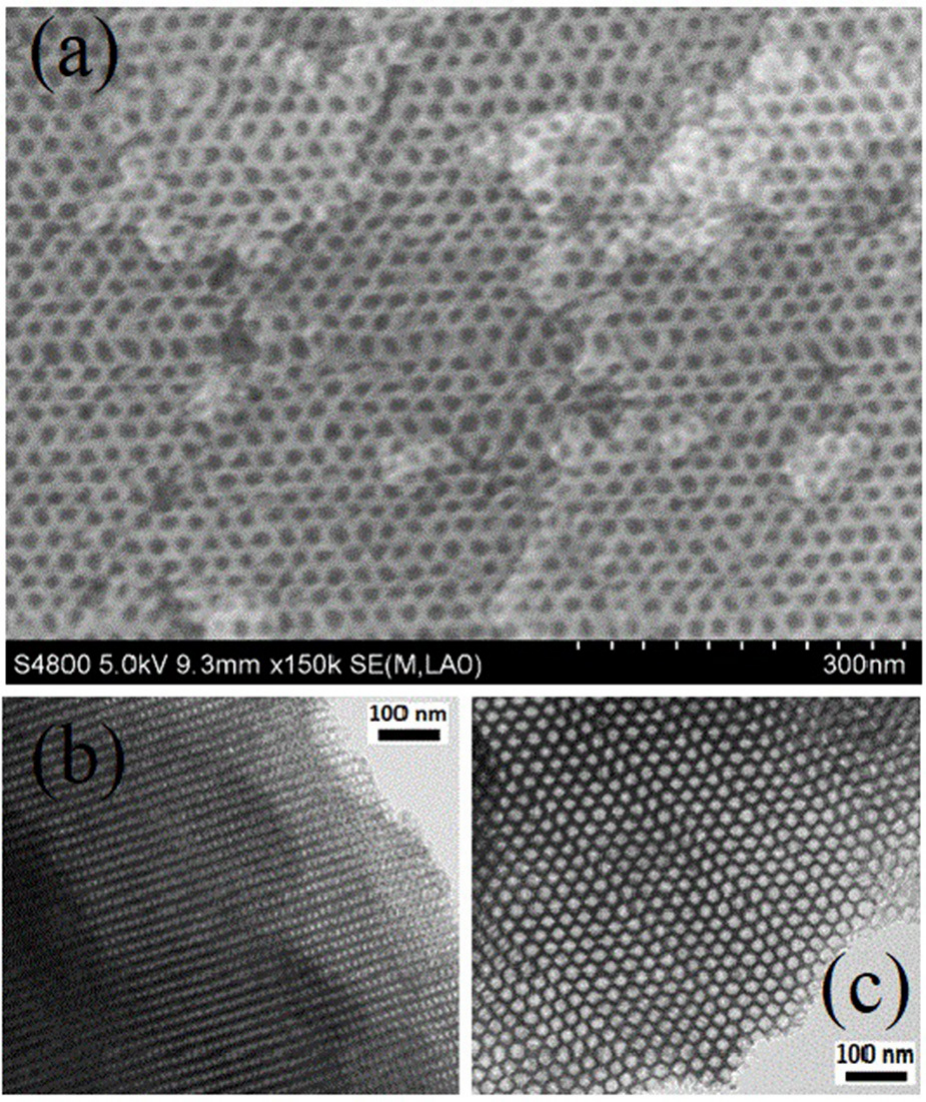
SEM images of catalyst **3, (b,c)** TEM images of catalyst **1** viewed along the **(a)** [100] and **(b)** [110] directions.

Figures 3, 4 revealed the mesostructure and morphology of catalyst **1**, which were characterized by scanning electron microscopy (SEM), nitrogen adsorption–desorption technique, transmission electron microscopy (TEM) with chemical mapping technique. The nitrogen adsorption–desorption isotherms of FDU−12, Me–FDU−12 and catalyst **1** presented the typical IV–type isotherms with H_2_ hysteresis loop (Figure 3), which are similar to those reported in the literatures, confirming its mesoporous structure(Fan et al., 2005; Ma et al., 2010). The SEM image of catalyst **1** disclosed the hexagonal arrays of uniform cages (Figure 4a) whereas the TEM images of catalyst **1** (Figures 4b,c) demonstrated the FDU−12–type face–centered–cubic mesostructures. Both confirmed that catalyst **1** retained the general morphological structure of FDU−12 after the immobilization of dual active species. Of significantly clear TEM image coupled with a chemical mapping (see Figure S4 of ESI) well demonstrated the uniformly distribution of ruthenium active centers within the inorganic silicate network of catalyst **1**.

All these characterizations and analyses elucidated that the obtained heterogeneous catalyst **1** had an operationally simple prepared procedure, well-defined dual active species as well as uniformly distributed ruthenium active centers, which would have a positive impact on its catalytic activity discussed below.

### Catalytic performance of catalyst 1

Cationic chiral *N*–sulfonylated diamine functionalized organometallic complexes, a type of catalytically efficient active species for asymmetric transfer hydrogenation (ATH) reaction, have obvious superiority in catalytic process with respect to their corresponding neutral ones since cationic complexes as real active species based on mechanism study could significantly favor asymmetric transfer hydrogenation process of ketones especially in an aqueous medium (Noyori and Hashiguchi, 1997; Liu et al., 2004a,b; Gladiali and Alberico, 2006; Wu et al., 2006, 2008; Ikariya and Blacker, 2007; Ohkuma et al., 2007; Wang et al., 2008, 2011, 2013b; Ding et al., 2013; Yang et al., 2014, 2016; Zhang et al., 2015). Therefore, direct immobilization of cationic ruthenium active species within the FDU−12 is beneficial to enhance ATH transformation. In particular, utilization of Me–FDU−12 as a support could ensure the dual active centers into the inner nanopores due the control of the protection step before the removal of surfactants during the synthetic procedure in the experiment part, where the potentially adjacent position of DABCO–molecule and chiral ruthenium/diamine–complex could promote cooperatively its catalytic performance.

**Figure 5:**
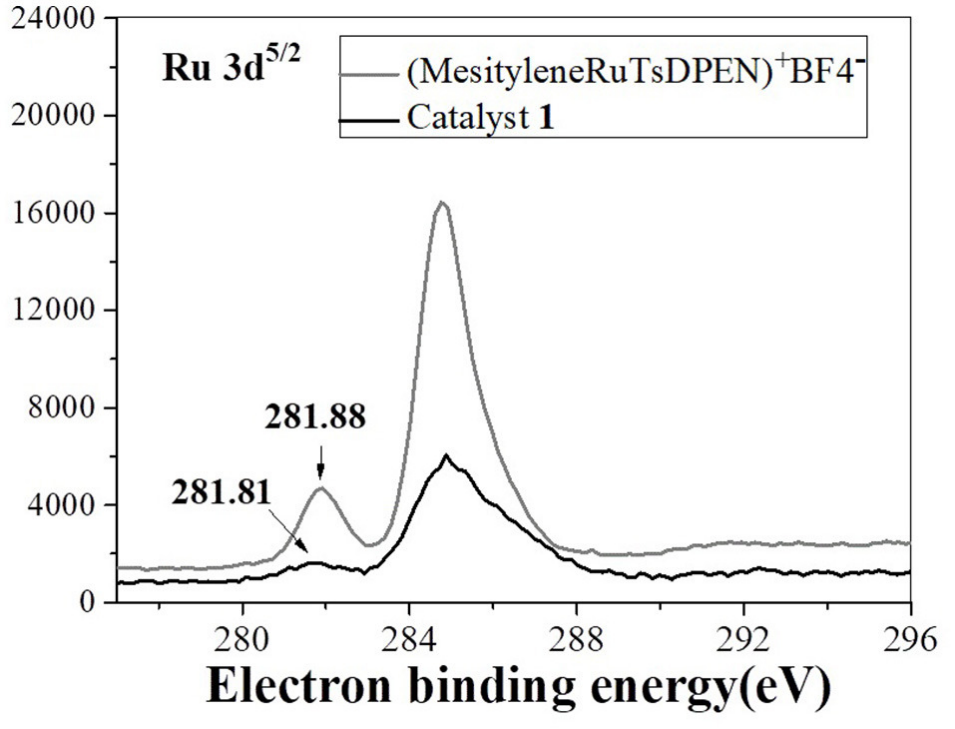
The XPS spectra of the homogeneous (MesityleneRuTsDPEN)BF_4_ and catalyst **1**.

With the well-established bifunctional catalyst **1** in hand, we transferred our attention to its catalytic and enantioselective performance in intermolecular aza–Michael addition/ATH one–pot process through the use of tandem reaction of acrylophenone and phenylamine as an example at the beginning. Based on the preliminary optimization of reaction conditions (see Table S1 of ESI), the tandem reaction was carried out with the 2.0 mol% of ruthenium–loading in **1** as a catalyst, the HCOONa as a hydrogen resource in the mixed solvents (H_2_O/^i^PrOH, v/v = 1/1) at 40°C reaction temperature. It was found that the organic transformation of 1–phenylprop−2–enone and aniline could provide the target product **3a** in 92% yield with 96% *ee* in 8 h. Such a yield was markedly higher than that of 79% yield obtained by the use of mixed homogeneous (DABCO)BF_4_ and homogeneous (MesityleneRuTsDPEN)BF_4_ as dual catalysts, even in a prolonged reaction time (Table 1, entry 1 and 2). This comparative analysis well demonstrated the existence of cross–interaction between (DABCO)BF_4_ and chiral (MesityleneRuTsDPEN)BF_4_ in the homogeneous catalytic conditions. Further products analysis found that about 19% of intermediate 1–phenyl−3–(phenylamino)propanone could not be converted, suggesting the DABCO part interfered in the reduction of (MesityleneRuTsDPEN)BF_4_. In other words, high catalytic activity of catalyst **1** demonstrated that the dual active species in catalyst **1** could overcome efficiently this kind of cross–interaction because the co-existent dual active had a potential site–isolated effect in the nanopores of catalyst **1**. Meanwhile, the obtained 96% *ee* in this tandem reaction was comparable to that of 97% *ee* obtained in single–step ATH transformation of 1–phenyl−3–(phenylamino)propanone, disclosing that the chiral catalytic environment of single–site (MesityleneRuTsDPEN)BF_4_ species in catalyst **1** was not affected during the immobilization procedure. This judgment could be further confirmed by a comparison of the XPS spectra. It was found that catalyst **1** and its homogeneous counterpart ((MesityleneRuTsDPEN)BF_4_) had the similar Ru 3d^5/2^ electron binding energies (281.88 vs. 281.81 eV), as displayed in Figure 5. This finding confirmed the presence of the similar catalytic environment as both had the similar electronic environment of active ruthenium centers.

**Table 1:** The aza–Michael addition/ATH one–pot enantioselective tandem reactions to prepare chiral γ-secondary amino alcohols^a^.

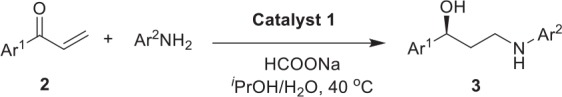				
**Entry**	**Ar**^1^**, Ar**^2^ **(3)**	**Time (*****h*****)**	**%Yield^b^**	**%*ee*^c^**
1	Ph, Ph (**3a**)	8	92	96
2	Ph, Ph (**3a**)	24	79	95^d^
3	Ph, 4–ClPh (**3b**)	10	89	95
4	Ph, 3–ClPh (**3c**)	10	81	94
5	Ph, 2–ClPh (**3d**)	10	92	97
6	Ph, 4–BrPh (**3e**)	10	91	96
7	Ph, 4–NO_2_Ph (**3f**)	10	83	98
8	Ph, 3–NO_2_Ph (**3g**)	10	85	96
9	Ph, 3,4–Me_2_Ph (**3h**)	12	76	96
10	Ph, 3,5–Me_2_Ph (**3i**)	12	73	95
11	Ph, 3–Cl−4–MePh (**3j**)	12	72	96
12	Ph, 3–MeOPh (**3k**)	12	81	96
13	4–FPh, Ph (**3l**)	10	92	92
14	4–ClPh, Ph (**3m**)	10	89	93
15	4–BrPh, Ph (**3n**)	10	91	94
16	4–IPh, Ph (**3o**)	10	88	94
17	4–MePh, Ph (**3p**)	12	82	94
18	4–MeOPh, Ph (**3q**)	12	80	95

a *Reaction conditions: catalyst **1** (19.61 mg, 2.0 μmol of Ru based on ICP–OES analysis), enones (0.10 mmol), amines (0.11 mmol), HCOONa (1.0 mmol), 2.0 mL of ^i^PrOH/H_2_O (v/v = 1: 1), and reaction time (8–24 h)*.

b *Isolated yields*.

c *The ee was determined chiral HPLC analysis (see SI in Figures S5, S7)*.

d *Data were obtained using the mixed DABCO–siloxane and its homogeneous MesityleneRuTsDPEN as dual catalysts*.

Promoted by the previous catalytic results, the as-synthesized heterogeneous catalyst **1** was thereafter examined using the aza–Michael addition/ATH enantioselective cascade reactions by systematically changing the substitution pattern of enones under the same reaction conditions, and the results were summarized in Table 1. It was found that all tandem reactions with the tested substrates could produce steadily the corresponding chiral products in satisfactory yields and excellent enantioselectivities. Gratifyingly, the electronic properties of substituents attached to the aromatic rings of Ar^2^ group affected slightly reactivity, where the reactions with electron–withdrawing substituents had inferior yields relative to those results obtained with electron–donating substituents (Entries 3–8, 9–12). However, their enantioselectivities were not affected significantly for all chiral products bearing electron–withdrawing and/or –donating substituents of Ar^2^ group had high *ee* values (Entries 3–12). Similarly, in the case of the substituents on the aromatic rings of Ar^1^ group, this phenomenon was also observed (Entries 13–18).

**Figure 6:**
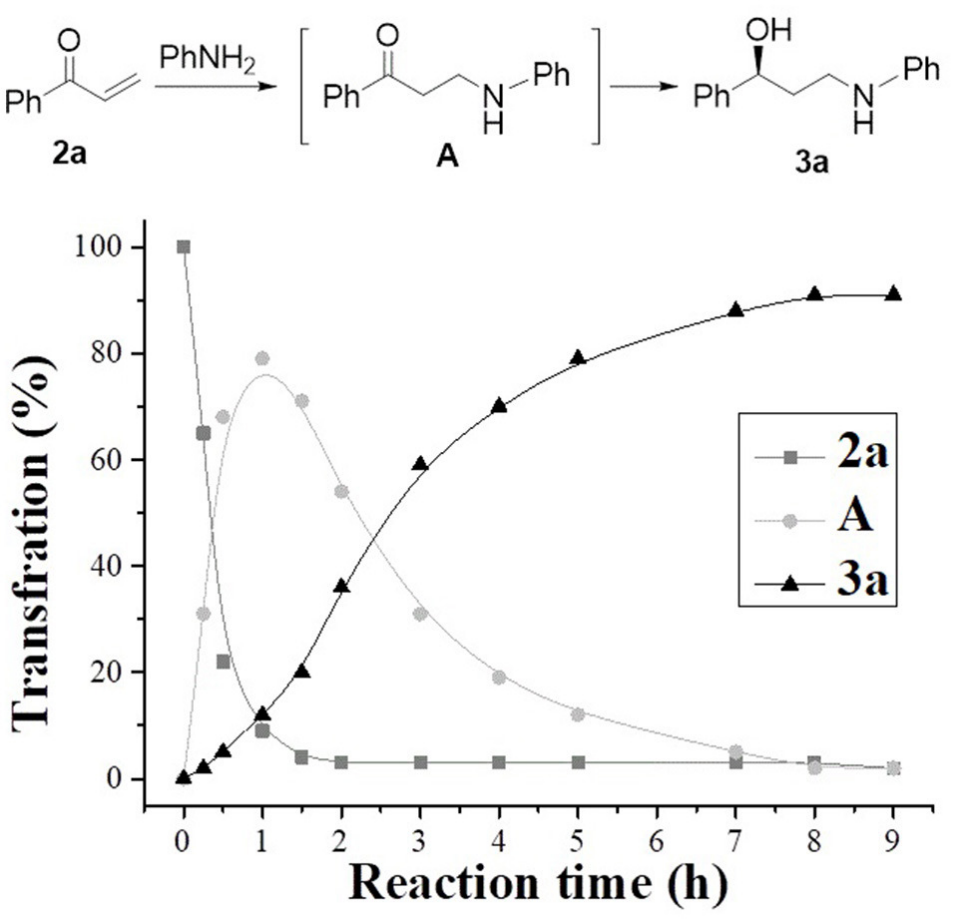
Time course of the aza–Michael addition/ATH of 1–phenylprop −2–enone and aniline to (*S*) −1–phenyl −3–(phenylamino) propanol (reaction was performed with 1 equivalent of 1–phenylprop−2–enone, 1.1 equivalent of aniline, 2.0 mol % of catalyst **1**, 10.0 equivalent of HCOONa at 40°C).

In order to confirm the tandem behavior and explore the catalytic nature of catalyst **1**, the time course for one–pot organic transformation of acrylophenone and phenylamine to chiral γ-secondary amino alcohols **3a** was investigated. It is worth noting that both the aza–Michael addition of aniline to acrylophenone (**2a**) and the ATH transformation of reaction intermediate **A** to chiral γ-secondary amino alcohols **3a** occur simultaneously during the first 1 h, where the content of 1–phenylprop−2–enone (**2a**) rapidly decreases down to 9% and that of (*S*)−1–phenyl−3–(phenylamino)propanol (**3a**) rises to 12%, as outlined in Figure 6. Meanwhile, during the first hour, the maximum 79% yield of the intermediate 1–phenyl−3–(phenylamino)propanone (**A**) is observed. Subsequently, one–pot organic transformation of acrylophenone and phenylamine proceeds smoothly, resulting in (*S*)−1–phenyl−3–(phenylamino)propanol (**3a**) in 92% yield within 8 h. Such a time course reveals the catalytic nature of this aza–Michael addition/ATH enantioselective undergoes a tandem process.

**Figure 7:**
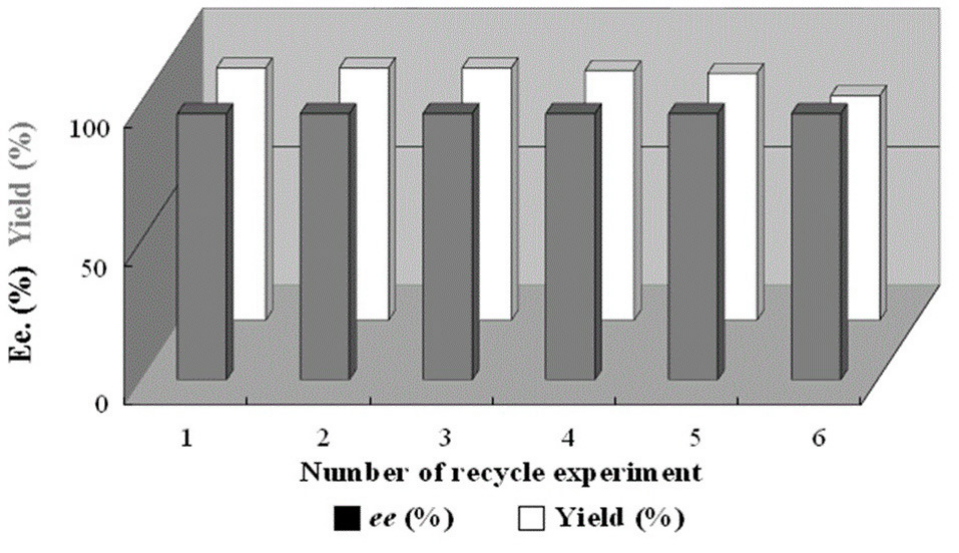
Reusability of catalyst **1** for the organic transformation of 1–phenylprop − 2–enone and aniline to (*S*) − 1–phenyl − 3–(phenylamino)propanol.

As a heterogeneous catalyst, it was highly expected that it could be recovered by simple centrifugation and the recycled catalyst could retained highly reactivity even after several recycle. Indeed, the newly developed catalyst **1** could be easily separated and recovered using centrifugation techniques from reaction solution, providing the desired adduct **3a** in 90% yield with 96% *ee* in fifth recycled experiment *via* the one-pot aza–Michael addition/ATH enantioselective tandem reaction of acrylophenone and aniline (Figure 7, also see Table S2 and Figure S7 of ESI). In order to explain the decreased reactivity after fifth recycle (at sixth run), an analysis of the Ru–leaching was carried out by the use of ICP–OES techniques. The result showed that the amount of Ru at fifth run was 9.59 mg (0.0941 mmol) per gram of **1**, meaning that the leaching amount of Ru should be 7.8% with respect to the original value (10.40 mg, 0.1020 mmol of Ru per gram of catalyst). For comparison, ICP–OES analysis after fifth recycle (at sixth run) indicated that the amount of Ru was 9.21 mg (0.0904 mmol) per gram of **1** and 11.6% of Ru was lost, revealing the obviously decreased reactivity at sixth run was attributed to the large number of Ru–leaching in catalyst **1**. In addition, the loss of (DABCO)BF_4_ molecules after fifth recycle was about 20% *via* a comparison of their element analyses.

## Conclusions

In conclusions, by using a ${\text{BF}}_{4}^{-}$ anion–bonding strategy, we develop a facile approach to one–steps assemble both DABCO–molecules and chiral ruthenium/diamine–complexes species into the inner surface of Me–FDU−12, constructing a bifunctional heterogeneous catalyst. As we envisaged, the DABCO–molecules in catalyst **1** as a Lewis base enables aza–Michael addition whereas chiral ruthenium/diamine–complex as chiral promoter catalyzes asymmetric transfer hydrogenation. Synergistic contribution of both functionality boost greatly one–pot enantioselective synthesis of a series of chiral γ-secondary amino alcohols from enones and amines in a tandem manner. This method described here offers an operationally simple synthetic procedure to fabricate bifunctional heterogeneous catalyst used for synthesis of valuable chiral pure γ-secondary amino alcohols.

## Author contributions

LL is mainly responsible for the preparation of heterogeneous catalyst, corresponding characterization and part of catalytic reactions. DY and ZZ carried out the catalytic reactions. YS finished the time course experiment, and LZ is responsible for the chiral HPLC analysis. RL and GL are responsible for the characterization analysis and writing.

### Conflict of interest statement

The authors declare that the research was conducted in the absence of any commercial or financial relationships that could be construed as a potential conflict of interest.
