# Supplementary material for: Boron Tetrafluoride Anion Bonding Dual Active Species Within a Large–Pore Mesoporous Silica for Two–Step Successive Organic Transformaion to Prepare Optically Pure Amino Alcohols
Source: Front Chem. 2018 Jul 6;6:272. doi: 10.3389/fchem.2018.00272 (PMC6043685; doi:10.3389/fchem.2018.00272)

Supplementary Material

Boron Tetrafluoride Anion Bonding Dual Active Species Within a Large–pore Mesoporous Silica for Two–step Successive Organic Transformaion to Prepare Optically Pure Amino Alcohols

Liang Li, Dongfeng Yang, Zhongrui Zhao, Yongkang Song, Lei Zhao, Rui Liu*, Guohua Liu*

Key Laboratory of Resource Chemistry of Ministry of Education, Shanghai Key Laboratory of Rare Earth Functional Materials, Shanghai Normal University, Shanghai, 200234, China.

CONTENTS

Experimental..2

Figure S1. FT-IR spectra of Me-FDU-12 and catalyst **1**. 3

Figure S2. Solid-state ^{29}Si CP MAS NMR spectra of Me-FDU-12 and catalyst **1**. 3

Figure S3. (a) Solid-state and (b) liquid-state ^{19}F CP MAS NMR spectra of (DABCO)BF_4@Me–FDU–12 and (MesityleneRuTsDPEN)BF_4@Me–FDU–12..........4

Figure S4. TEM image with a chemical mapping of **1** showing the distribution of Si (white) and Ru (red). 7

Figure S5. The TG/DTA curves of Me-FDU-12, (DABCO)BF_4@FDU-12 (1′) and catalyst **1**. 8

Table S1. Optimization of co-solvents for the the aza-Michael addition/ATH one–pot Enantioselective tandem reaction of 1–phenylprop–2–enone and aniline to (*S*)-1-Phenyl-3-(phenylamino)propanol....................................11

Figure S6. HPLC analyses for chiral products. 12

Table S2. Reusability of catalyst **1** for the aza–Michael addition/ATH one–pot Enantioselective tandem reaction of 1–phenylprop–2–enone and aniline....................29

Figure S7. Reusability of catalyst **1** for the aza–Michael addition/ATH one–pot Enantioselective tandem reaction of 1–phenylprop–2–enone and aniline....................29

Figure S8. Characterizations of chiral products. 32

Experimental

General. All experiments, which are sensitive to moisture or air, were carried out under an Ar atmosphere using the standard Schlenk techniques. Mesoporous silica was synthesized by using block copolymer and organic solvent as the templates with additive of inorganic salts. 1,3,5-trimethylbenzene (TMB), tetraethoxysilane (TEOS), KCl, Hexamethyldisilazane, Triethylenediamine, Dichloromethane, 4-(methylphenylsulfonyl)-1,2-diphenylethylenediamine [(*S*,*S*)-TsDPEN], surfactant triblock copolymers $EO_{106}PO_{70}EO_{106}$ (Pluronic F127), $[mesityleneRuCl_2]_2$ were purchased from Sigma-Aldrich Company Ltd and used as received. Compound of 1-(chloromethyl)-1,4-diazabicyclo[2.2.2]octanium tetrafluoroborate [*Catal. Sci. Technol.*, 2014, **4**, 3945-3952] and $[mesityleneRuClBF_4]_2$ [*J. Am. Chem. Soc.* 2015, 137, 7083−7086] were synthesized according to the reported literature.

Characterization. Ru loading amounts in this catalyst was analyzed using an inductively coupled plasma optical emission spectrometer (ICP–OES, Varian VISTA−MPX). Fourier transform infrared (FT−IR) spectra were collected on a Nicolet Magna 550 spectrometer using a KBr method. Scanning electron microscopy (SEM) image was obtained using a JEOL JSM−6380LV microscope operating at 20 kV. Transmission electron microscopy (TEM) image was performed on a JEOL JEM2010 electron microscope at an acceleration voltage of 220 kV. X−ray photoelectron spectroscopy (XPS) measurement was performed on a Perkin−Elmer PHI 5000C ESCA system. A 200 μm diameter spot size was scanned using a monochromatized Aluminum Kα X−ray source (1486.6 eV) at 40 W and 15 kV with 58.7 eV pass energies. All the binding energies were calibrated by using the contaminant carbon (C1s = 284.6 eV) as a reference. Nitrogen adsorption isotherms were measured at 77 K with a Quantachrome Nova 4000 analyzer. The samples were measured after being outgassed at 423 K overnight. Pore size distributions were calculated by using the BJH model. The specific surface areas (SBET) of samples were determined from the linear parts of BET plots (p/p0 = 0.05−1.00). Elemental analysis was performed with a Carlo Erba 1106 Elemental Analyzer. Solid−state NMR experiments were explored on a Bruker AVANCE spectrometer at a magnetic field strength of 9.4 T with 1H frequency of 400.1 MHz, 13C frequency of 100.5 MHz, 29Si frequency of 79.4 MHz and 19F frequency of 169.3 MHz with 4 mm rotor at two spinning frequency of 5.5 kHz and 8.0 kHz, TPPM decoupling is applied in the during acquisition period. ^{1}H cross polarization in all solid−state NMR experiments were employed using a contact time of 2 ms and the pulse lengths of 4μs.

Figure S1. FT-IR spectra of Me-FDU-12 and catalyst **1.**

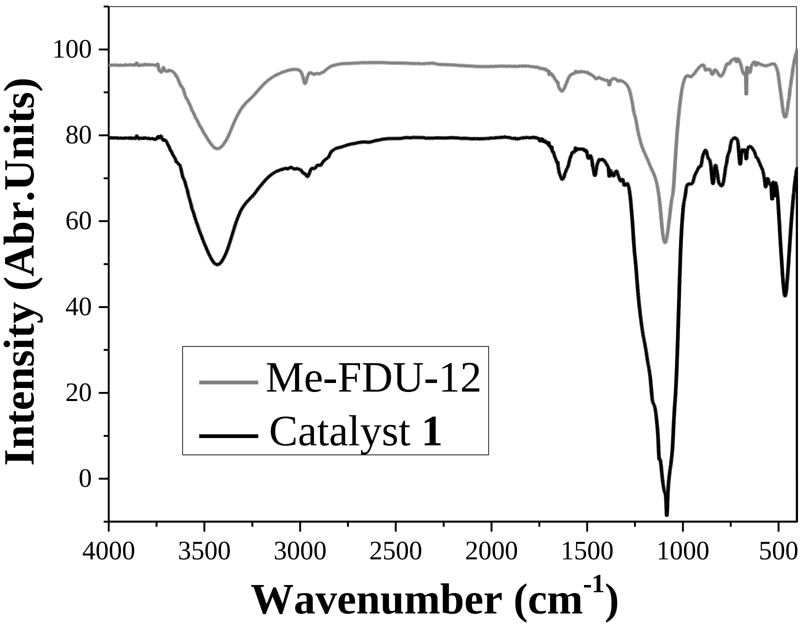

Figure S2. Solid-state ^{29}Si CP MAS NMR spectra of Me-FDU-12 and catalyst **1**.

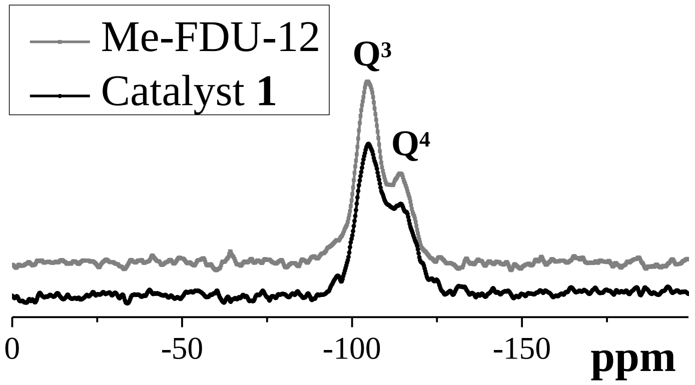

Figure S3. Solid-state ^{19}F CP MAS NMR spectra of catalyst **1** (a), BF_4@Me–FDU–12 (b), (DABCO)BF_4@Me–FDU–12 (c), and (MesityleneRuTsDPEN)BF_4@Me–FDU–12 (d).

(a) The solid-state ^{19}F CP MAS NMR spectrum of catalyst **1**

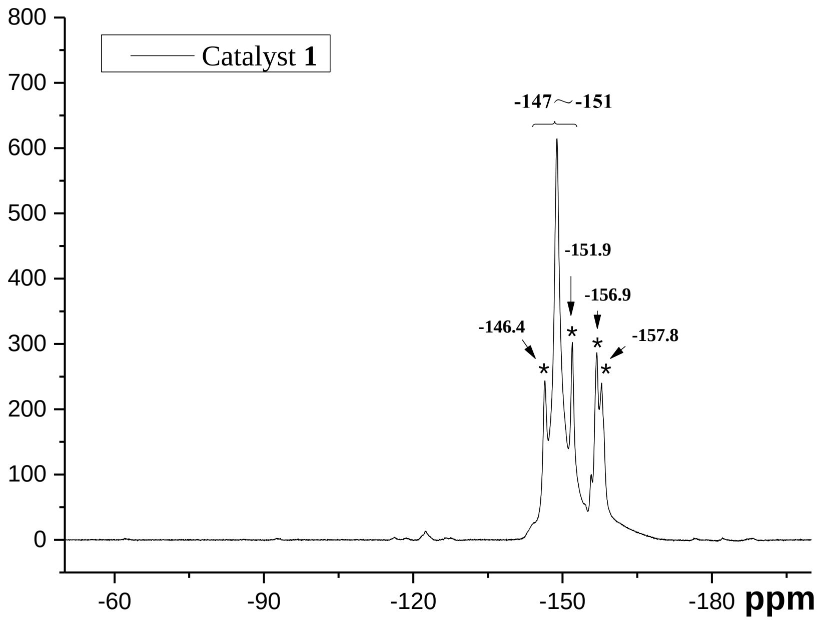

(b) The solid-state ^{19}F CP MAS NMR spectrum of **BF_4@Me-FDU-12**

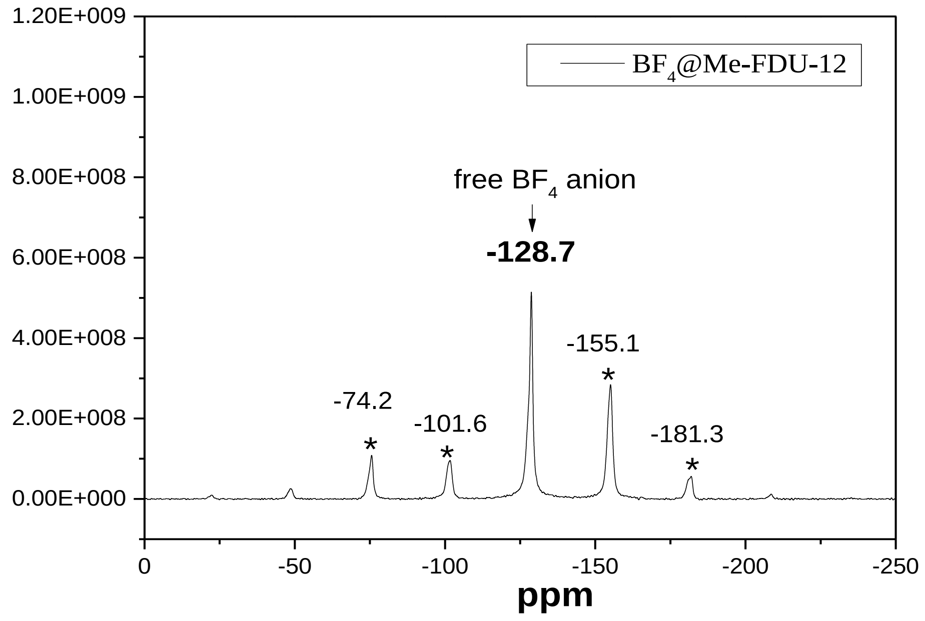

(c) The solid-state ^{19}F CP MAS NMR spectrum of (DABCO)BF_4@Me-FDU-12

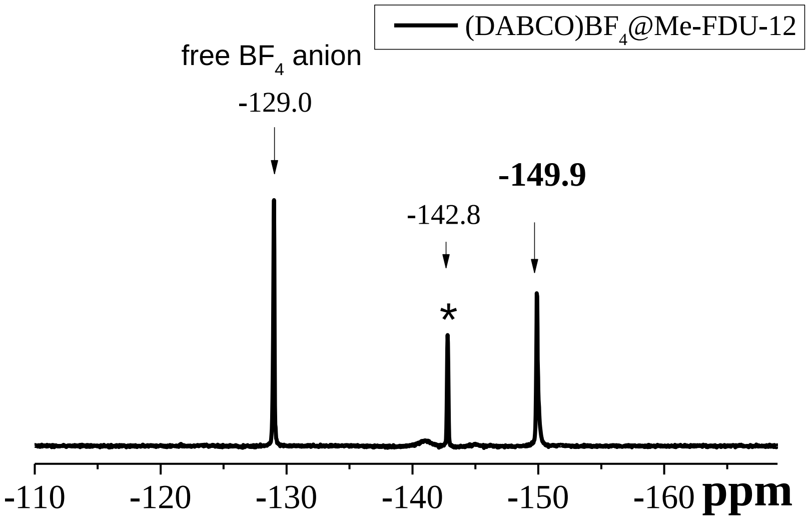

(c') The liquid-state ^{19}F NMR spectrum of (DABCO)BF_4

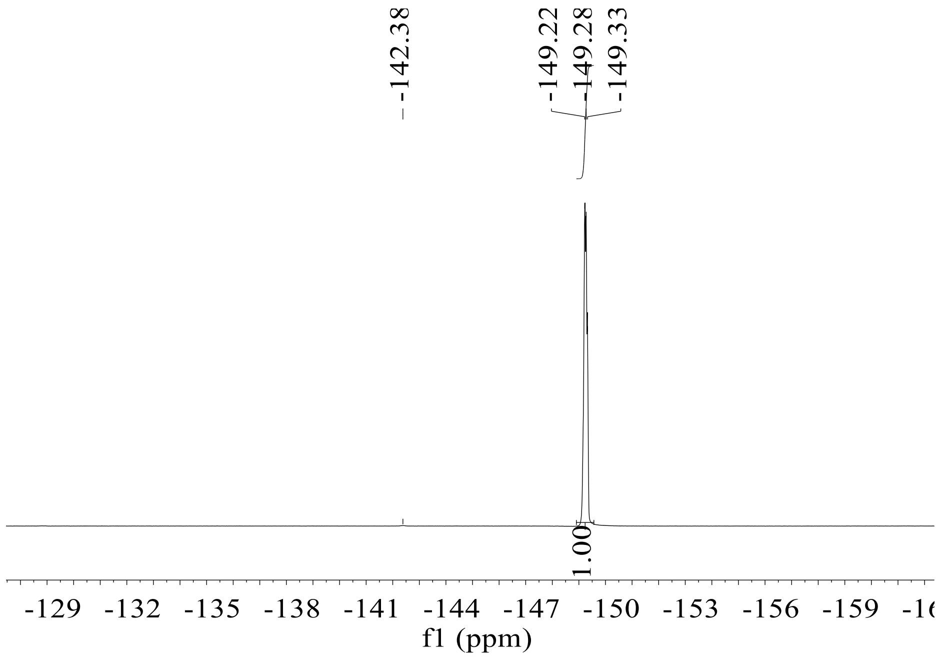

(d) The solid-state ^{19}F CP MAS NMR spectrum of (MesityleneRuTsDPEN)BF_4@Me-FDU-12.

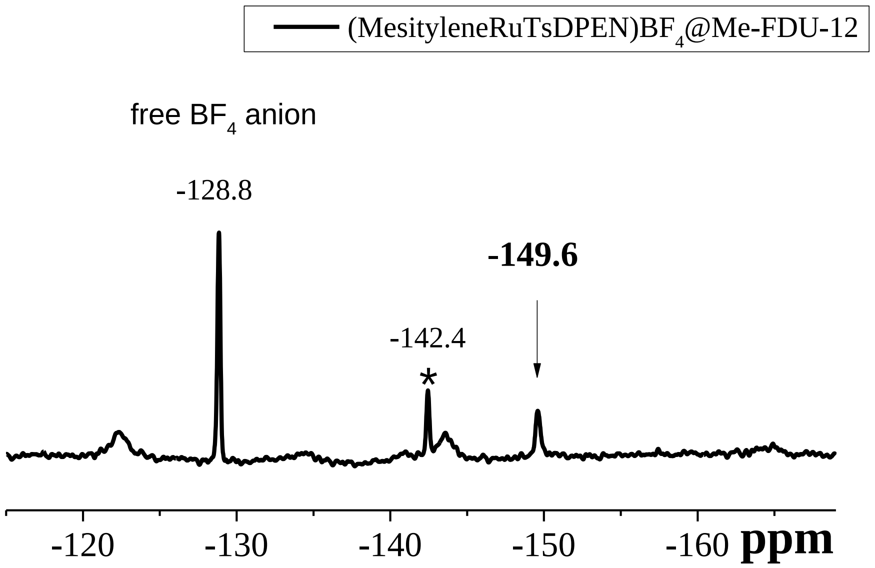

(d') The liquid-state ^{19}F NMR spectrum of (MesityleneRuTsDPEN)BF_4

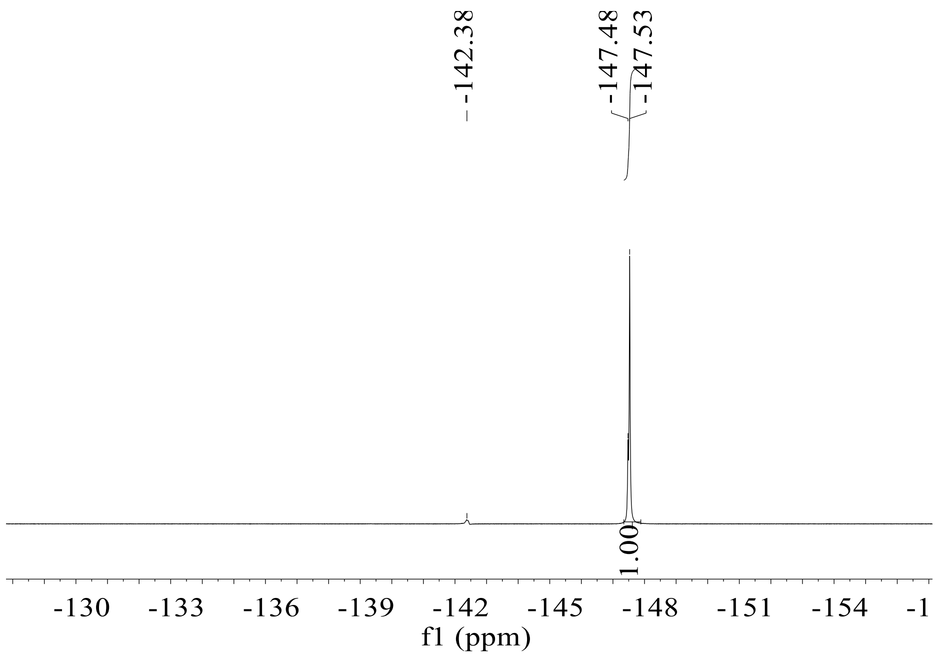

Figure S4. TEM image with a chemical mapping of **1** showing the distribution of Si (white) and Ru (red).

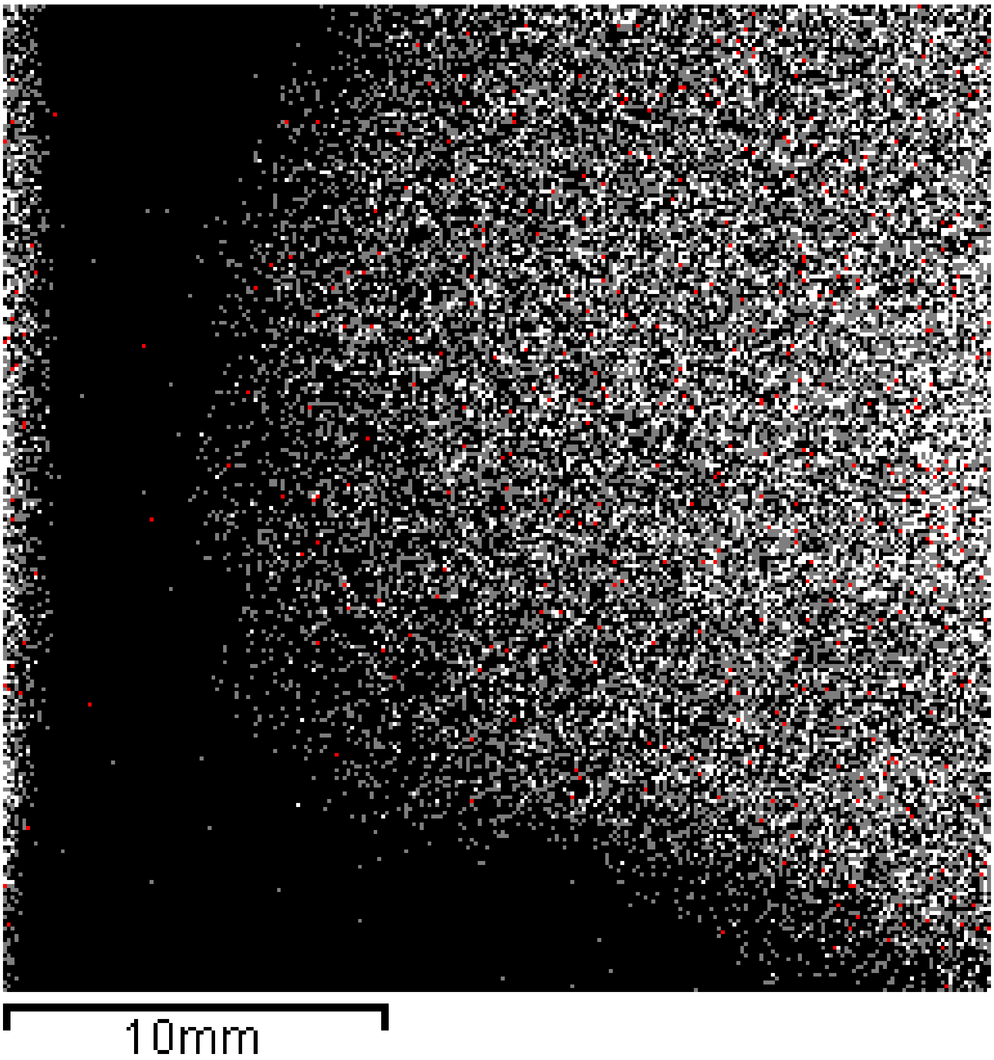

Figure S5. The TG/DTA curves of Me-FDU-12, (DABCO)BF_4@FDU-12 (**1′**) and catalyst **1.**

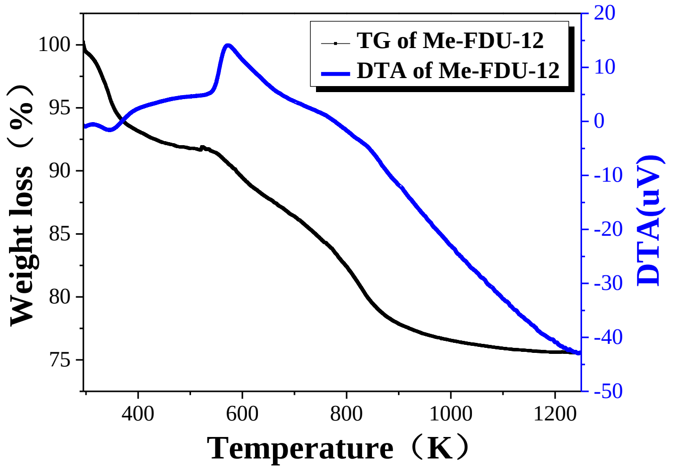

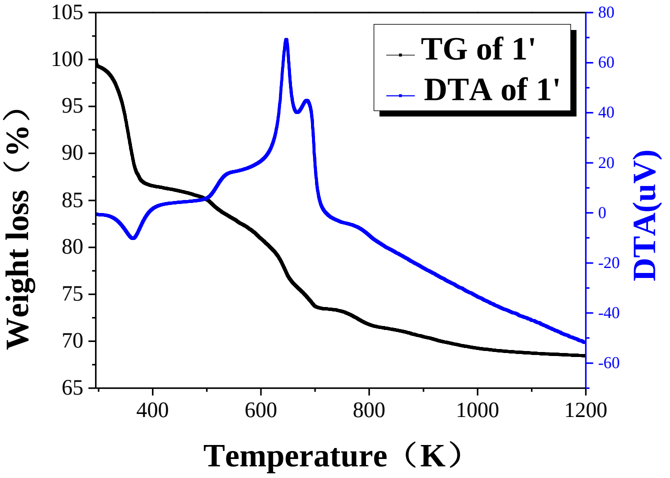

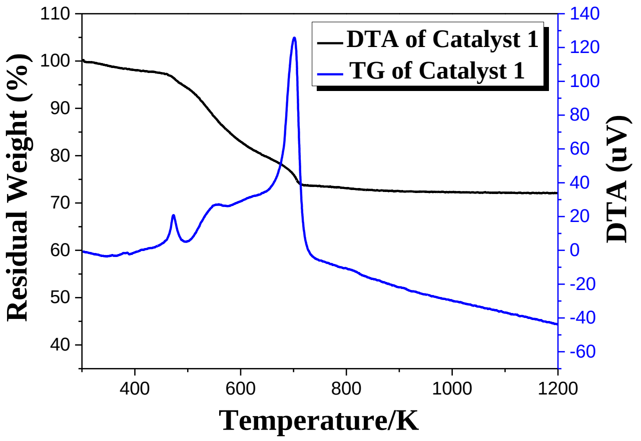

For Me-FUD-12, it was found easily that a similar endothermic peak around 345 K with weight loss of (100-92.28) 7.72% were strongly similar to that of parent Me-FUD-12 due to the release of physical adsorption water. It was worth mentioning that the all exothermic peaks were combined into one complicated exothermic peak between 440 K and 1200 K with weight loss of (92.28-75.60) 16.68% could be assigned to the oxidation of organic molecules (including alkyl fragments and part of the residual surfactant). Because the totally weight loss of organic moieties was 16.68% per 92.28% the extracted catalyst when eliminated the part of water, meaning the whole weight loss 18.07% of the oxidation of the organic molecules per 100% materials.

For the (DABCO)BF_4@FDU-12 (**1′**), it was found easily that a similar endothermic peak around 363 K with weight loss of (100-86.19) 13.71% were strongly similar to that of parent Me-FUD-12 due to the release of physical adsorption water. It was worth mentioning that the all exothermic peaks were combined into one complicated exothermic peak between 440 K and 1200 K with weight loss of (86.19-68.47) 17.71% could be assigned to the oxidation of organic molecules (including hydrogen-bonding organic molecules, part of the residual surfactant). Because the totally weight loss of organic moieties was 17.71% per 86.19% the extracted catalyst when eliminated the part of water, meaning the whole weight loss 20.56% of the oxidation of the organic molecules per 100% materials.

As compared the weight loss of the Me-FUD-12 with (DABCO)BF_4@FDU-12, the true weight loss of (DABCO)BF_4 moieties (Mr = 249.09) was 2.49% (20.56-18.07) per 100% materials. This finding means that the mole amounts of (DABCO)BF_4 is 0.010 mol%, demonstrating the 10% of the DABCO loading per gram of (DABCO)BF_4@FDU-12 (**1′**) material.

For the catalyst 1, it was found easily that a similar endothermic peak around 347 K with weight loss of (100-97.55) 2.45% were strongly similar to that of parent Me-FUD-12 due to the release of physical adsorption water. It was worth mentioning that the all exothermic peaks were combined into one complicated exothermic peak between 440 K and 1200 K with weight loss of (97.55-72.11) 25.44% could be assigned to the oxidation of organic molecules (including hydrogen-bonding organic molecules and complexes, part of the residual surfactant). Because the totally weight loss of organic moieties was 25.44% per 97.03% the extracted catalyst when eliminated the part of water, meaning the whole weight loss 26.21% of the oxidation of the organic molecules per 100% materials.

As compared the weight loss of the $DABCOBF_4$@Me-FDU-12 (**1′**) with catalyst **1**, the true weight loss of $(MesityleneTsDPEN)BF_4$ moieties (Mr = 570.2) was 5.65% (26.21-20.56) per 100% materials. This finding means that the mole amounts of $(MesityleneRuTsDPEN)BF_4$ is 0.009924 mol%, demonstrating the 10.112 mg (0.09924 mmol of Ru) of the Ru loading per gram of catalyst **1**. This result is nearly same as that obtained with ICP analysis (the Ru loadings was 10.40 mg (0.1020 mmol of Ru) per gram of catalyst).

Table S1. Optimization of dual species-loadings and solvents for the aza-Michael addition/ATH one–pot Enantioselective tandem reaction of 1–phenylprop–2–enone and aniline to (*S*)-1-Phenyl-3-(phenylamino)propanol.[a]

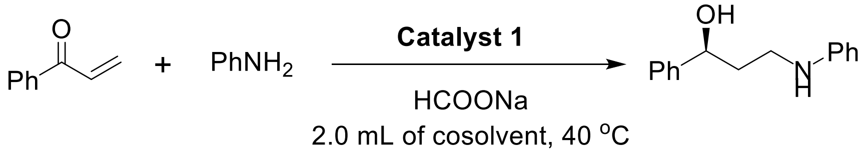

Entry	Catalyst/base-loading (%/%)	Solvent (v/v)	time (h)	Yield (%)[b]	*ee* (%)[c]
1	2/10	H_2O	24	68	78
2	2/10	EtOH/H_2O (1:1)	8	86	95
3	2/10	MeOH/H_2O (1:1)	8	77	94
4	2/10	^{i}PrOH/H_2O (1:1)	8	92	96
5	2/10	^{i}PrOH/H_2O (2:1)	8	95	93
6	2/10	^{i}PrOH/H_2O (1:2)	8	90	95
7	1.25/10	^{i}PrOH/H_2O (1:1)	8	89	96
8	2.25/10	^{i}PrOH/H_2O (1:1)	8	93	94

[a] Reactions were performed with catalyst **1**, 0.10 mmol of 1–phenylprop–2–enone, 0.11 mmol of aniline, and 1.0 mmol of HCOONa in 2.0 mL of co-solvent at 40 ℃. [b] Isolated yield. [c] Determined by HPLC on a Daciel Chiralcel OD-H column.

Figure S6. HPLC analyses for chiral products

<u>3a:</u> (*S*)-1-phenyl-3-(phenylamino)propan-1-ol (HPLC (OD-H, elute: Hexane/*i*-PrOH = 90/10, detector: 254 nm, flow rate: 1 mL/min, 25 °C), t_1 = 27.3 min (major), t_2 = 31.1 min.)

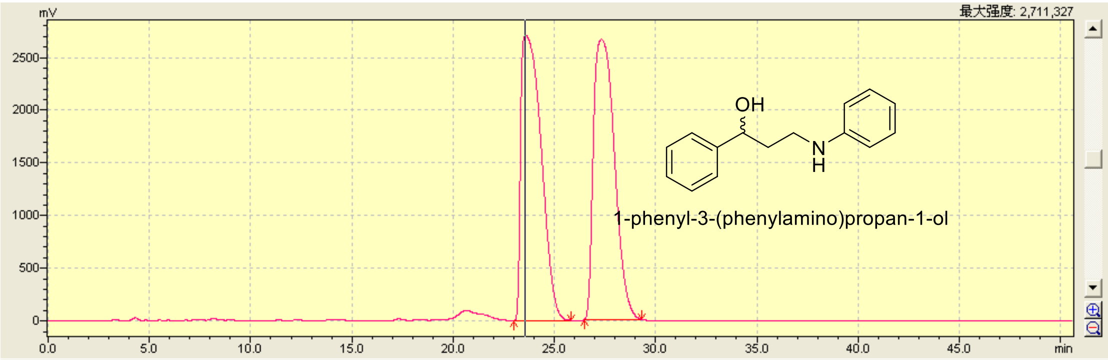

化合物表视图

ID#	名称	保留时间	峰#	面积	高度	面积%
1	RT23.563	23.563	1	189601304	2706599	49.6216
2	RT27.307	27.307	2	192492741	2661421	50.3784

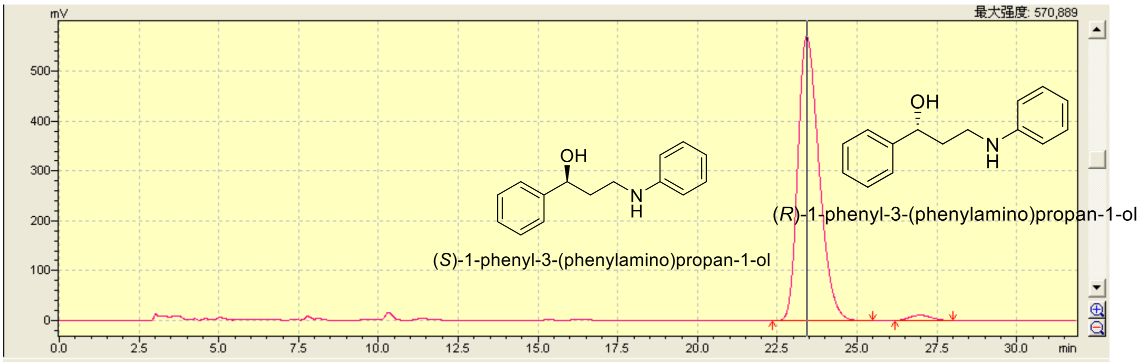

化合物表视图

ID#	名称	保留时间	峰#	面积	高度	面积%
1	RT23.417	23.417	1	26407936	571119	98.0602
2	RT26.964	26.964	2	522396	10752	1.9398

<u>3b:</u> (*S*)-3-((4-chlorophenyl)amino)-1-phenylpropan-1-ol (HPLC (OD-H, elute: Hexane/*i*-PrOH = 90/10, detector: 254 nm, flow rate: 1 mL/min, 25 °C), t_1 = 21 .5min (major), t_2 = 25.9 min.)

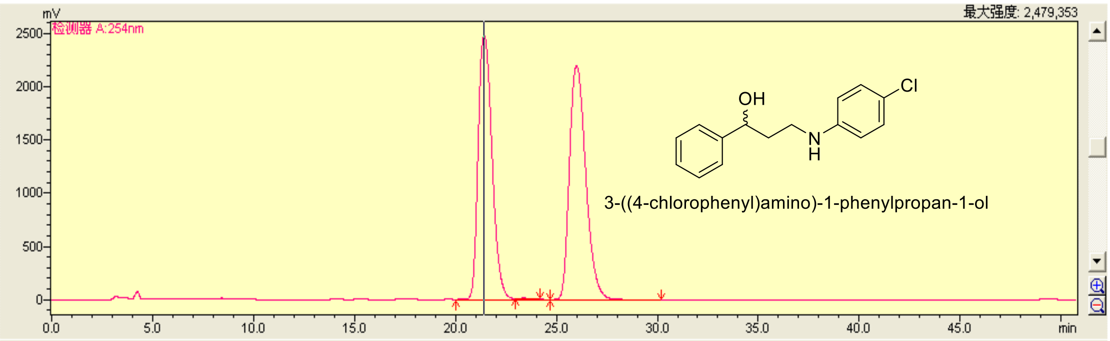

化合物表视图

ID#	名称	保留时间	峰#	面积	高度	面积%
1	RT21.415	21.415	1	118864195	2477582	48.9486
2	RT25.995	25.995	3	123734758	2199300	50.9543

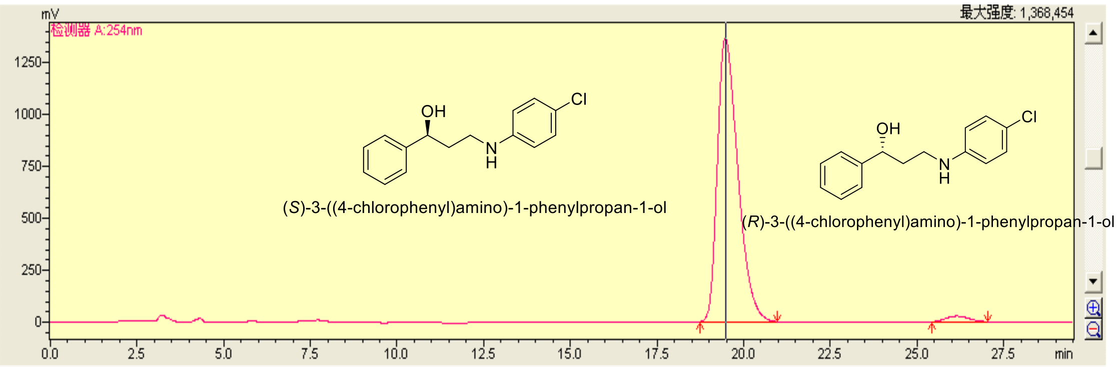

化合物表视图

ID#	名称	保留时间	峰#	面积	高度	面积%
1	RT19.469	19.469	1	56283386	1365797	97.7135
2	RT26.140	26.140	2	1317048	28080	2.2865

<u>3c:</u> (*S*)-3-((3-chlorophenyl)amino)-1-phenylpropan-1-ol (HPLC (OD-H, elute: Hexane/*i*-PrOH = 90/10, detector: 254 nm, flow rate: 1 mL/min, 25 ^{o}C), t_1 = 19.5 min (major), t_2 = 26.0 min.**)**

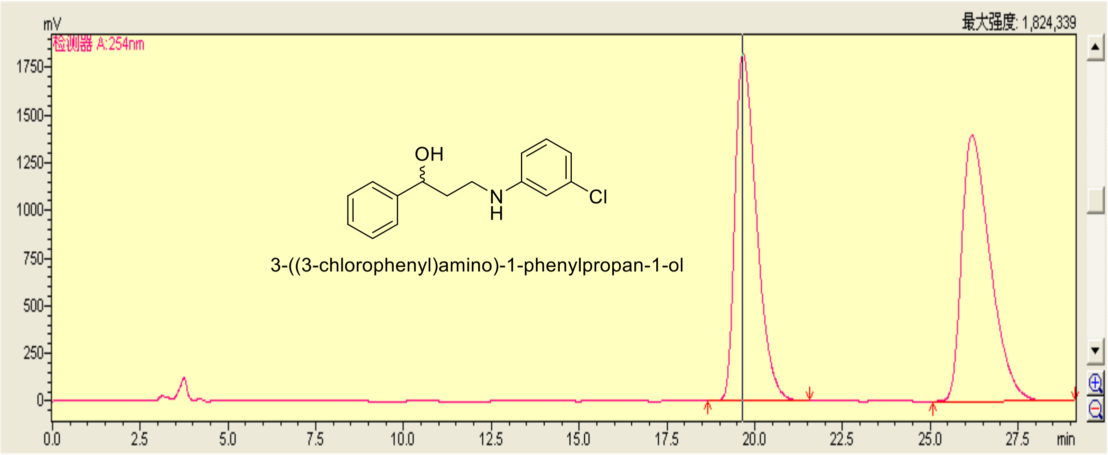

化合物表视图

ID#	名称	保留时间	峰#	面积	高度	面积%
1	RT19. 665	19. 665	1	77533321	1824010	49. 5001
2	RT26. 189	26. 189	2	79099454	1394221	50. 4999

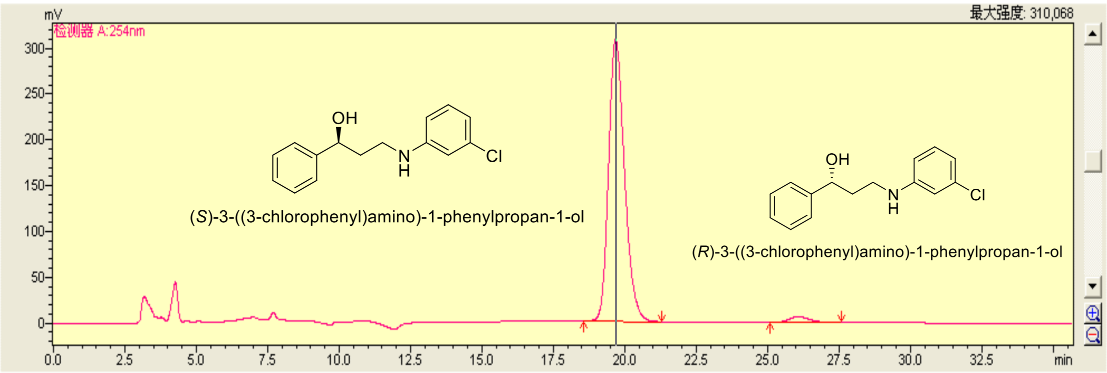

化合物表视图

ID#	名称	保留时间	峰#	面积	高度	面积%
1	RT19. 680	19. 680	1	11934040	308113	97. 3145
2	RT26. 082	26. 082	2	329327	6391	2. 6855

3d: (*S*)-3-((2-chlorophenyl)amino)-1-phenylpropan-1-ol (HPLC (OD-3, elute: Hexane/*i*-PrOH = 97/3, detector: 254 nm, flow rate: 1 mL/min, 25 °C), t_1 = 50.9 min (major), t_2 = 56 min.**)**

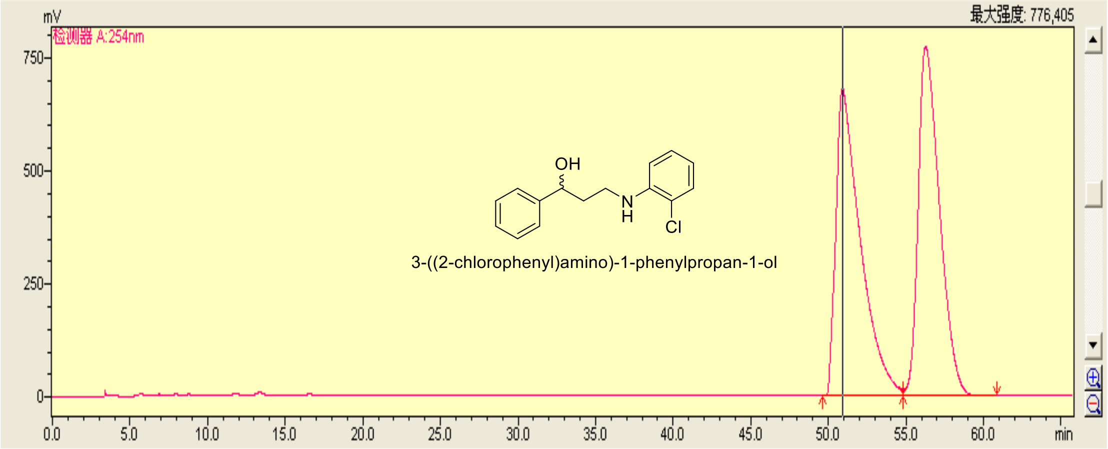

化合物表视图

ID#	名称	保留时间	峰#	面积	高度	面积%
1	RT50.901	50.901	1	74611665	679717	50.1854
2	RT56.266	56.266	2	74060355	772942	49.8146

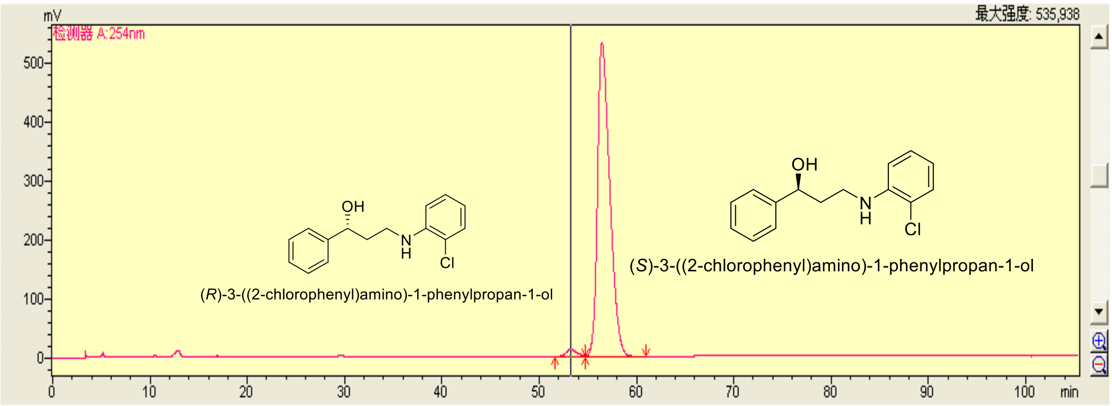

化合物表视图

ID#	名称	保留时间	峰#	面积	高度	面积%
1	RT53.196	53.196	1	946859	12073	1.9981
2	RT56.451	56.451	2	46440159	532180	98.0019

<u>3e:</u> (*S*)-3-((4-bromophenyl)amino)-1-phenylpropan-1-ol (HPLC (OD-H, elute: Hexane/*i*-PrOH = 90/10, detector: 254 nm, flow rate: 1 mL/min, 25 ^{o}C), t_1 = 23.7min (major), t_2 = 29.3 min.)

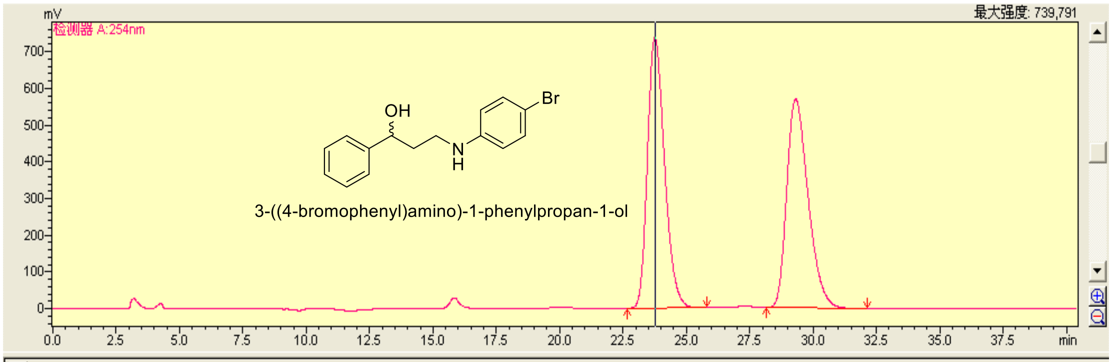

化合物表视图

ID#	名称	保留时间	峰#	面积	高度	面积%
1	RT23.754	23.754	1	34593783	738142	50.7863
2	RT29.312	29.312	2	33522586	569326	49.2137

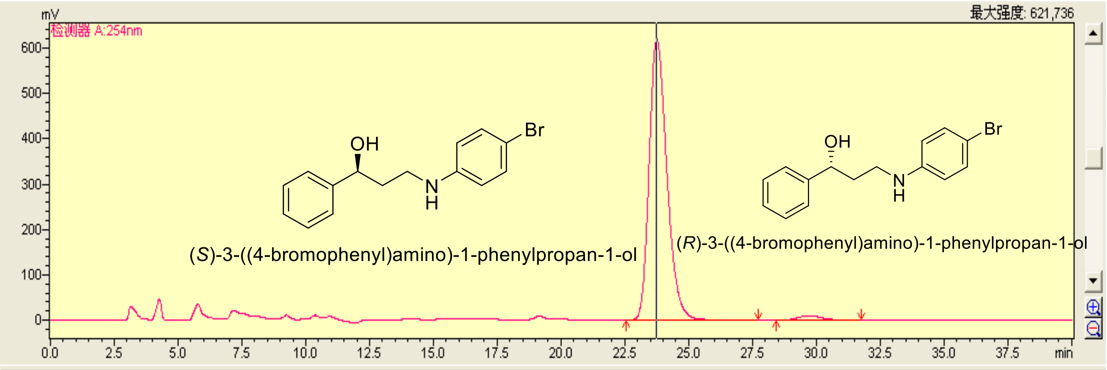

化合物表视图

ID#	名称	保留时间	峰#	面积	高度	面积%
1	RT23.753	23.753	1	29896012	621077	98.1020
2	RT29.718	29.718	2	578396	9849	1.8980

<u>3f:</u> (*S*)-3-((4-nitrophenyl)amino)-1-phenylpropan-1-ol (HPLC (OD-H, elute: Hexane/*i*-PrOH = 90/10, detector: 254 nm, flow rate: 1 mL/min, 25 ^{o}C), t_1 = 42.6min (major), t_2 = 50.4 min.)

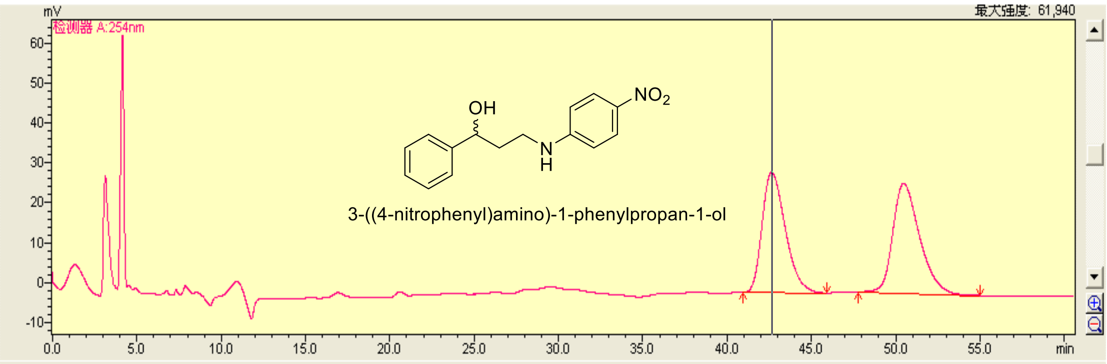

化合物表视图

ID#	名称	保留时间	峰#	面积	高度	面积%
1	RT42.647	42.647	1	2870968	30109	48.5872
2	RT50.472	50.472	2	3037930	27458	51.4128

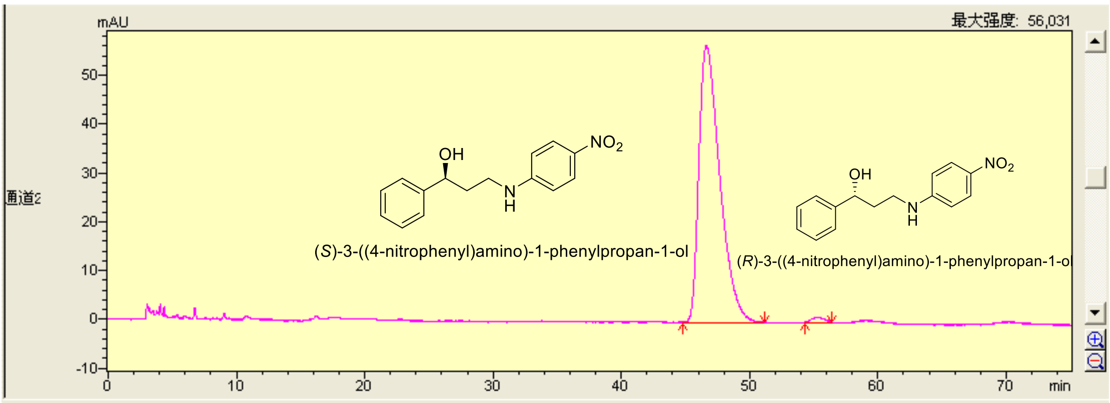

化合物表视图

ID#	峰#	保留时间	面积	面积%	高度	高度%
1	1	46.614	6467913	98.9347	56788	98.1772
2	2	55.270	69644	1.0653	1054	1.8228

3g: (*S*)-3-((3-nitrophenyl)amino)-1-phenylpropan-1-ol (HPLC (OD-H, elute: Hexane/*i*-PrOH = 90/10, detector: 254 nm, flow rate: 1 mL/min, 25 ^{o}C), t_1 = 26.9min (major), t_2 = 31.2min.)

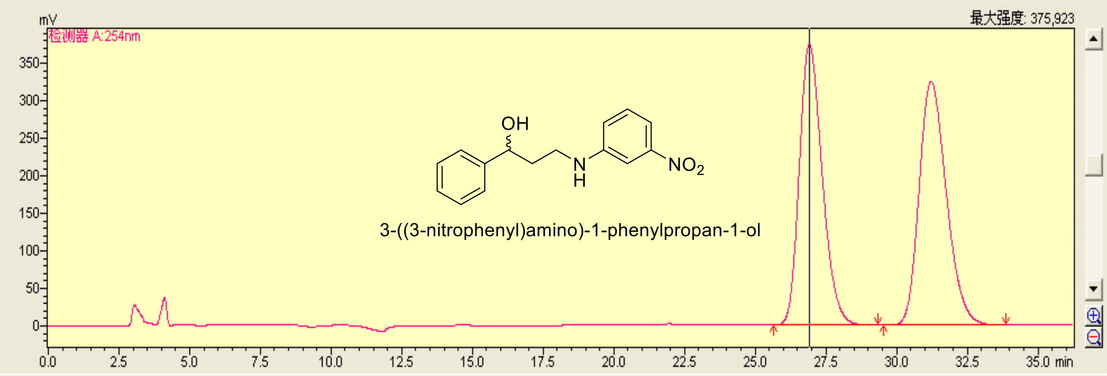

化合物表视图

ID#	名称	保留时间	峰#	面积	高度	面积%
1	RT26.915	26.915	1	21156688	374850	49.2642
2	RT31.221	31.221	2	21788709	325041	50.7358

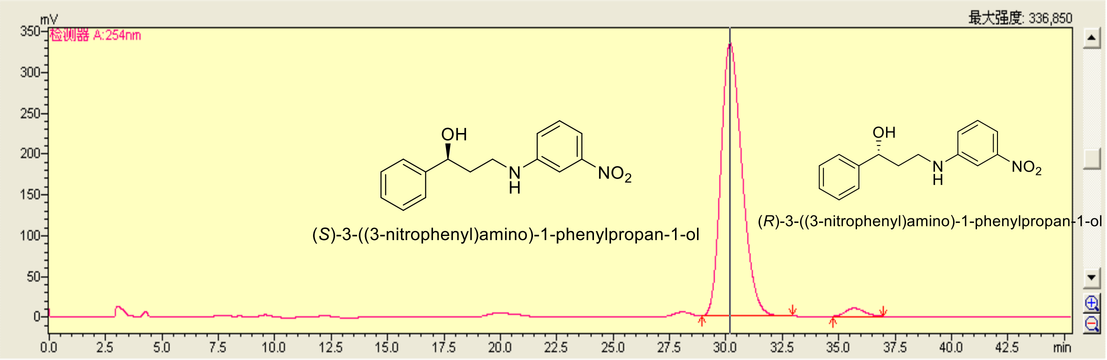

化合物表视图

ID#	名称	保留时间	峰#	面积	高度	面积%
1	RT30.183	30.183	1	21754002	335290	97.4137
2	RT35.688	35.688	2	577569	9919	2.5863

<u>3h:</u> (*S*)-3-((3,4-dimethylphenyl)amino)-1-phenylpropan-1-ol (HPLC (OD-H, elute: Hexane/*i*-PrOH = 90/10, detector: 254 nm, flow rate: 1 mL/min, 25 °C), t_1 = 26.9min (major), t_2 = 37.3 min.**)**

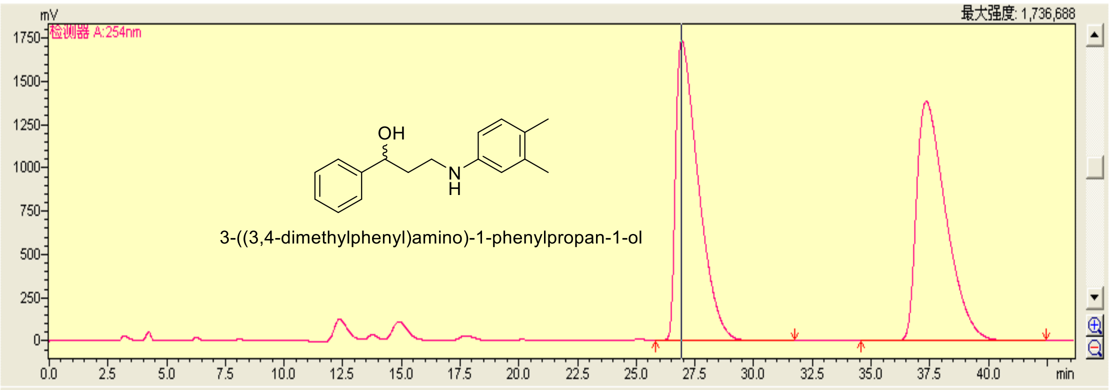

化合物表视图

ID#	名称	保留时间	峰#	面积	高度	面积%
1	RT26. 934	26. 934	1	116467781	1733768	49. 0826
2	RT37. 340	37. 340	2	120821794	1383345	50. 9174

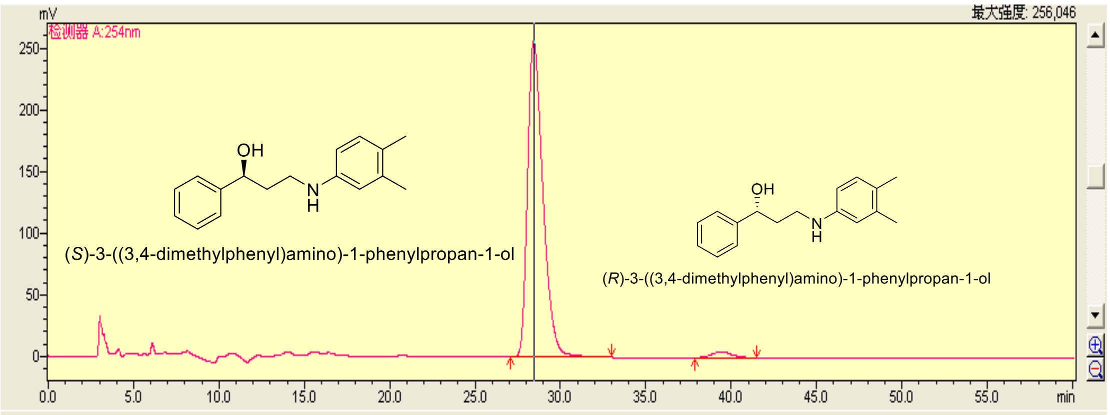

化合物表视图

ID#	名称	保留时间	峰#	面积	高度	面积%
1	RT28. 429	28. 429	1	16194176	256976	97. 7091
2	RT39. 402	39. 402	2	379694	4696	2. 2909

<u>3i:</u> (*S*)-3-((3,5-dimethylphenyl)amino)-1-phenylpropan-1-ol (HPLC (OD-H, elute: Hexane/*i*-PrOH = 90/10, detector: 254 nm, flow rate: 1 mL/min, 25 ^{o}C), t_1 = 15.6min (major), t_2 = 19.8 min.**)**

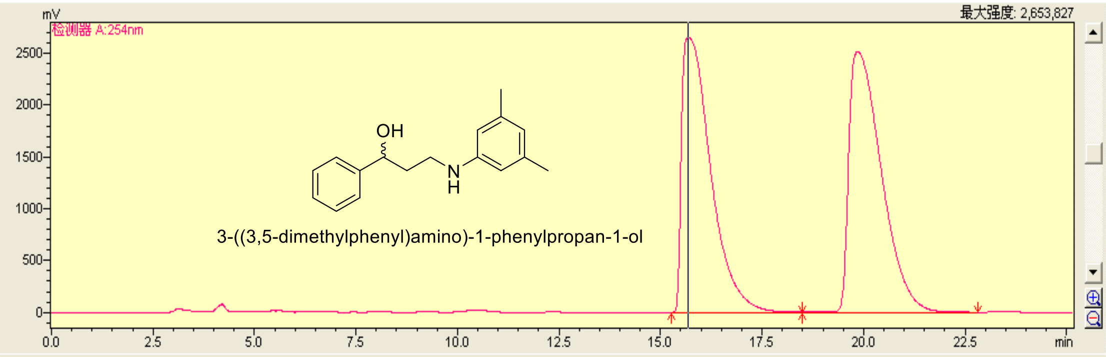

化合物表视图

ID#	名称	保留时间	峰#	面积	高度	面积%
1	RT15.674	15.674	1	132366126	2652983	48.6014
2	RT19.852	19.852	2	139984162	2516708	51.3986

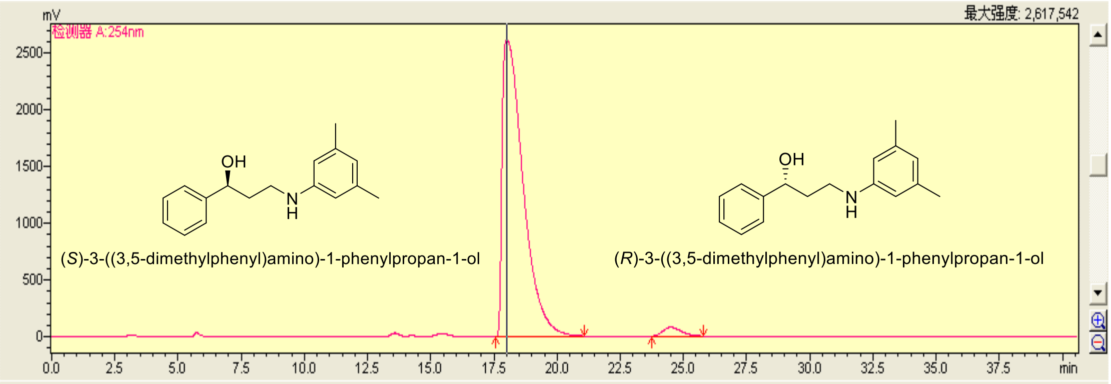

化合物表视图

ID#	名称	保留时间	峰#	面积	高度	面积%
1	RT18.010	18.010	1	151007979	2615602	97.4672
2	RT24.501	24.501	2	3924072	77601	2.5328

3j: (*S*)-3-((3-chloro-4-methylphenyl)amino)-1-phenylpropan-1-ol (HPLC (OD-H, elute: Hexane/*i*-PrOH = 90/10, detector: 254 nm, flow rate: 1 mL/min, 25 °C), t_1 = 18.1min (major), t_2 = 25.3 min.)

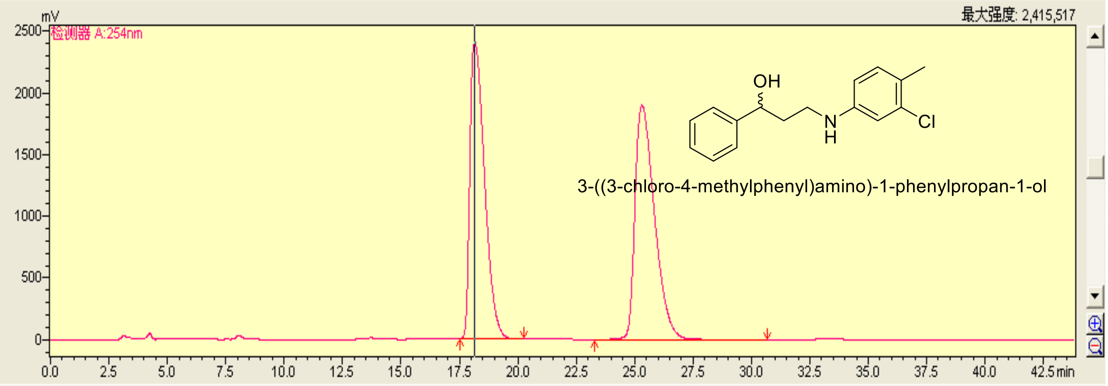

化合物表视图

ID#	名称	保留时间	峰#	面积	高度	面积%
1	RT18.154	18.154	1	105078563	2408630	48.8081
2	RT25.315	25.315	2	110210504	1898812	51.1919

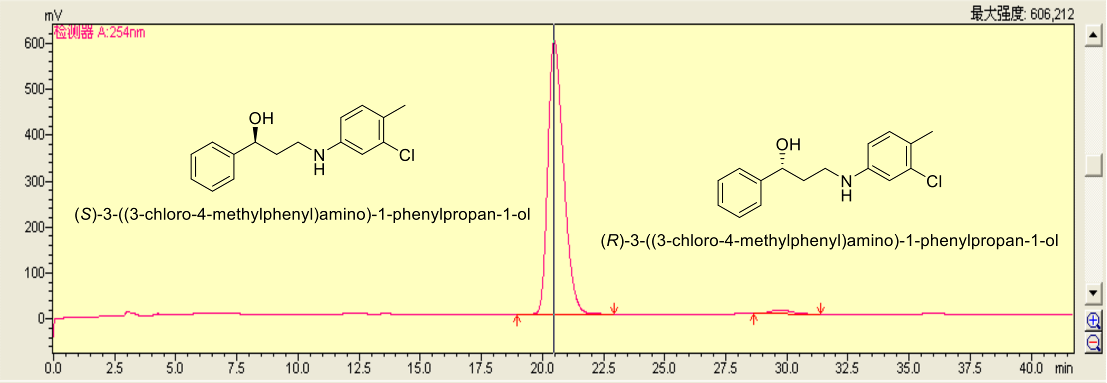

化合物表视图

ID#	名称	保留时间	峰#	面积	高度	面积%
1	RT20.483	20.483	1	26516176	595474	98.0766
2	RT29.713	29.713	2	520027	8750	1.9234

<u>3k:</u> (*S*)-3-((3-methoxyphenyl)amino)-1-phenylpropan-1-ol (HPLC (OD-H, elute: Hexane/*i*-PrOH = 90/10, detector: 254 nm, flow rate: 1 mL/min, 25 ^{o}C), t_1 = 41.7min (major), t_2 = 49.1min.)

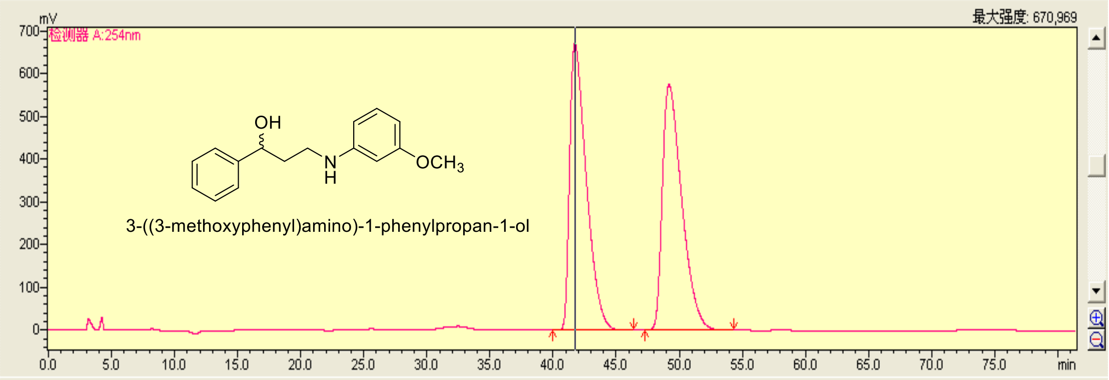

化合物表视图

ID#	名称	保留时间	峰#	面积	高度	面积%
1	RT41.733	41.733	1	61584725	671297	49.9379
2	RT49.193	49.193	2	61737907	574682	50.0621

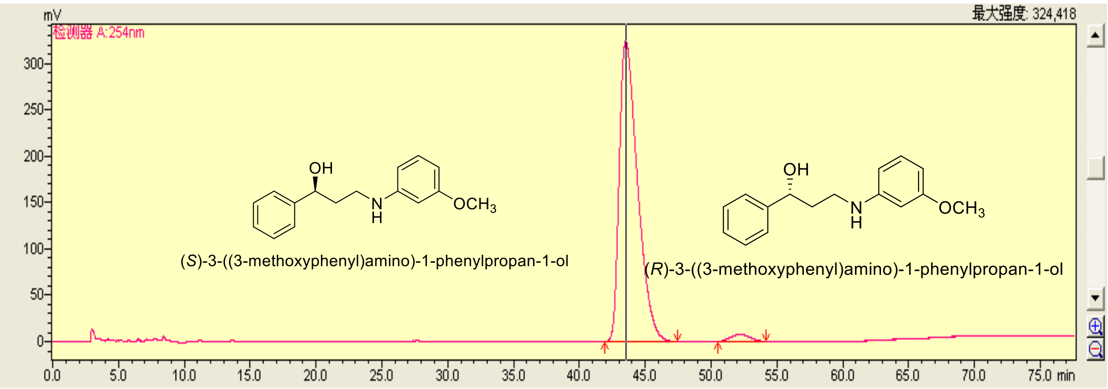

化合物表视图

ID#	名称	保留时间	峰#	面积	高度	面积%
1	RT43.528	43.528	1	31246678	324322	97.5601
2	RT52.142	52.142	2	781439	7854	2.4399

<u>3l:</u> (*S*)-1-(4-fluorophenyl)-3-(phenylamino)propan-1-ol (HPLC (OD-H, elute: Hexane/*i*-PrOH = 90/10, detector: 254 nm, flow rate: 1 mL/min, 25 °C), t_1 = 24.4min (major), t_2 = 28.7min.)

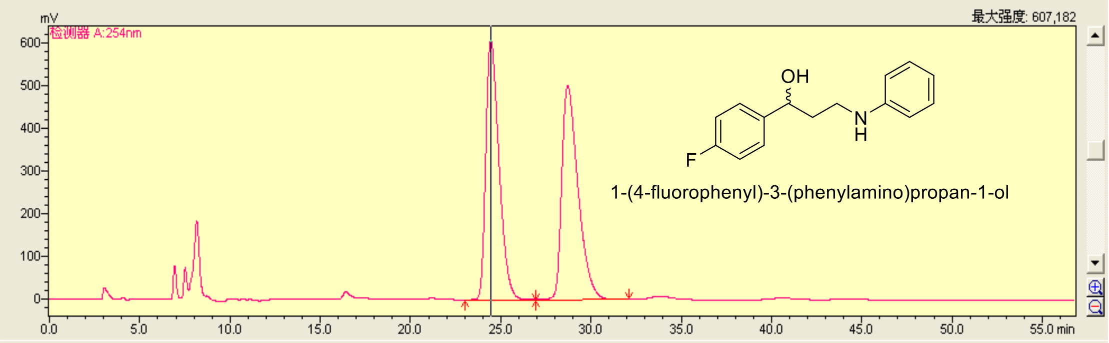

化合物表视图

ID#	名称	保留时间	峰#	面积	高度	面积%
1	RT24. 453	24. 453	1	31036748	607876	49. 6246
2	RT28. 710	28. 710	2	31506318	501318	50. 3754

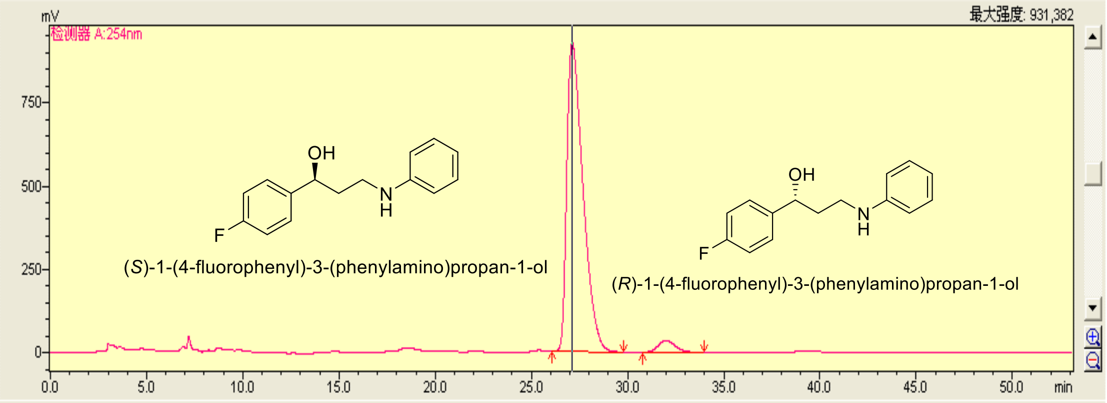

化合物表视图

ID#	名称	保留时间	峰#	面积	高度	面积%
1	RT27. 122	27. 122	1	54388487	928757	96. 1630
2	RT31. 988	31. 988	2	2170173	34427	3. 8370

3m: **(*S*)-1-(4-chlorophenyl)-3-(phenylamino)propan-1-ol** (HPLC (OD-H, elute: Hexane/*i*-PrOH = 90/10, detector: 254 nm, flow rate: 1 mL/min, 25 °C), t_1 = 26.1min (major), t_2 = 31.7min.)

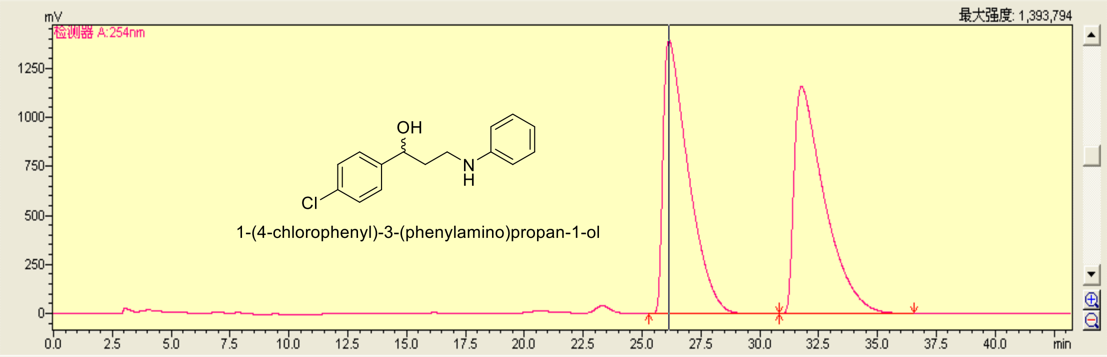

化合物表视图

ID#	名称	保留时间	峰#	面积	高度	面积%
1	RT26.144	26.144	1	103556646	1393644	49.9324
2	RT31.778	31.778	2	103837200	1155508	50.0676

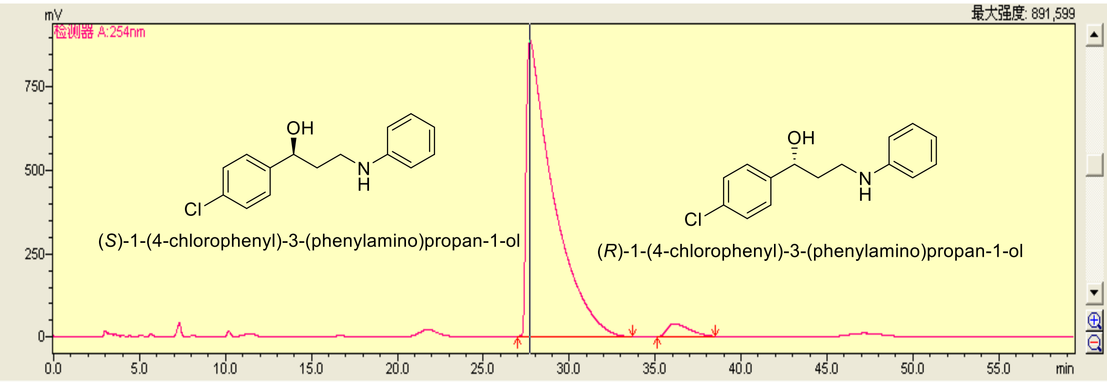

化合物表视图

ID#	名称	保留时间	峰#	面积	高度	面积%
1	RT27.720	27.720	1	97221566	889117	96.5274
2	RT36.162	36.162	2	3497569	39238	3.4726

<u>3n:</u> (*S*)-1-(4-bromophenyl)-3-(phenylamino)propan-1-ol (HPLC (OD-H, elute: Hexane/*i*-PrOH = 90/10, detector: 254 nm, flow rate: 1 mL/min, 25 °C), t_1 = 28.3min (major), t_2 = 35.2min.)

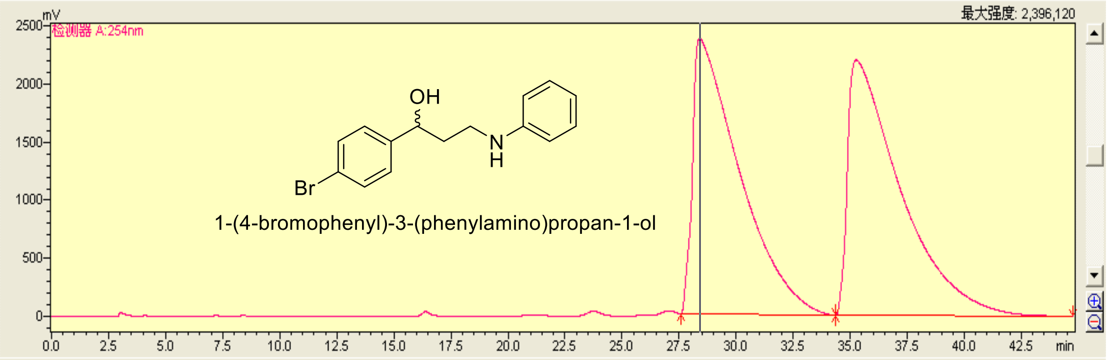

化合物表视图

ID#	名称	保留时间	峰#	面积	高度	面积%
1	RT28.398	28.398	1	341241119	2378339	49.0772
2	RT35.263	35.263	2	354074559	2201553	50.9228

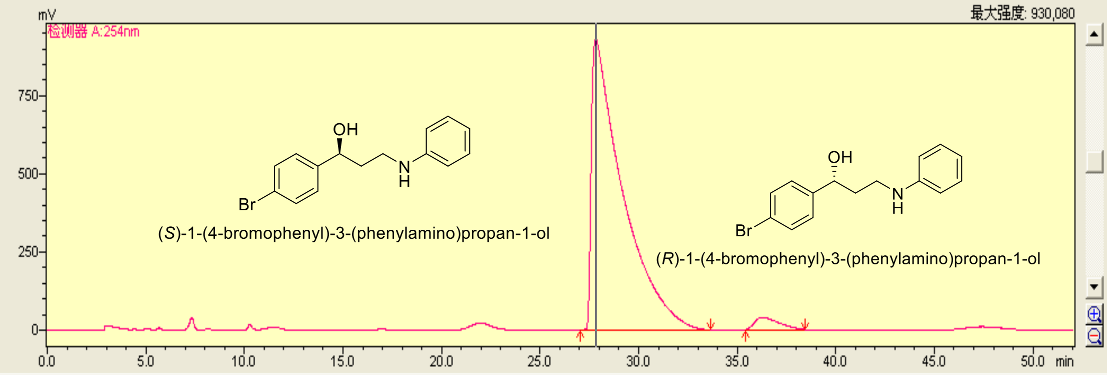

化合物表视图

ID#	名称	保留时间	峰#	面积	高度	面积%
1	RT27.825	27.825	1	102425641	929489	96.8306
2	RT36.319	36.319	2	3352508	38898	3.1694

3o: **(*S*)-1-(4-iodophenyl)-3-(phenylamino)propan-1-ol** (HPLC (AS-H, elute: Hexane/*i*-PrOH = 90/10, detector: 254 nm, flow rate: 1 mL/min, 25 ^{o}C), t_1 = 13.8min (major), t_2 = 16.5min.)

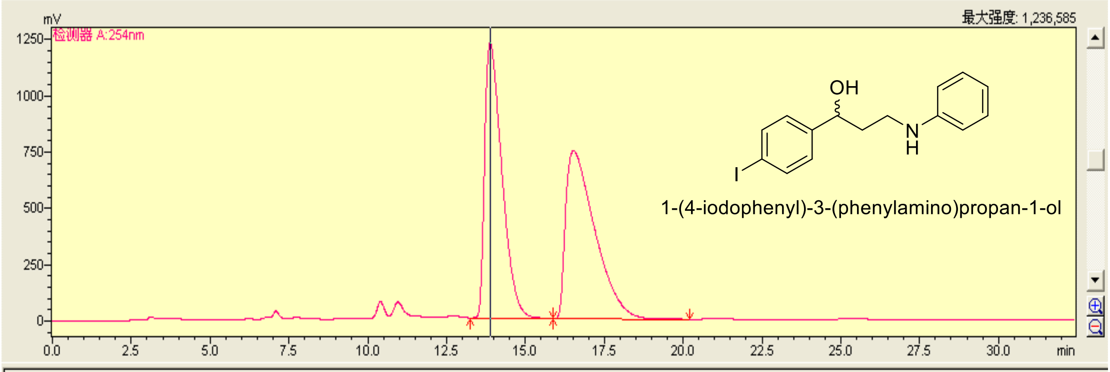

化合物表视图

ID#	名称	保留时间	峰#	面积	高度	面积%
1	RT13.879	13.879	1	46812364	1223397	49.2261
2	RT16.523	16.523	2	48284340	747224	50.7739

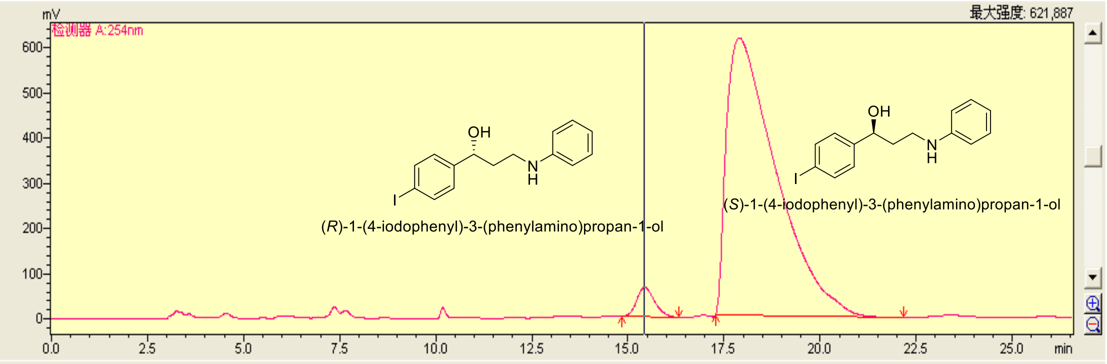

化合物表视图

ID#	名称	保留时间	峰#	面积	高度	面积%
1	RT15.426	15.426	1	2104418	64760	3.6504
2	RT17.896	17.896	2	55545240	614113	96.3496

3p: **(*S*)-3-(phenylamino)-1-(p-tolyl)propan-1-ol** (HPLC (OD-H, elute: Hexane/*i*-PrOH = 90/10, detector: 254 nm, flow rate: 1 mL/min, 25 °C), t_1 = 22.8min (major), t_2 = 28.0min.)

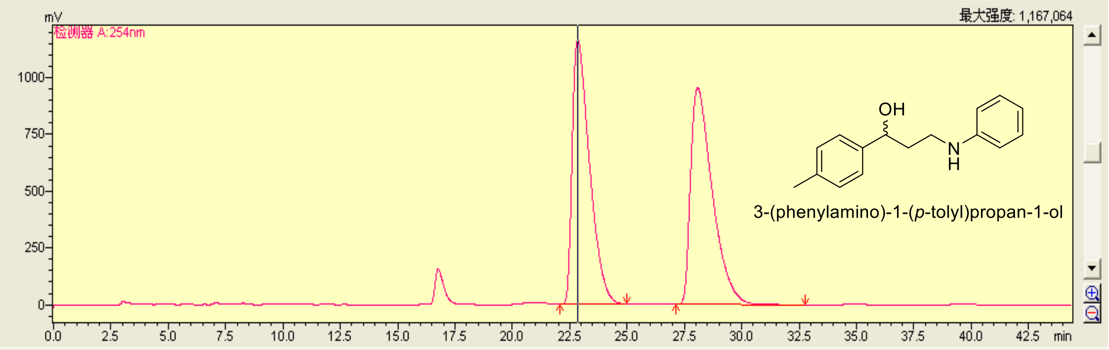

化合物表视图

ID#	名称	保留时间	峰#	面积	高度	面积%
1	RT22.852	22.852	1	61347146	1164899	49.3407
2	RT28.077	28.077	2	62986699	953503	50.6593

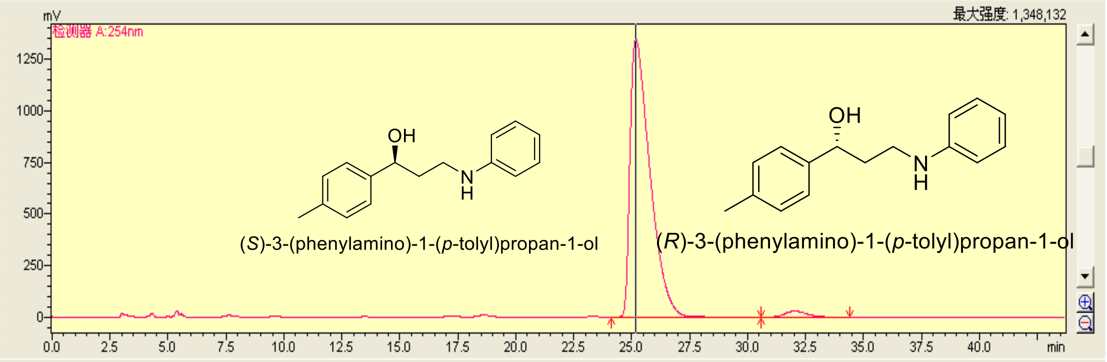

化合物表视图

ID#	名称	保留时间	峰#	面积	高度	面积%
1	RT25.178	25.178	1	80438686	1347360	97.6682
2	RT32.030	32.030	2	1920476	29442	2.3318

3q: (*S*)-1-(4-methoxyphenyl)-3-(phenylamino)propan-1-ol (HPLC (OD-H, elute: Hexane/*i*-PrOH = 90/10, detector: 254 nm, flow rate: 1 mL/min, 25 ^{o}C), t_1 = 36.6min (major), t_2 = 43.4min.**)**

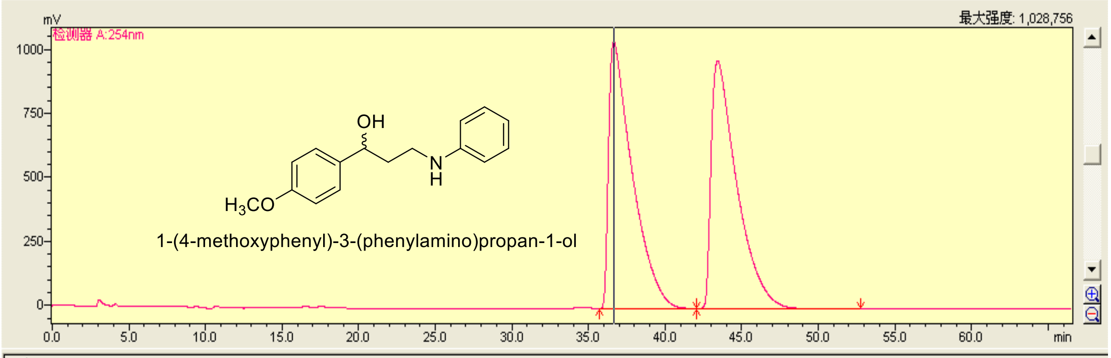

化合物表视图

ID#	名称	保留时间	峰#	面积	高度	面积%
1	RT36. 650	36. 650	1	112235263	1041298	49. 7672
2	RT43. 428	43. 428	2	113285176	968173	50. 2328

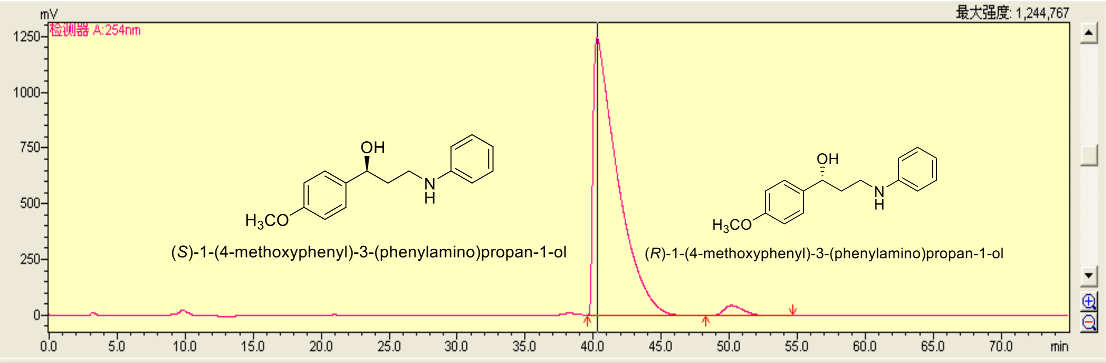

化合物表视图

ID#	名称	保留时间	峰#	面积	高度	面积%
1	RT40. 287	40. 287	1	155597031	1245609	97. 0517
2	RT50. 175	50. 175	2	4726861	45845	2. 9483

Table S2. Reusability of catalyst **1** for the aza–Michael addition/ATH one–pot Enantioselective tandem reaction of 1–phenylprop–2–enone and aniline.[a]

**Entry**	**1**	**2**	**3**	**4**	**5**	**6**	**7**
**Yield [%]**	91	91	90	90	90	86	78
**ee [%]**	96	96	96	96	96	96	96

[a] Reaction conditions: catalyst **5** (196.80 mg), K_2CO_3 (138.0 mg, 1.0 mmol), HCO_2Na (680.0 mg, 10.0 mmol), iodoacetophenones (1.0 mmol), aryne (1.10 mmol), and 40.0 mL of the mixed solvents (H_2O/MeOH v/v = 1/3), reaction temperature (60 ℃), reaction time (16 h).

Figure S7. Reusability of catalyst **1** for the aza–Michael addition/ATH one–pot Enantioselective tandem reaction of 1–phenylprop–2–enone and aniline to (*S*)–1–phenyl–3–(phenylamino)propanol.

Recycle 1

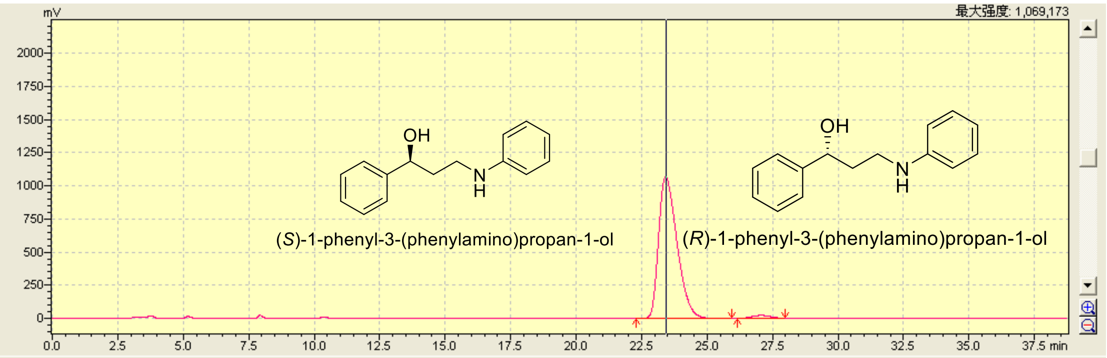

化合物表视图

ID#	名称	保留时间	峰#	面积	高度	面积%
1	RT23. 417	23. 417	1	52098884	1069661	97. 9800
2	RT27. 050	27. 050	2	1074076	21950	2. 0200

Recycle 2

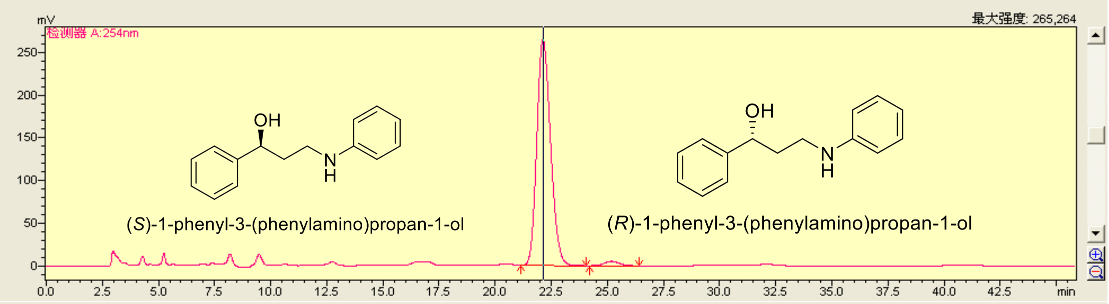

化合物表视图

ID#	名称	保留时间	峰#	面积	高度	面积%
1	RT22. 128	22. 128	1	11219063	264696	97. 8759
2	RT25. 185	25. 185	2	243473	5124	2. 1241

Recycle 3

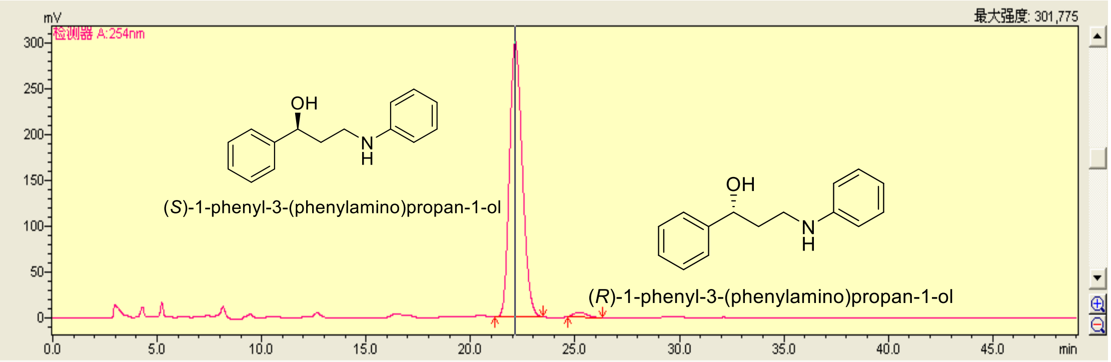

化合物表视图

ID#	名称	保留时间	峰#	面积	高度	面积%
1	RT22.138	22.138	1	12821020	301148	98.3425
2	RT25.215	25.215	2	216091	5120	1.6575

Recycle 4

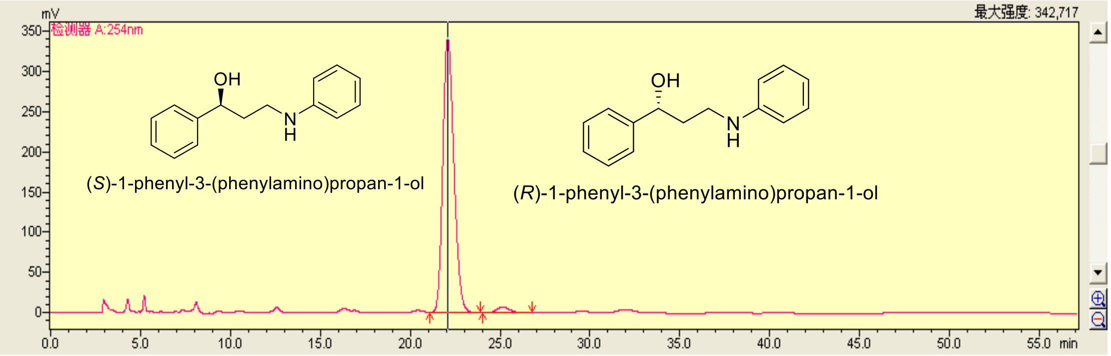

化合物表视图

ID#	名称	保留时间	峰#	面积	高度	面积%
1	RT22.083	22.083	1	14586268	342323	97.8818
2	RT25.161	25.161	2	315649	6701	2.1182

Recycle 5

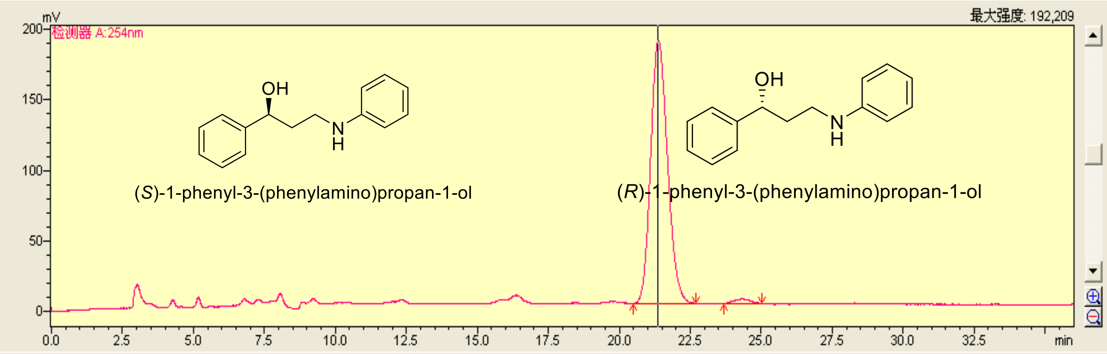

化合物表视图

ID#	名称	保留时间	峰#	面积	高度	面积%
1	RT21.373	21.373	1	7691994	186358	98.3673
2	RT24.303	24.303	2	127673	3294	1.6327

Recycle 6

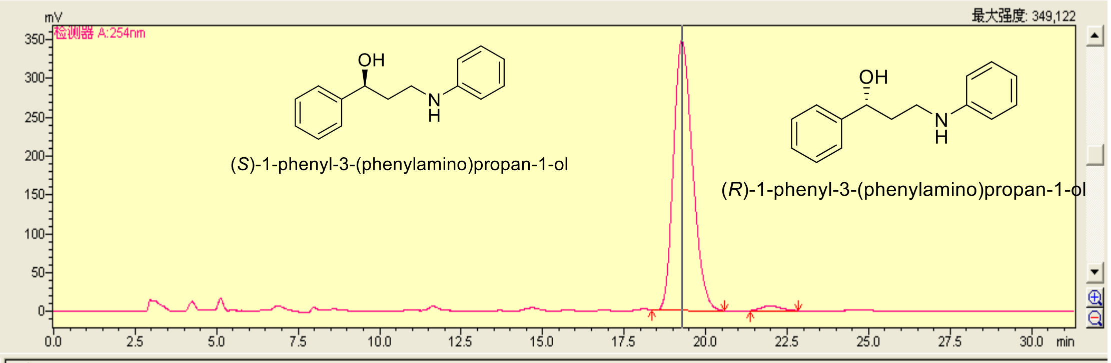

化合物表视图

ID#	名称	保留时间	峰#	面积	高度	面积%
1	RT19.269	19.269	1	14134191	347386	98.0954
2	RT21.956	21.956	2	274424	6420	1.9046

Recycle 7

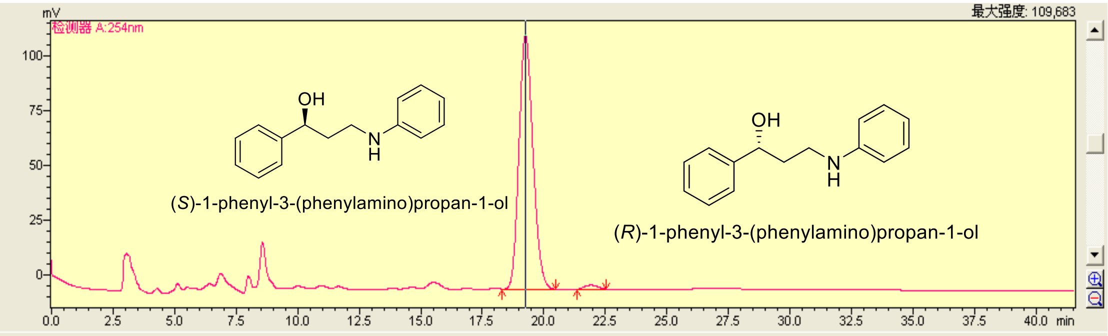

化合物表视图

ID#	名称	保留时间	峰#	面积	高度	面积%
1	RT19.249	19.249	1	4593451	116219	98.5234
2	RT21.903	21.903	2	68843	1826	1.4766

Figure S8. Characterizations of chiral products.

<u>3a:</u> (*S*)-1-phenyl-3-(phenylamino)propan-1-ol

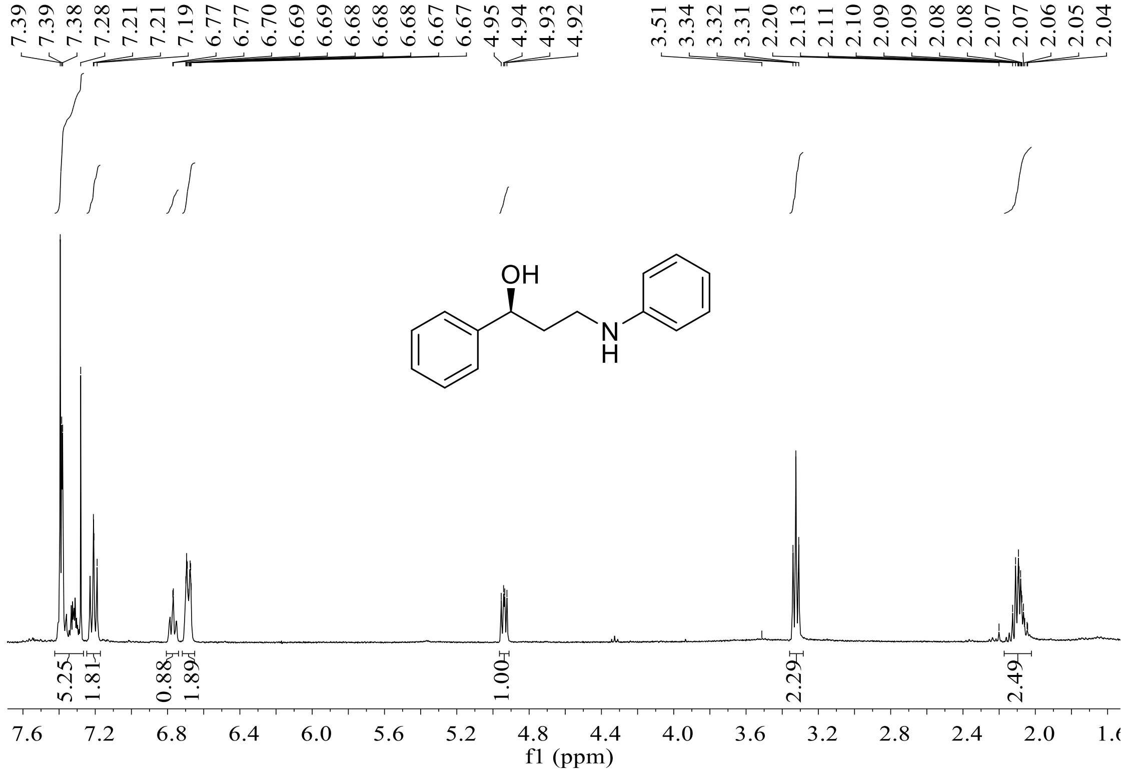

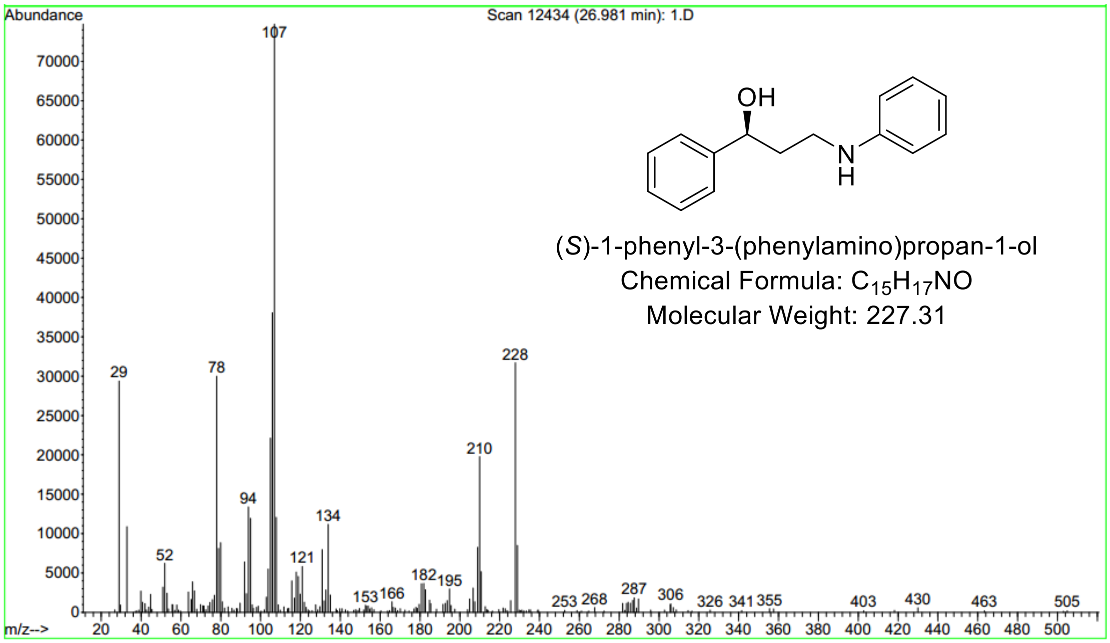

3b: (*S*)-3-((4-chlorophenyl)amino)-1-phenylpropan-1-ol

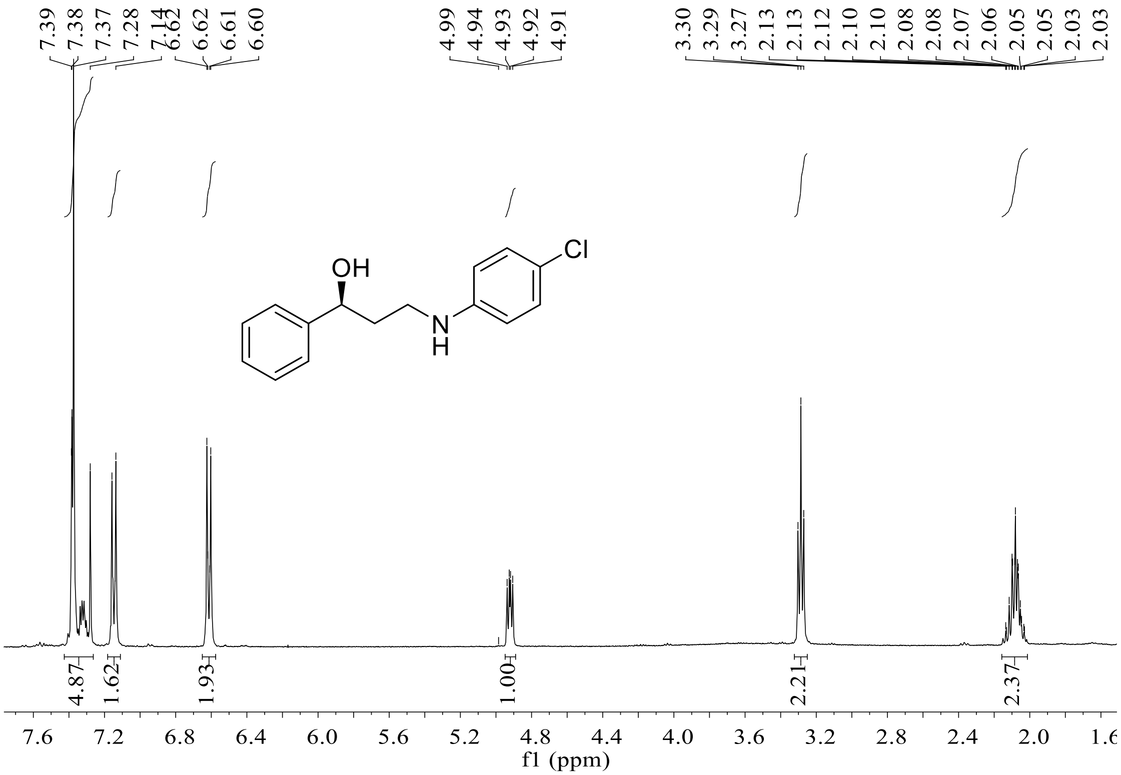

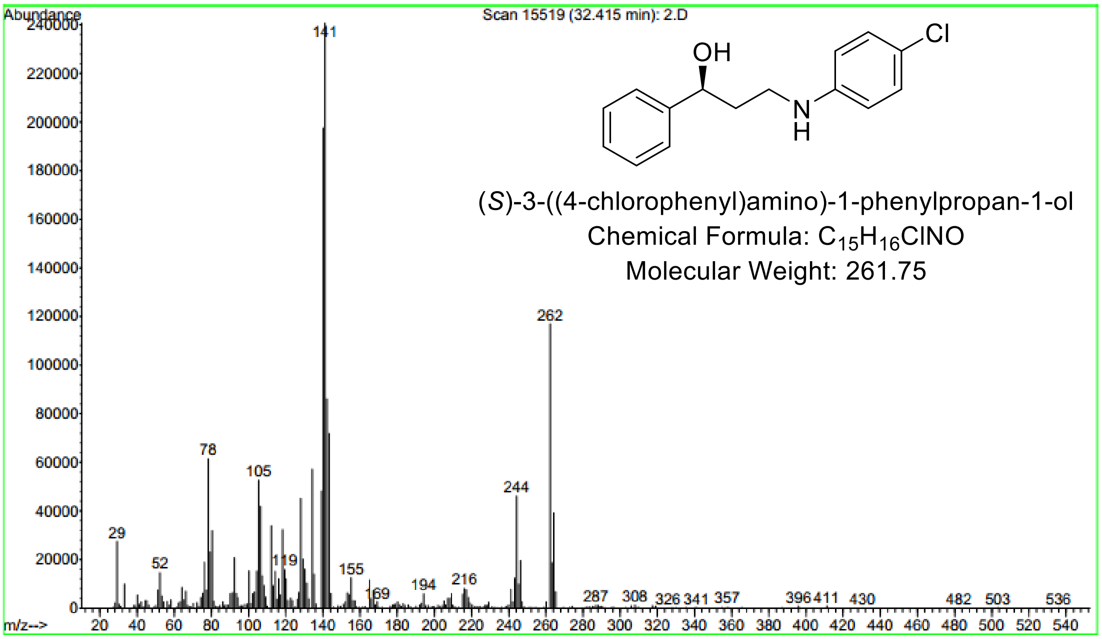

3c: (*S*)-3-((3-chlorophenyl)amino)-1-phenylpropan-1-ol

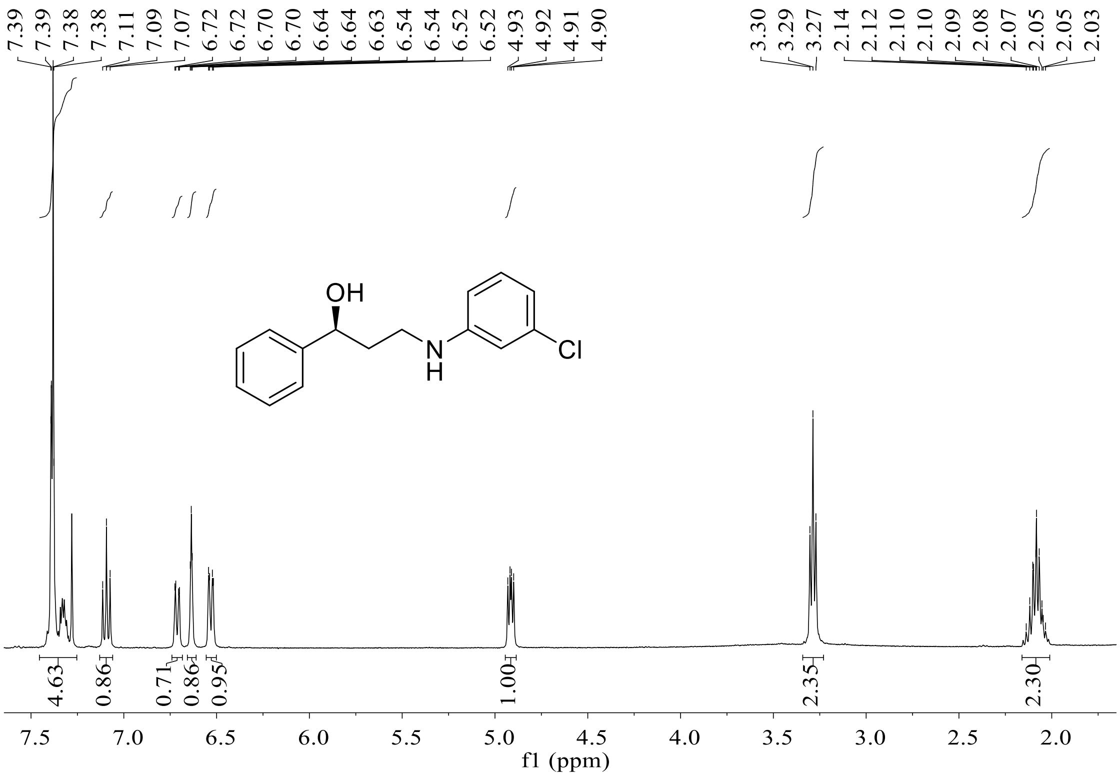

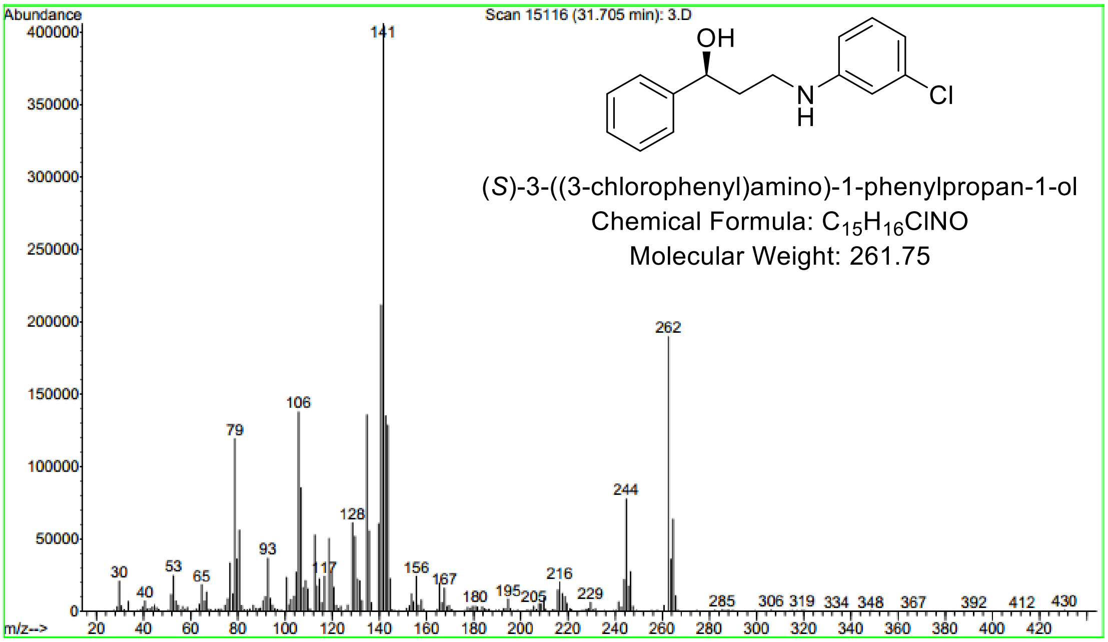

3d: (*S*)-3-((2-chlorophenyl)amino)-1-phenylpropan-1-ol

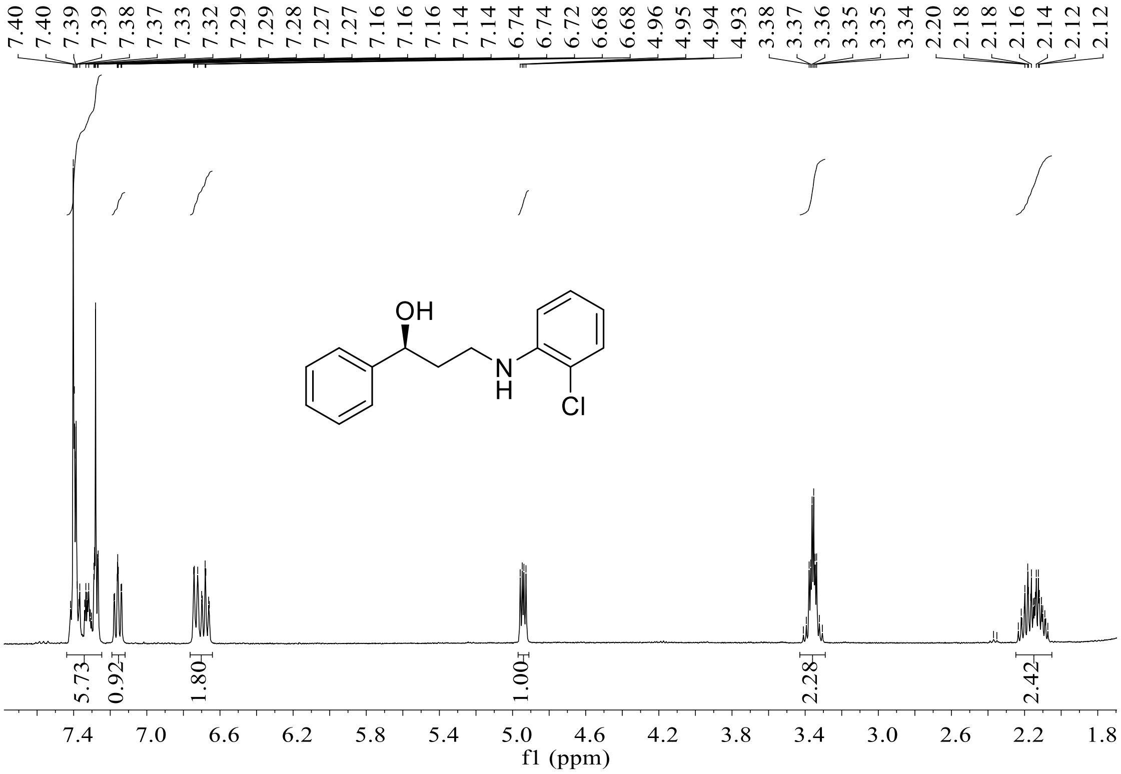

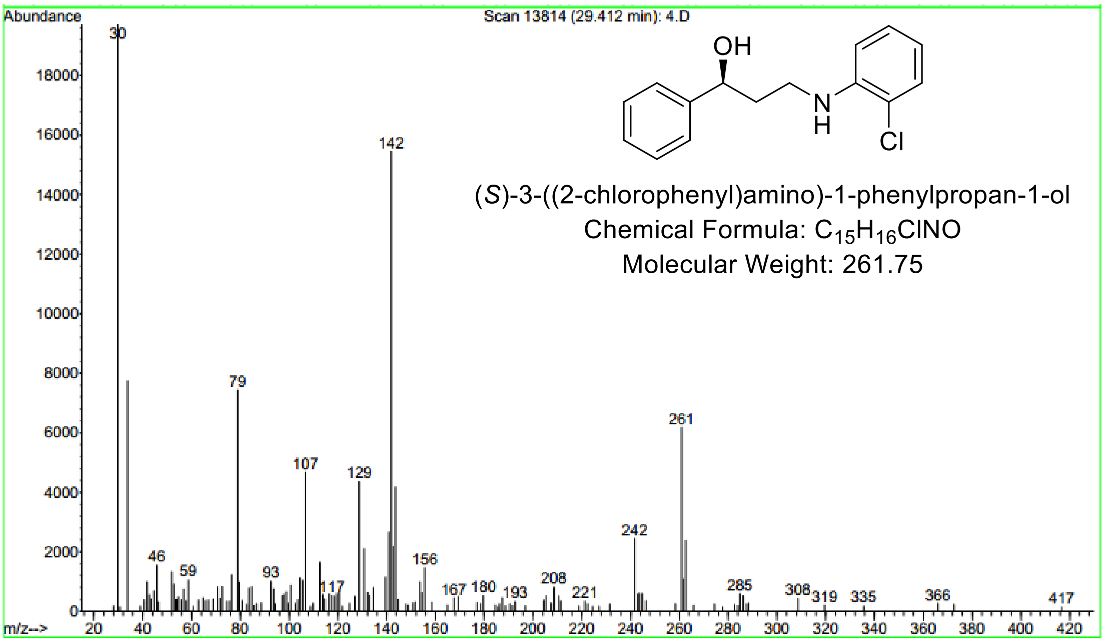

3e: (*S*)-3-((4-bromophenyl)amino)-1-phenylpropan-1-ol

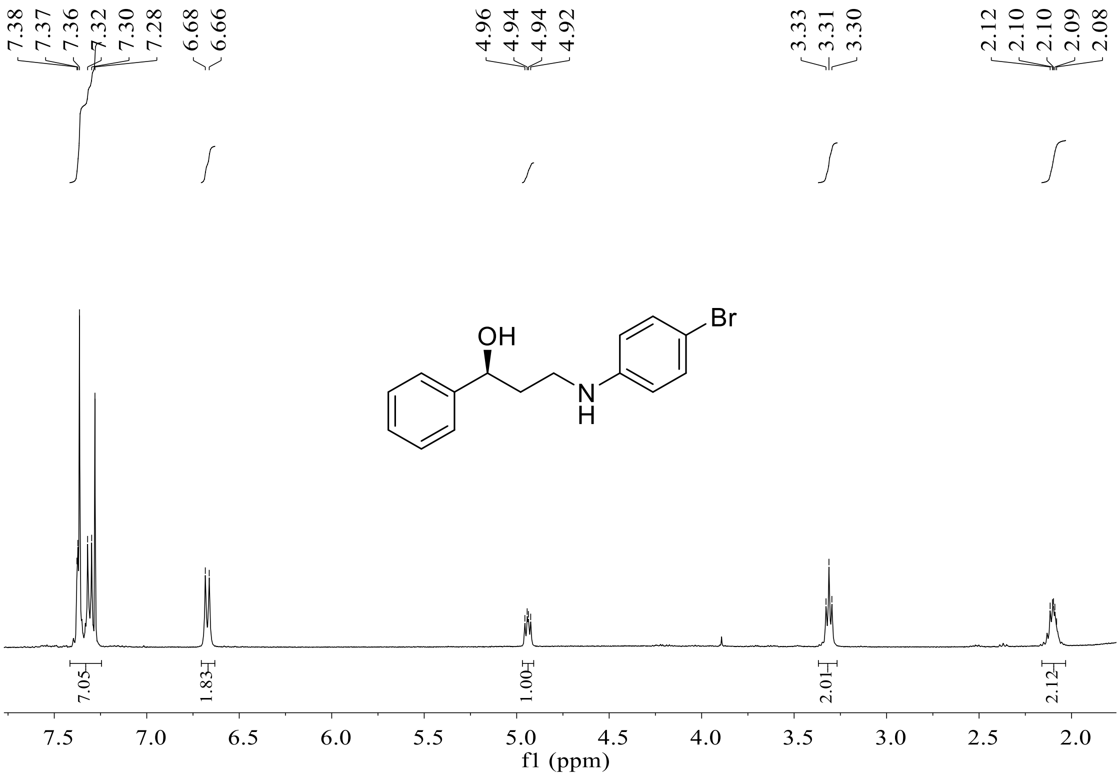

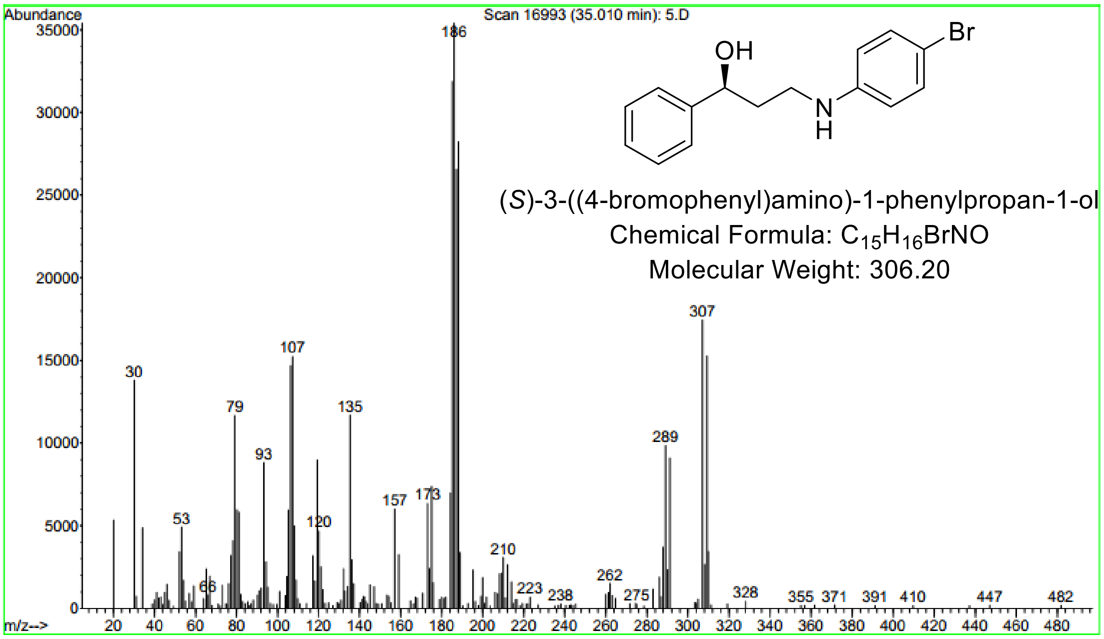

<u>3f:</u> (*S*)-3-((4-nitrophenyl)amino)-1-phenylpropan-1-ol

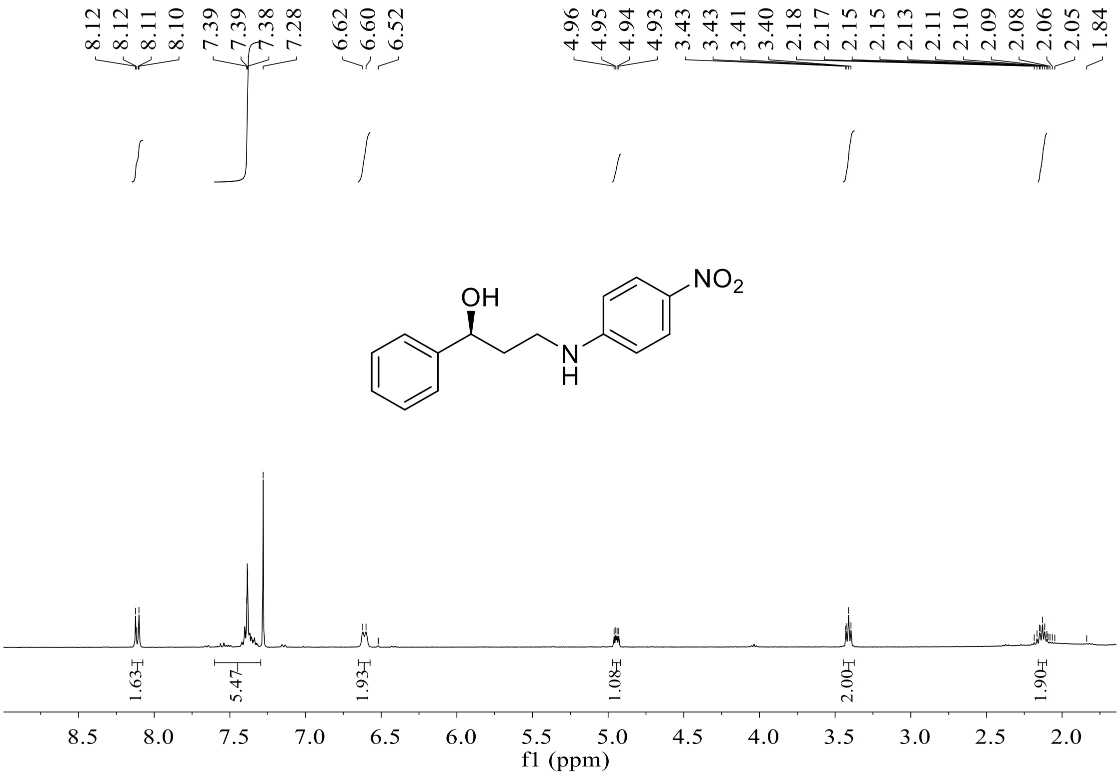

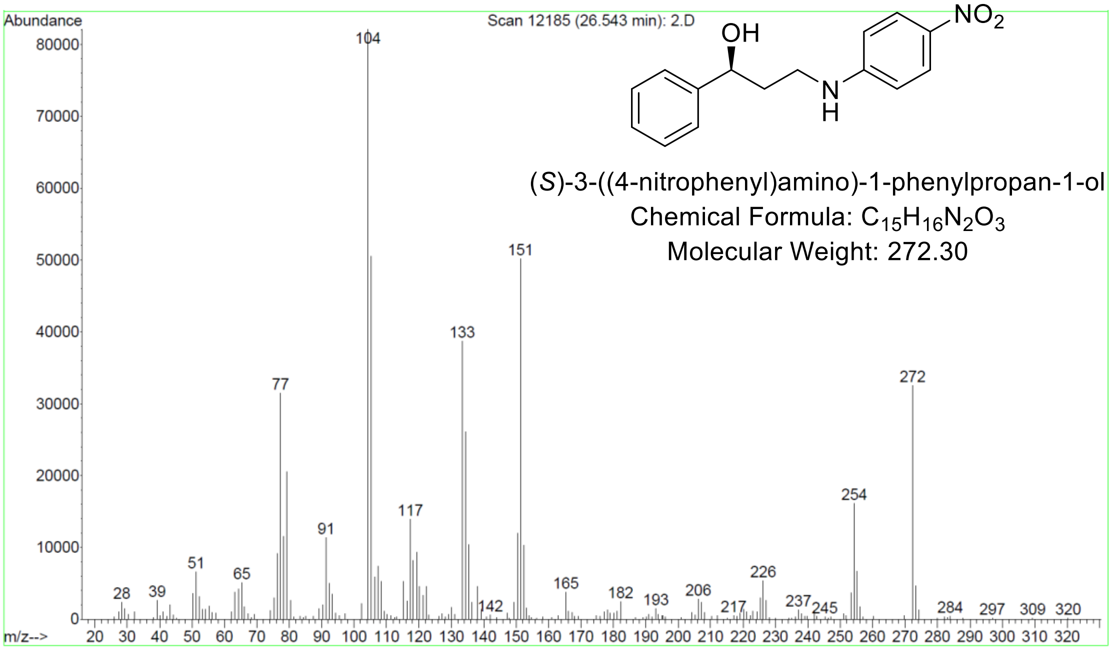

3g: (*S*)-3-((3-nitrophenyl)amino)-1-phenylpropan-1-ol

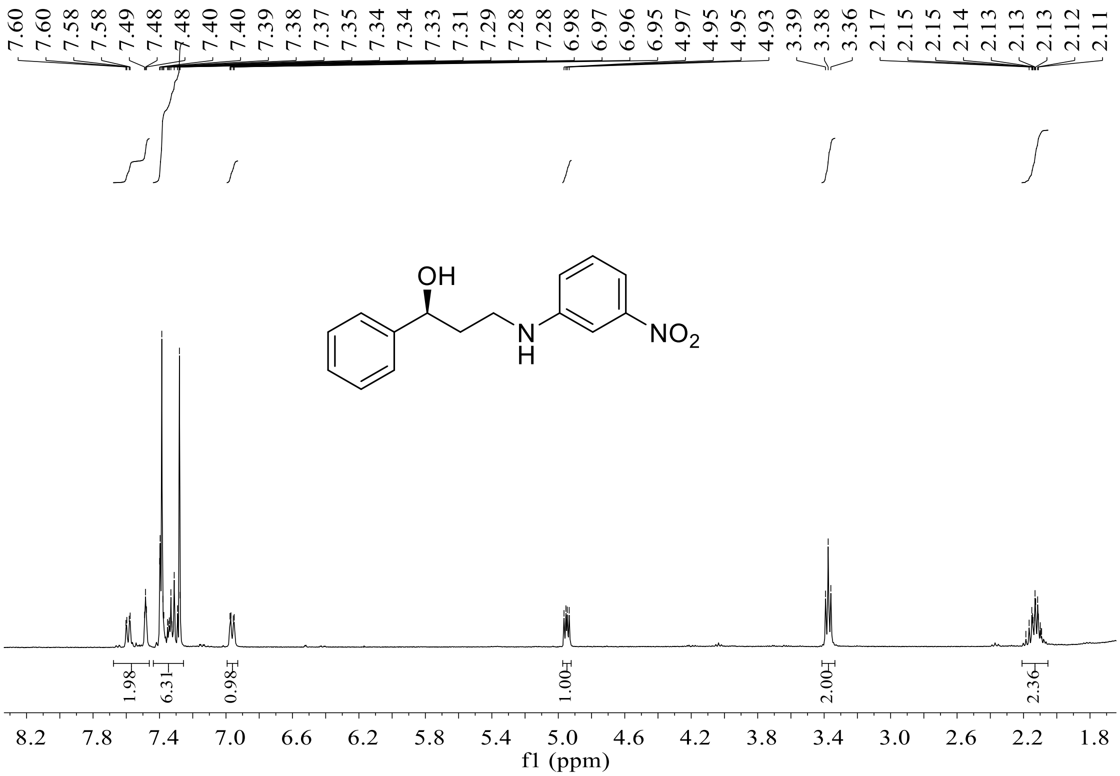

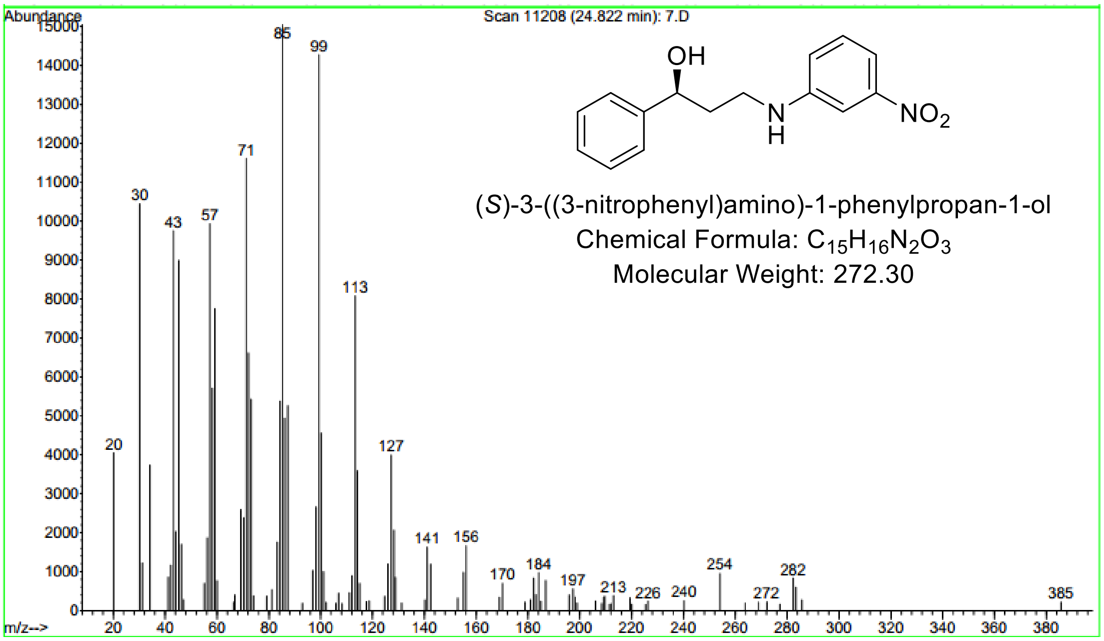

<u>3h:</u> (*S*)-3-((3,4-dimethylphenyl)amino)-1-phenylpropan-1-ol

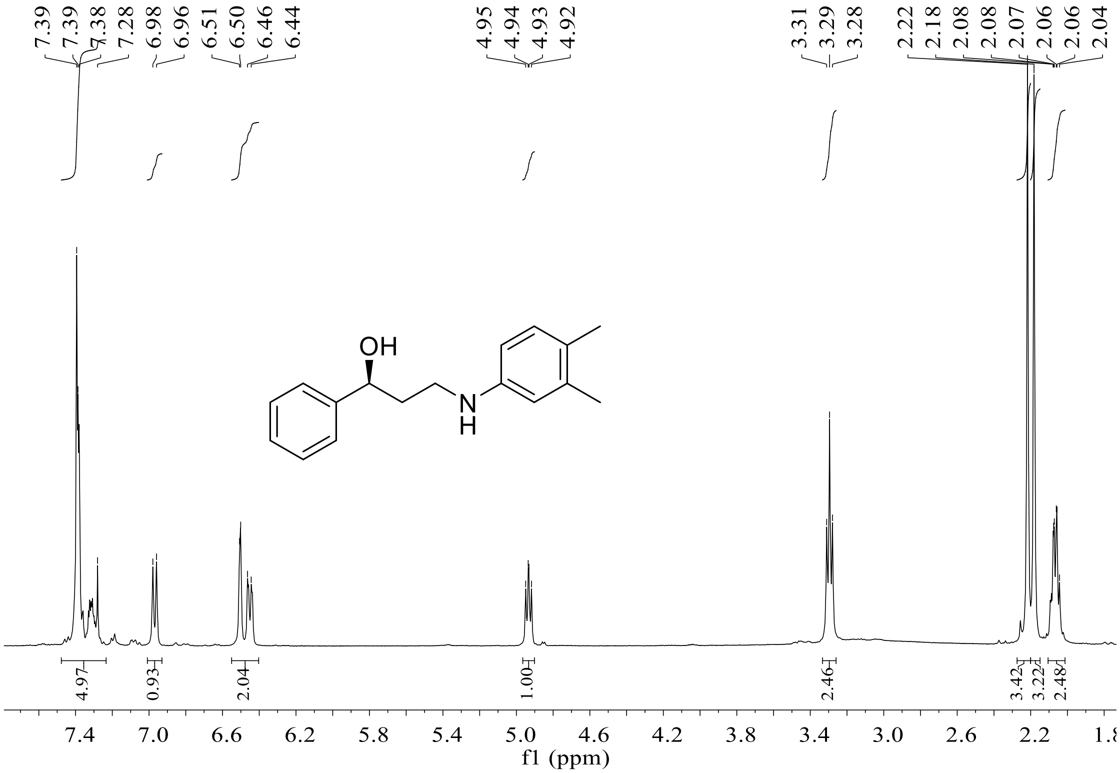

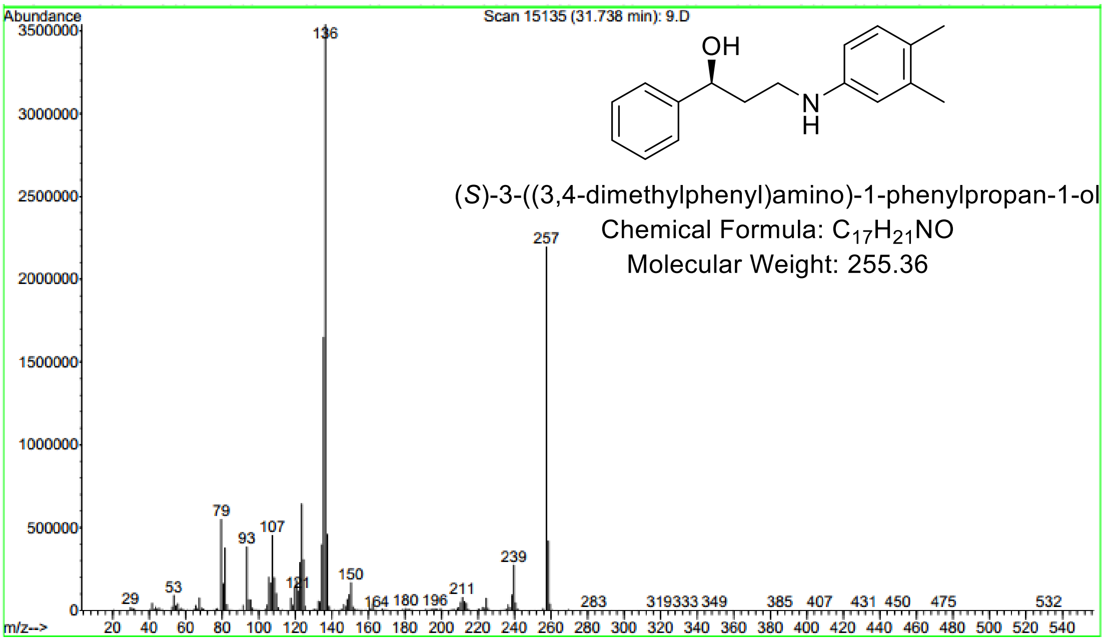

3i: (*S*)-3-((3,5-dimethylphenyl)amino)-1-phenylpropan-1-ol

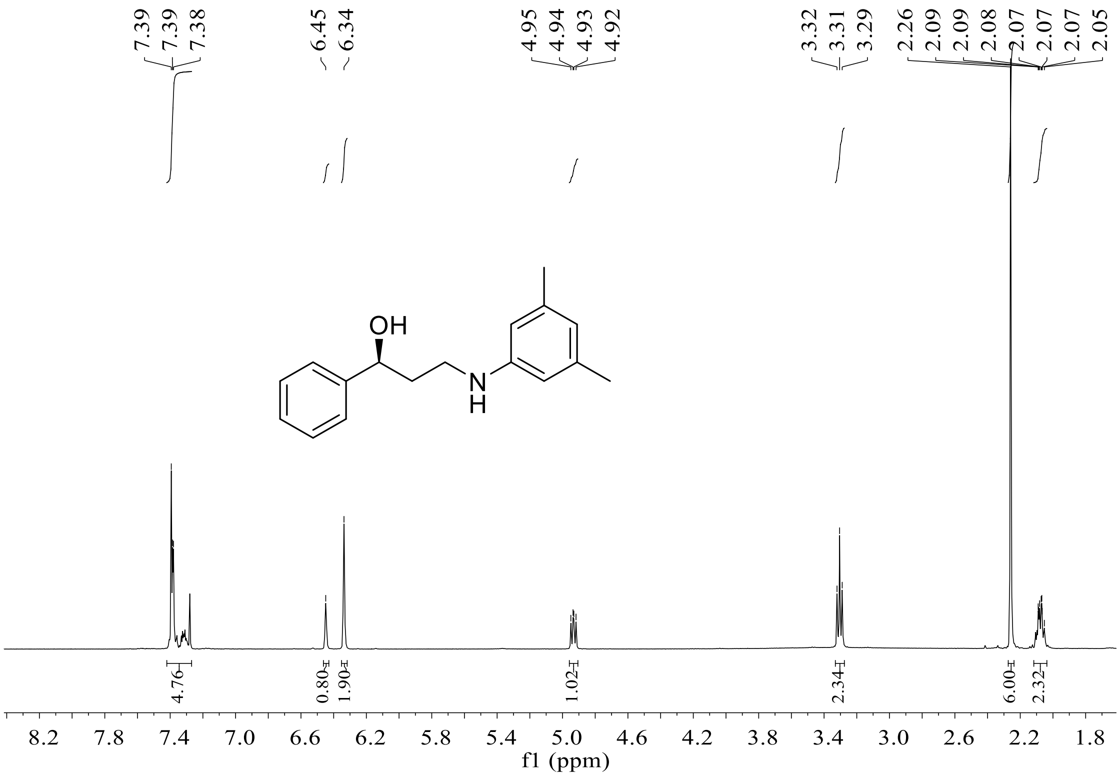

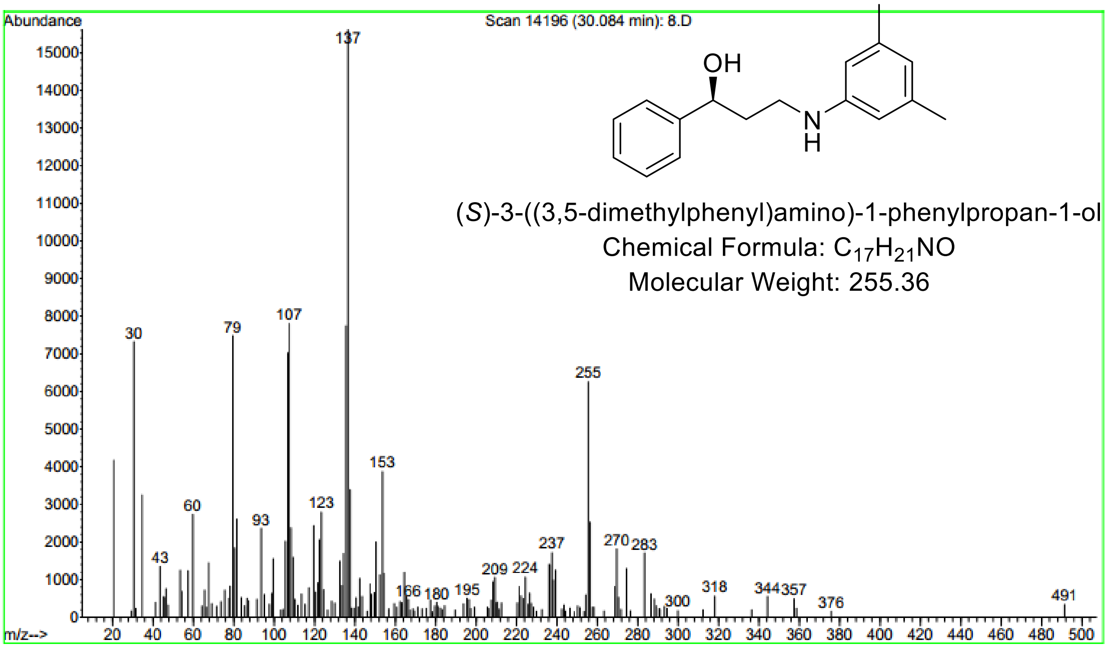

3j: (*S*)-3-((3-chloro-4-methylphenyl)amino)-1-phenylpropan-1-ol

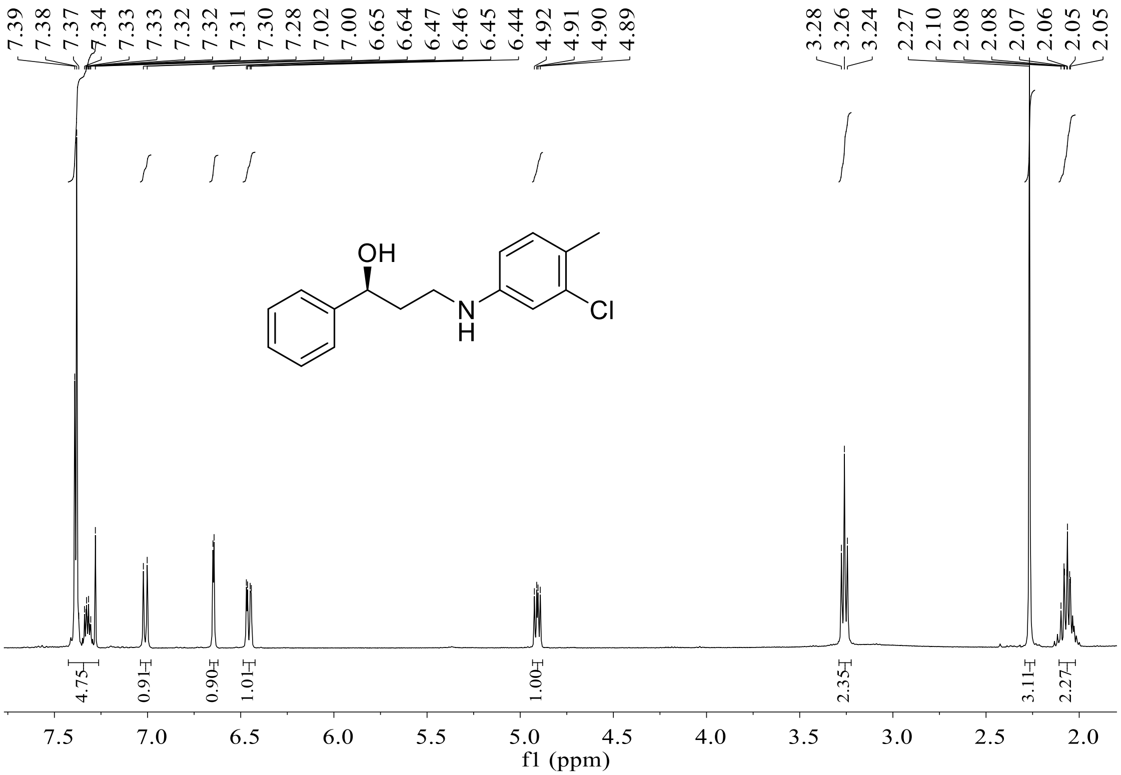

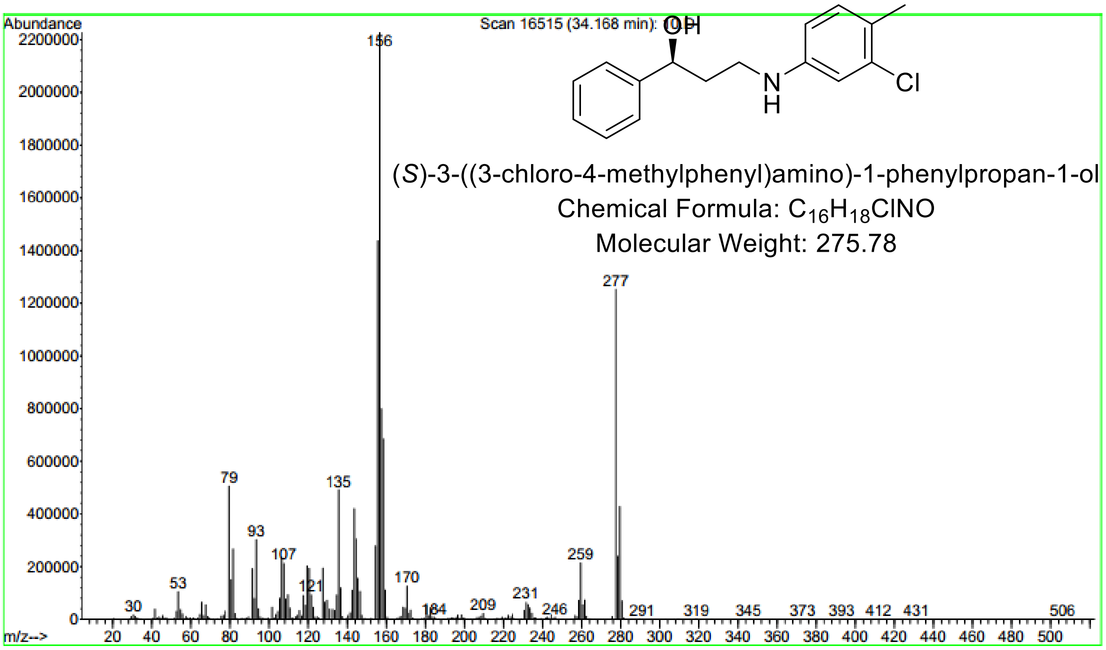

<u>3k:</u> (*S*)-3-((3-methoxyphenyl)amino)-1-phenylpropan-1-ol

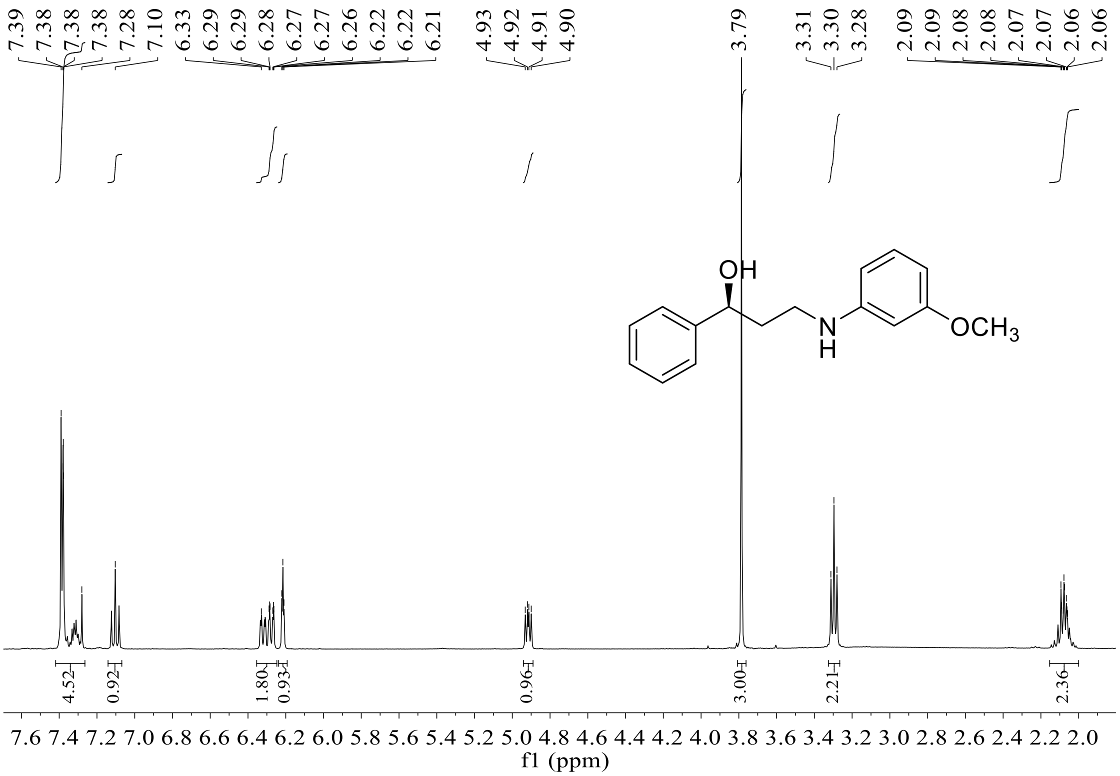

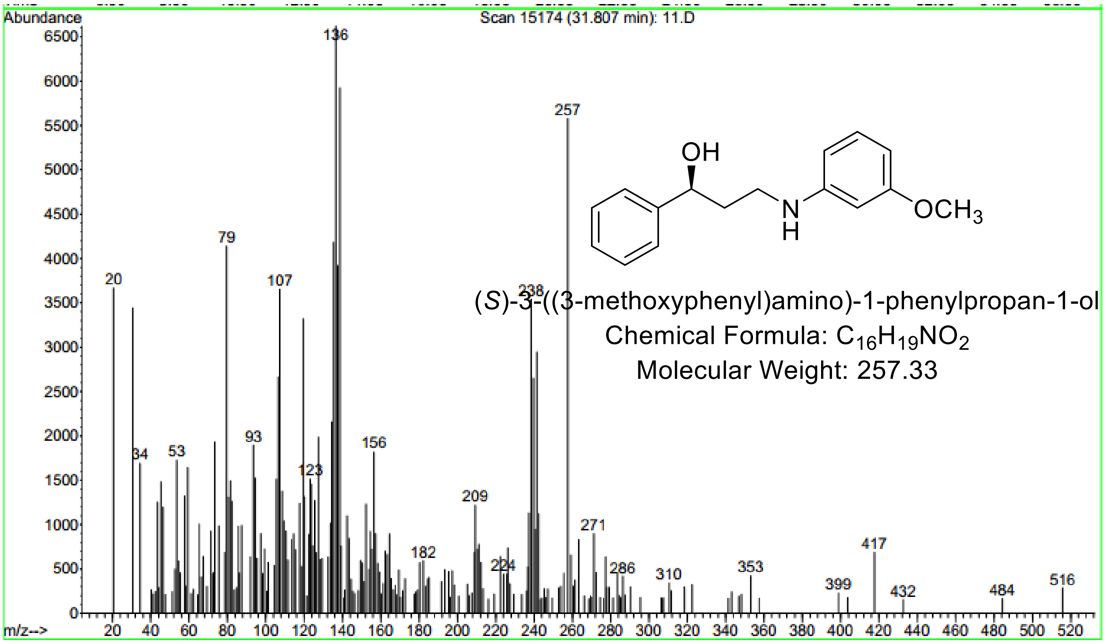

<u>3l:</u> (*S*)-1-(4-fluorophenyl)-3-(phenylamino)propan-1-ol

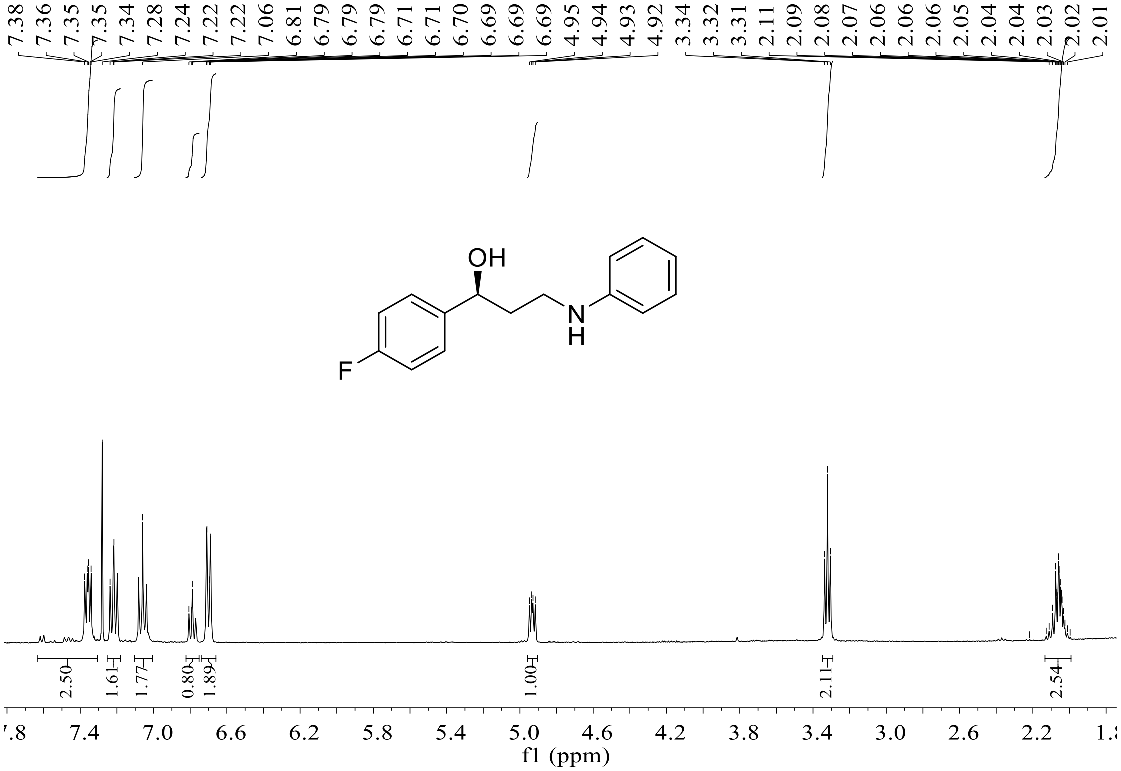

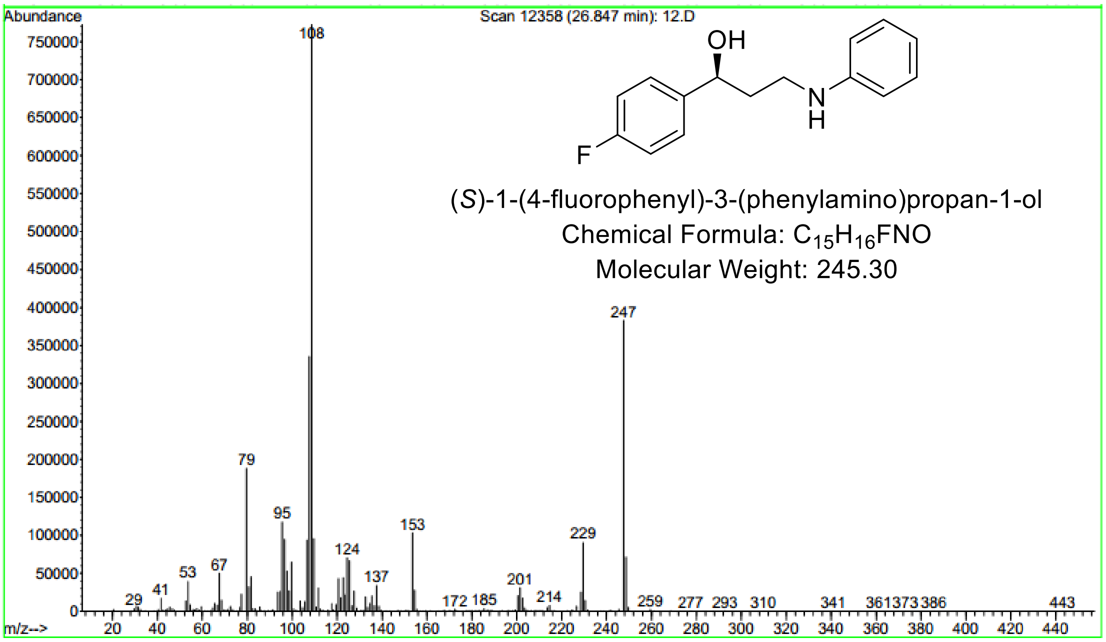

3m: (*S*)-1-(4-chlorophenyl)-3-(phenylamino)propan-1-ol

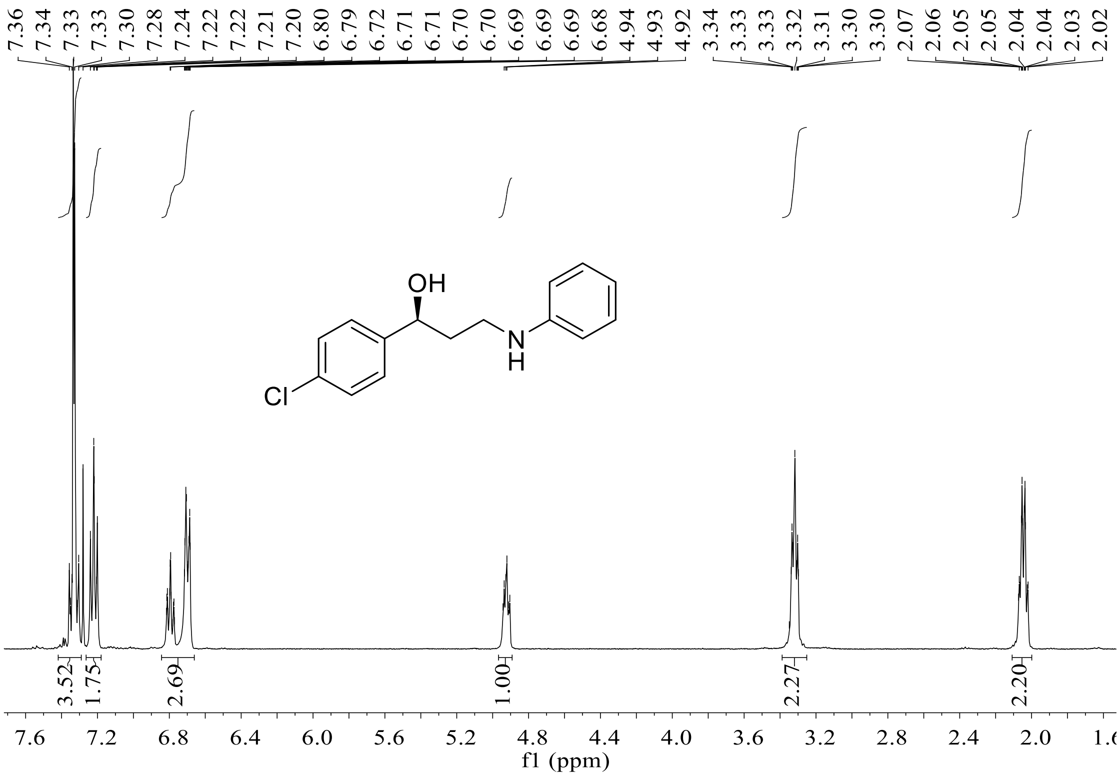

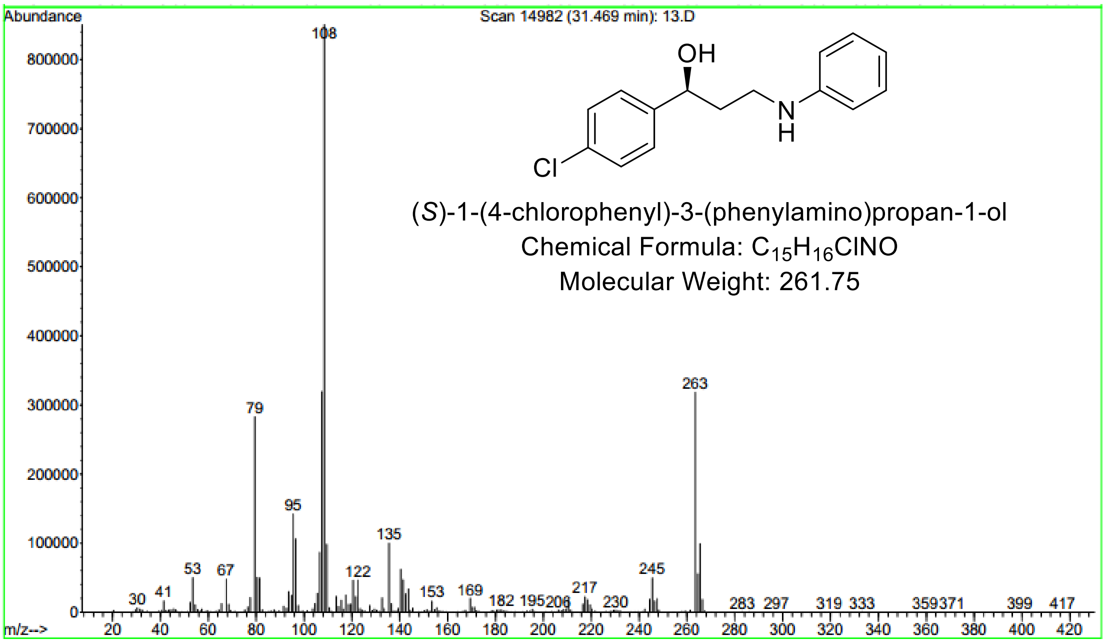

3n: (*S*)-1-(4-bromophenyl)-3-(phenylamino)propan-1-ol

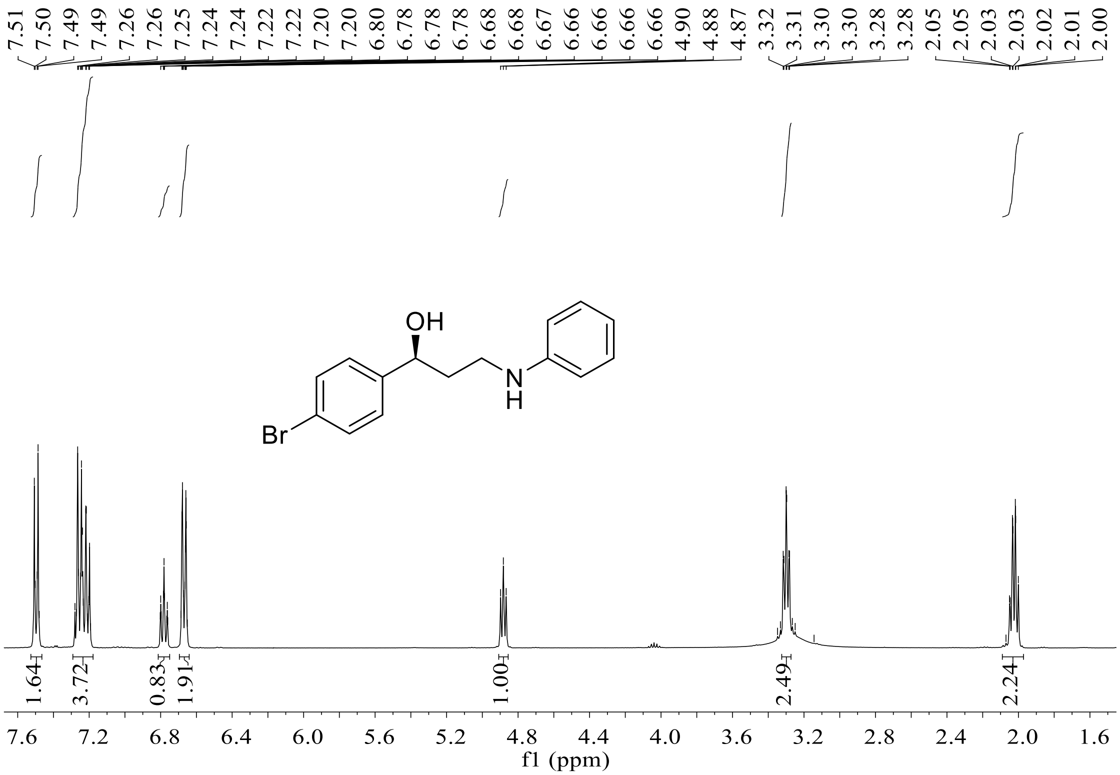

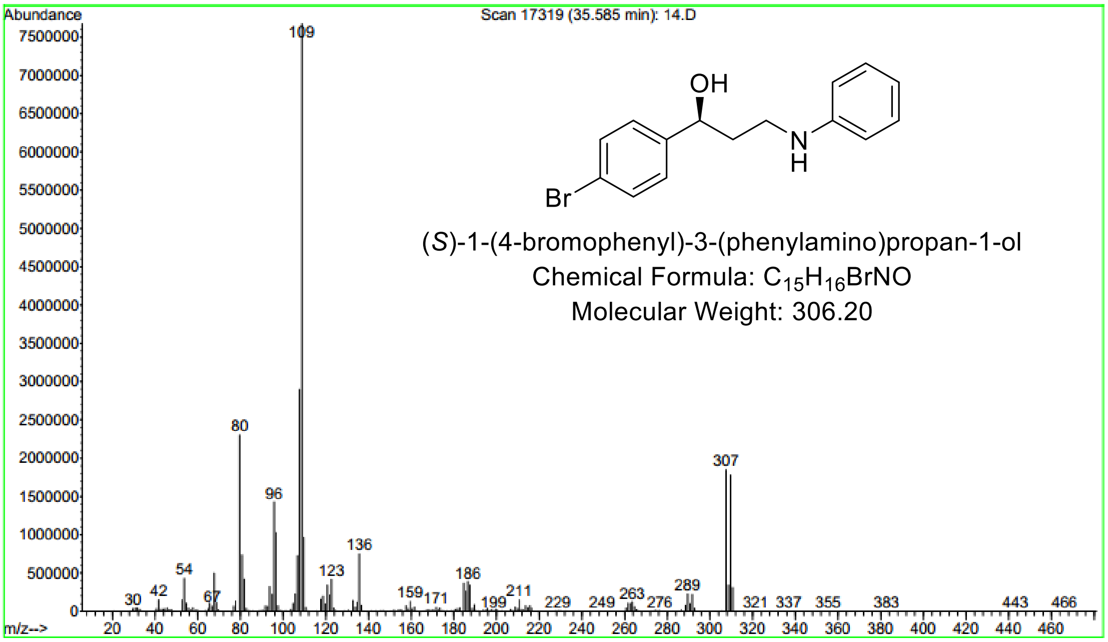

3o: (*S*)-1-(4-iodophenyl)-3-(phenylamino)propan-1-ol

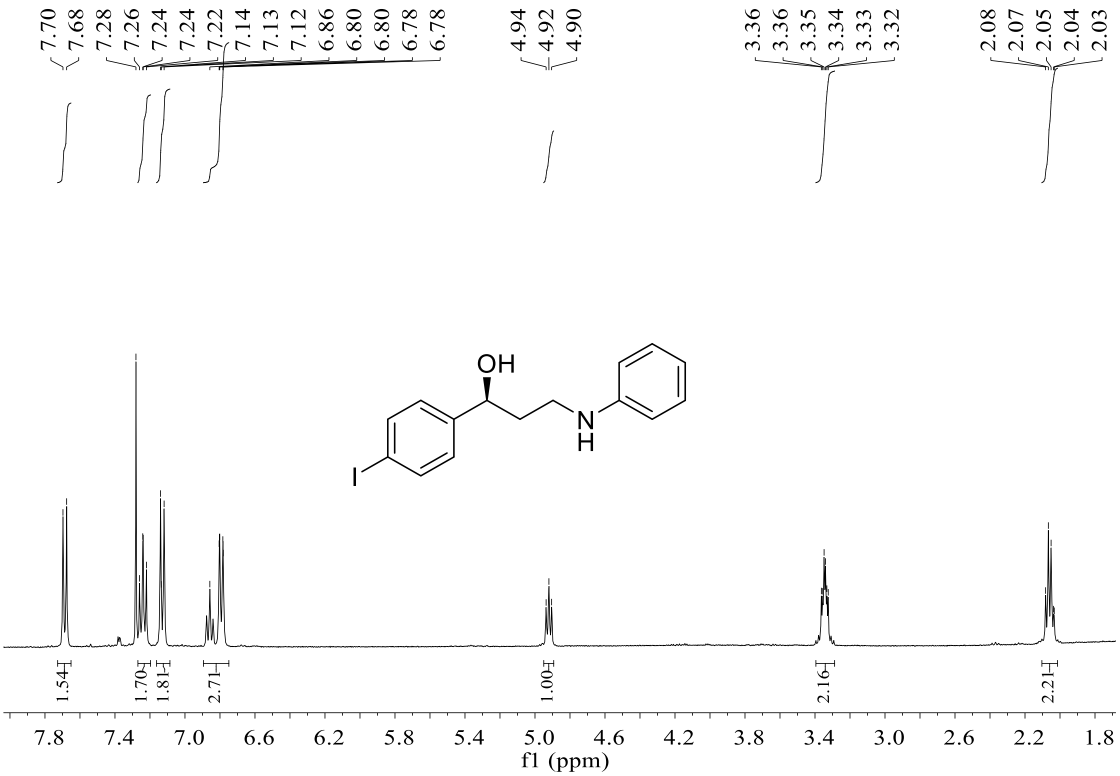

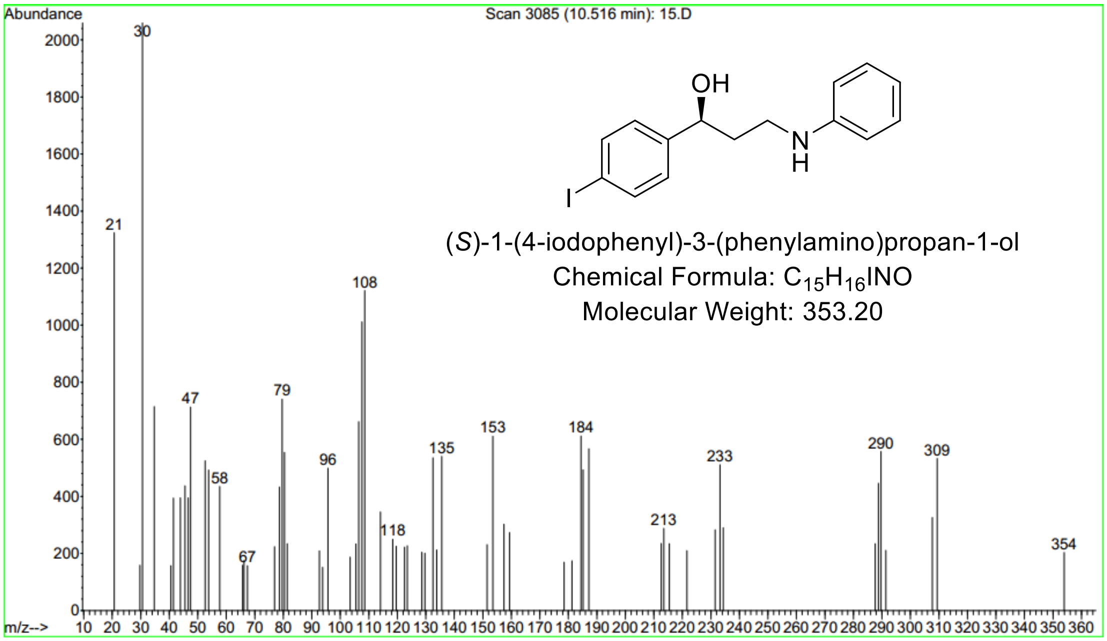

3p: (*S*)-3-(phenylamino)-1-(p-tolyl)propan-1-ol

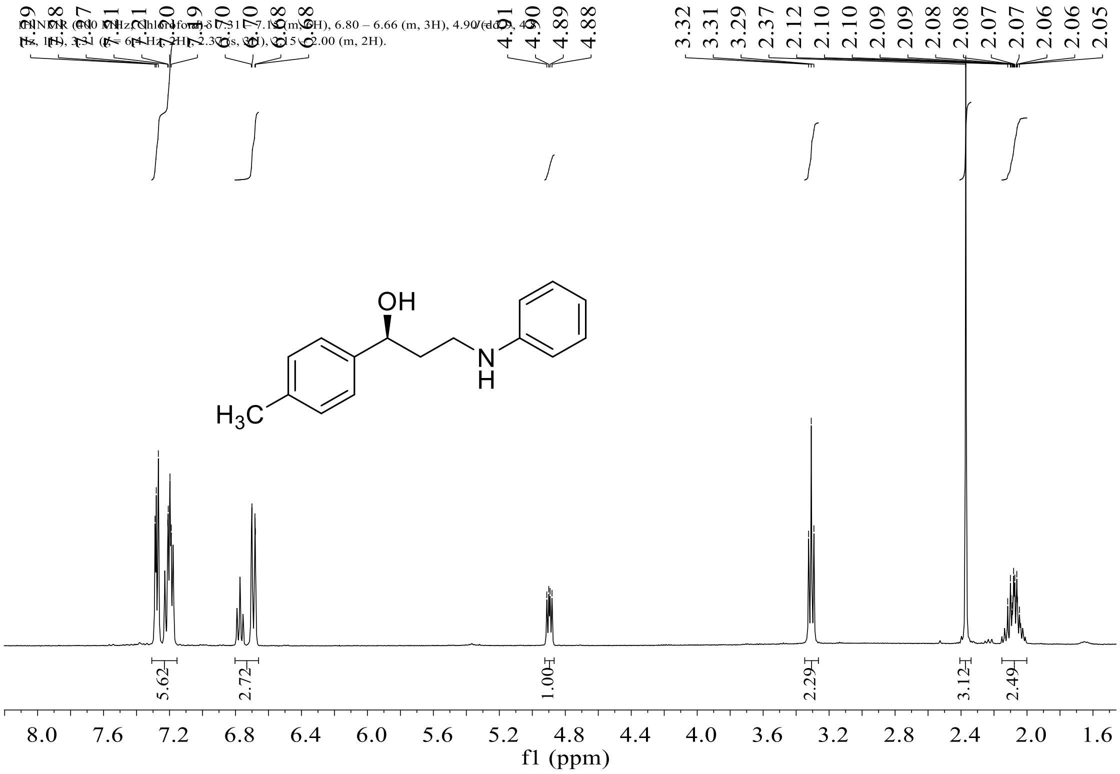

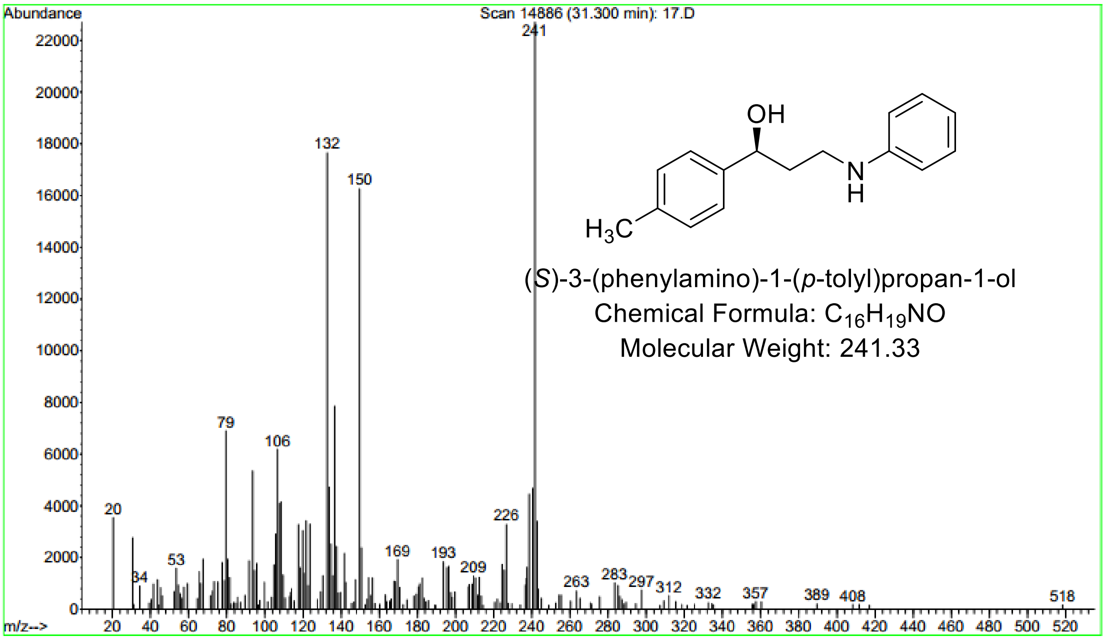

<u>3q:</u> (*S*)-1-(4-methoxyphenyl)-3-(phenylamino)propan-1-ol

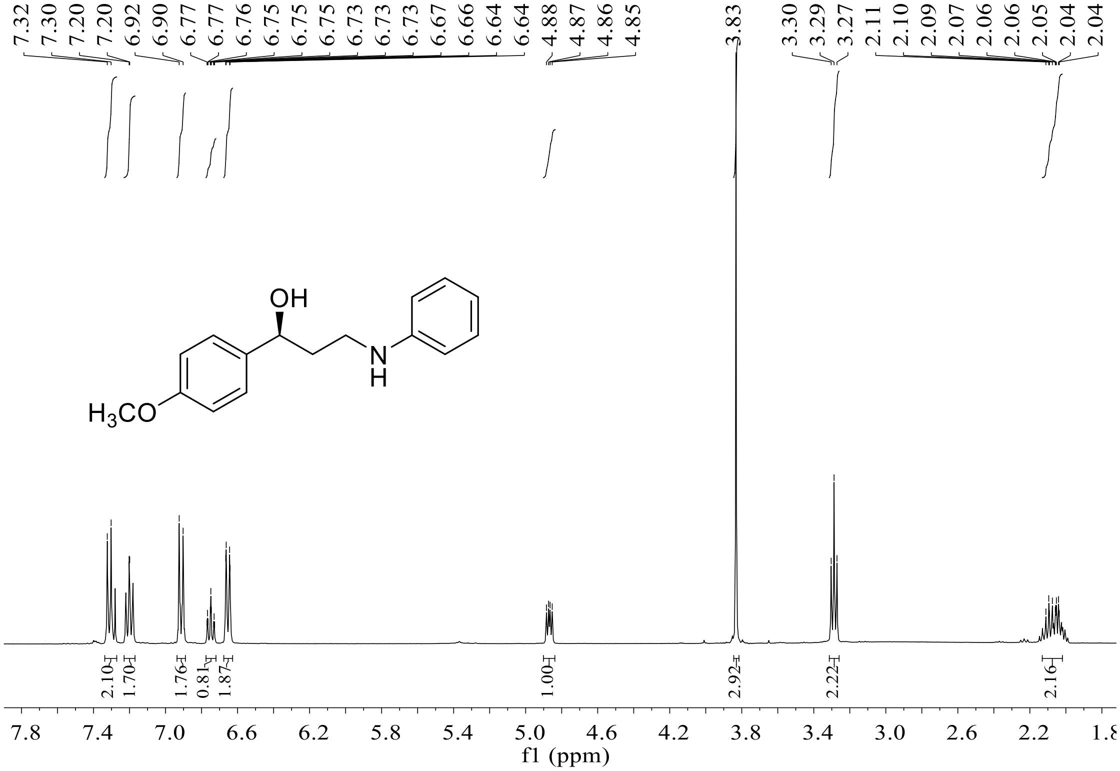

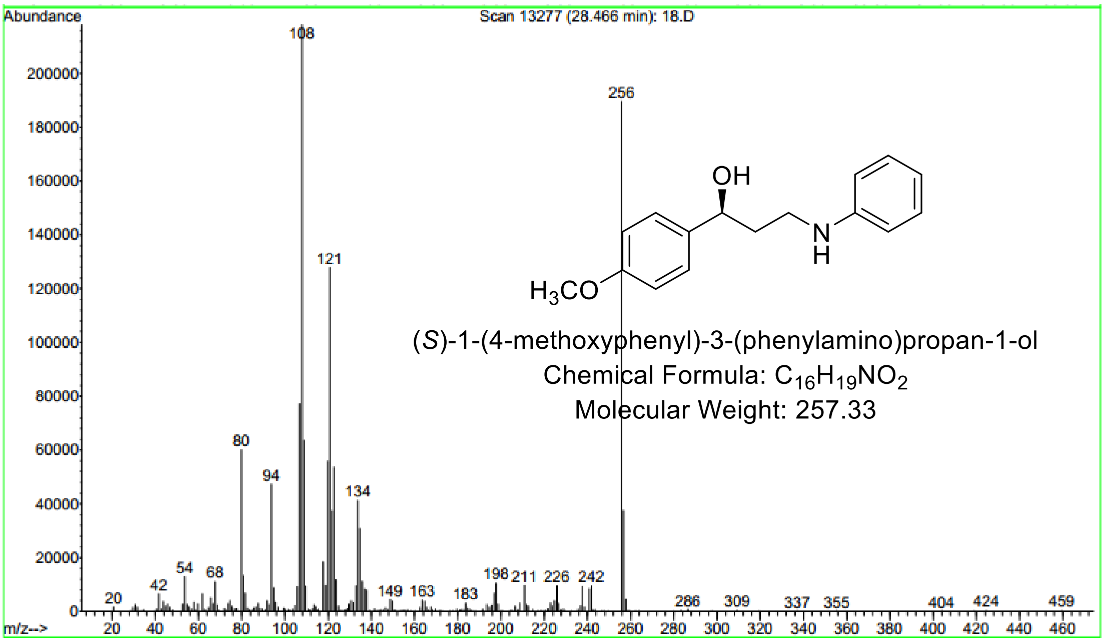

Supplement: Supplementary file 1 [file Presentation_1.PDF]
